# A clinically relevant gene signature in triple negative and basal-like breast cancer

**DOI:** 10.1186/bcr3035

**Published:** 2011-10-06

**Authors:** Achim Rody, Thomas Karn, Cornelia Liedtke, Lajos Pusztai, Eugen Ruckhaeberle, Lars Hanker, Regine Gaetje, Christine Solbach, Andre Ahr, Dirk Metzler, Marcus Schmidt, Volkmar Müller, Uwe Holtrich, Manfred Kaufmann

**Affiliations:** 1 Department of Obstetrics and Gynecology, J. W. Goethe-University, Theodor-Stern-Kai 7, Frankfurt, 60590, Germany; 2 Department of Obstetrics and Gynecology, University of Muenster, Albert-Schweitzer Straße 33, 48149, Muenster, Germany; 3 Department of Breast Medical Oncology, The University of Texas M.D. Anderson Cancer Center, PO Box 301439, Houston, TX 77230-1439, USA; 4 Department of Biology II, Ludwig-Maximilians-University Munich, Grosshaderner Str. 2, Planegg-Martinsried, 82152, Germany; 5 Department of Obstetrics and Gynecology, J. Gutenberg-University, Langenbeckstr. 1, Mainz, 55131, Germany; 6 Department of Obstetrics and Gynecology, University of Hamburg, Martinistrasse 52, Hamburg, 20246, Germany

## Abstract

**Introduction:**

Current prognostic gene expression profiles for breast cancer mainly reflect proliferation status and are most useful in ER-positive cancers. Triple negative breast cancers (TNBC) are clinically heterogeneous and prognostic markers and biology-based therapies are needed to better treat this disease.

**Methods:**

We assembled Affymetrix gene expression data for 579 TNBC and performed unsupervised analysis to define metagenes that distinguish molecular subsets within TNBC. We used *n *= 394 cases for discovery and *n *= 185 cases for validation. Sixteen metagenes emerged that identified basal-like, apocrine and claudin-low molecular subtypes, or reflected various non-neoplastic cell populations, including immune cells, blood, adipocytes, stroma, angiogenesis and inflammation within the cancer. The expressions of these metagenes were correlated with survival and multivariate analysis was performed, including routine clinical and pathological variables.

**Results:**

Seventy-three percent of TNBC displayed basal-like molecular subtype that correlated with high histological grade and younger age. Survival of basal-like TNBC was not different from non basal-like TNBC. High expression of immune cell metagenes was associated with good and high expression of inflammation and angiogenesis-related metagenes were associated with poor prognosis. A ratio of high B-cell and low IL-8 metagenes identified 32% of TNBC with good prognosis (hazard ratio (HR) 0.37, 95% CI 0.22 to 0.61; *P *< 0.001) and was the only significant predictor in multivariate analysis including routine clinicopathological variables.

**Conclusions:**

We describe a ratio of high B-cell presence and low IL-8 activity as a powerful new prognostic marker for TNBC. Inhibition of the IL-8 pathway also represents an attractive novel therapeutic target for this disease.

## Introduction

Different molecular subtypes of breast cancer have been described [1]. The most profound effects on gene expression profiles in breast cancer are related to estrogen (ER), and proliferation status, and to a lesser extent to Human Epidermal Growth Factor Receptor 2 (HER2) status. Not surprisingly, molecular classification and current prognostic signatures mainly reflect these molecular features [2]. However, substantial clinical and molecular heterogeneity remains within current molecular subsets, particularly among ER, progesterone (PgR) and HER2 receptor negative (that is, triple negative breast cancers, TNBC [3]). Furthermore the relationship between clinically defined TNBC and the gene expression profile-based basal-like breast cancer subtype (BLBC) [4] is not fully defined [5]. Some authors use these two terms synonymously given the substantial overlap between the two definitions [6,7]. However, immunohistochemical and molecular profiling studies have shown that only a subset of TNBC express the combination of basal cell markers (for example, CK5 and CK14) that is required for the molecular definition of this disease [5]. The prognostic significance and therapeutic implications of molecular heterogeneity within TNBC remains to be established. From a clinical point of view, further understanding of TNBC is important because better prognostic markers and new treatments are needed [8].

The goal of this analysis was to assemble all currently available TNBC gene expression datasets generated on Affymetrix gene chips and search for molecular structures in the data to define gene expression-based subsets within TNBC. We defined metagenes as the average expression of groups of highly co-expressed genes in the data without considering any clinical outcome variable. These metagenes identified several molecular subsets within TNBC, some with good prognosis even in the absence of systemic therapy. Our results also suggest possible new therapeutic strategies for TNBC. This study represents the largest attempt to define clinically important molecular subsets within TNBC [9].

## Materials and methods

All analyses were performed according to the REporting recommendations for tumour MARKer prognostic studies (REMARK) recommendations for prognostic and tumor marker studies [10,11] and the respective guidelines to microarray-based studies for clinical outcomes [12]. A respective diagram of the complete analytical strategy and the flow of patients through the study, including the number of patients included in each stage of the analysis, is given in Additional file 1, Supplementary Figure S1. Tissue samples of invasive breast cancer cases (dataset Frankfurt) were obtained with IRB approval and informed consent from consecutive patients undergoing surgical resection between December 1996 and July 2007 at the Department of Gynecology and Obstetrics at the Goethe-University in Frankfurt. Gene expression data have been deposited into the GEO database (accession number GSE31519).

### Assembly of TNBC microarray data and definition of metagenes

In order to facilitate pooling of data sets from different laboratories we only used data from a single platform (Affymetrix U133A and U133 Plus 2.0 chips) and included only samples that were defined as triple negative based on the mRNA expression of ER, PgR, and HER2 as previously described [13-15]. To obtain a large enough sample size for discovery it was necessary to pool several datasets. A major concern during this exercise is the possible confounding effect of systematic technical differences that exist between individual datasets. These could lead to false discovery during metagene definition and could also weaken the power of validation. We applied two different strategies to minimize this problem. First, we selected only highly comparable datasets for discovery. We initially identified 579 TNBC from a total of 3,488 publicly available primary breast cancer gene expression profiles representing 28 individual datasets (Additional file 2, Supplementary Table S1). We excluded 13 datasets contributing 185 TNBC cases from the discovery cohort because they did not fulfill our criteria of comparability of the microarray data (for details see Additional file 4, Supplementary Methods Section 1 and Additional file 1, Supplementary Figure S2). The final discovery cohort to identify metagenes included 394 TNBC from 15 datasets (cohort-A). The 185 samples excluded from discovery were retained as a validation set (cohort-B) to assess correlations between various metagenes and between metagenes and clinical outcome (Additional file 1, Supplementary Figure S1). This strategy maximized the integrity of metagene discovery at the cost of possibly reducing the power of the validation study. The two cohorts did not significantly differ with respect to age, tumor size and histological grade. However, the validation cohort-B contained a larger number of lymph node positive patients and a higher proportion of fine needle aspiration (FNA) samples. Follow-up data were available for 2,348 of the total 3,488 samples and 327 of the 579 TNBC samples. Since the number of patients with follow-up in validation cohort B was too small (*n *= 30 of 185) an additional independent validation cohort-C [16] (*n *= 76) was included to assess the prognostic value of the metagenes (Additional file 1, Supplementary Figure S1). The patient characteristics of the discovery and validation cohorts are given in Table 1. For analysis of normal tissue a dataset from a benign breast was used (Additional file 2, Supplementary Table S1).

**Table 1:** Clinical data of TNBC patients from the finding-cohort-A and the validation cohorts-B and -C

Parameter	Status	Finding cohort-A (*n *= 394)	Validation cohort-B (*n *= 185)	P-value (Chi^2^) B vs A	Validation cohort-C (*n *= 76)	*P*-value (Chi^2^) C vs A
Lymph node status	LNN	240	36		44	
	Node pos.	68	60	**< 0.001**	32	**0.001**
	*n.a*.	*86*	*89*		*0*	
Age	≤ 40 yr	63	25		10	
	41 to 50 yr	91	41		17	
	51 to 60 yr	76	39		13	
	> 60 yr	79	35	0.87	36	**0.003**
	*n.a*.	*85*	*45*		*0*	
Tumor size	≤ 2 cm	85	29		11	
	> 2 cm	224	122	0.068	62	**0.035**
	*n.a*.	*85*	*34*		*3*	
						
Histological grade	grade 3	227	110		62	
	grade 1 and 2	82	46	0.57	14	0.18
	*n.a*.	*85*	*29*		*0*	
Biopsy method	surgical	346	130		76	
	core	19	22		0	
	FNA	29	33	**< 0.001**	0	**0.009**
Five-year DFS	no event	202	24		49	
	event	95	6	0.25	26	0.69
	*n.a*.	*97*	*155*		*1*	

Unsupervised analysis, without input of clinical variables, was performed to identify metagenes that were defined as the arithmetical average expression of highly correlated genes. Gene clusters were selected with either a minimal membership of 10 genes and a minimal correlation threshold of 0.7, or a minimum of 25 genes and a correlation of 0.6, respectively (for details see Additional file 4, Supplementary Methods Section *2*). We also employed a screen to remove genes that showed data-set bias. The dependence of the expression levels of the metagene probesets on the dataset vector was analyzed using the Kruskal-Wallis statistic (Additional file 4, Supplementary Methods Section 3). Only *Stroma *and *Hemoglobin *metagenes displayed a bias for FNA samples that reflect frequent contamination of these types of samples with blood and the lack of stromal elements compared to core needle or surgical biopsies (Additional file 1, Supplementary Figure S3 and Additional file 4, Supplementary Methods). Therefore, these two metagenes were analyzed only in surgical biopsies.

No systematic bias was observed between the U133A and U133 Plus2.0 arrays, which differ only in the spatial feature size of the probesets (for details see Additional file 4, Supplementary Methods Section 4). Both metagene distributions and "Centroid methods" were used to classify subtypes of TNBC as given in Additional file 4, Supplementary Methods Sections 8 and 9).

### Survival analysis

Relapse free survival (RFS) was preferentially used as a clinical endpoint for event free survival (EFS). Only if RFS was not available in some datasets was it replaced by distant metastasis free survival (DMFS). Details on used endpoints, Kaplan-Meier and Cox regression analysis are given in Additional file 4, Supplementary Methods Section 5. Optimized cutoffs for dichotomizing of metagene scores to plot survival curves were derived from the discovery cohort and were applied without modification to the validation cohorts (Additional file 4, Supplementary Methods Section 6). All *P*-values are two-sided and 0.05 was considered as a significant result. Analyses were performed using the R software [17] and SPSS version 17.0 (SPSS Inc. Chicago, IL).

## Results

### Identification of subsets of TNBC based on metagene expression profile

**Figure 1:**
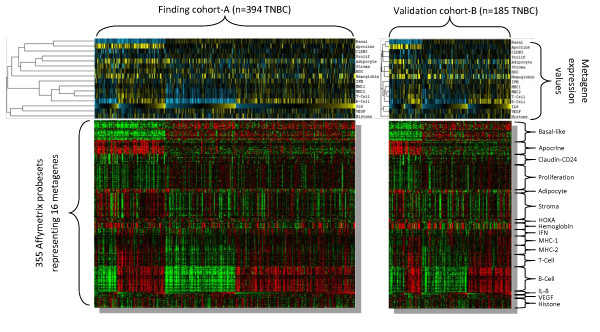
**Principal biological phenotypes identified as metagenes among TNBC**. Heatmaps of expression values of the 16 metagenes (upper panels) and the 355 individual Affymetrix probe sets (lower panels) are shown for the finding cohort (left panels, *n *= 394) and validation cohort (right panels, *n *= 185). The dendrogram at the left presents the results from hierarchical clustering of the metagenes. Three major clusters were observed representing (i) *basal-like*, *apocrine*, *CLDN-CD24*, *proliferation*, and *adipocyte *metagenes (ii) all five immune cell metagenes, and (iii) the IL-8 and VEGF metagenes, when the hemoglobin and stroma metagenes were left out which display some dataset-bias (see methods). In keeping with these three major phenotypes the samples were sorted according to (1.) Basal-like phenotype, (2.) low vs. high B-Cell metagene, and (3.) the expression value of the IL-8 metagene. (The 355 individual Affymetrix probesets and the respective metagenes are listed in the Additional file 4, Supplementary Methods).

**Figure 2:**
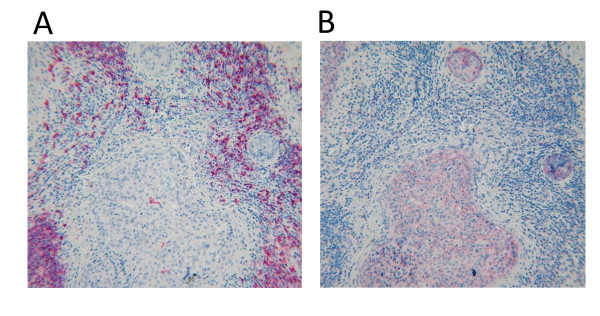
**Immunohistochemical analyses of the cellular source of expression of the B-Cell and IL-8 metagenes in TNBC**. **A) **Detection of B-lymphocytes by a CD20 antibody (red staining) in a triple negative breast cancer from the Frankfurt cohort with high expression of B-Cell and IL-8 metagenes. **B) **An adjacent section of the same tumor as in (A) is stained with an IL-8 antibody demonstrating that carcinoma cells are the source of IL-8 expression (red staining). Note the strong IL-8 staining in rod-like structures in the carcinoma cells. Further analyses using antibodies specific for macrophages (CD68) also demonstrated that macrophages are not the cellular source of IL-8 expression in the tumor (Additional file 1, Supplementary Figure S15).

In our discovery cohort we identified 16 clusters of correlated genes by unsupervised methods whose expression values were averaged as metagenes (Figure 1). As expected, no cluster of genes correlated with ER, PgR, and HER2 status [4] were identified. In contrast the identified metagenes presented in Table 2 included the basal-like phenotype [4], an apocrine/androgen receptor signaling signature [18,19], five signatures related to different types of immune cells [4,20-25], a stromal signature [26,27], the claudin-CD24 signature [28,29], markers of blood [30] and adipocytes [4], as well as an inflammatory signature [31-33] and an angiogenesis signature [23,34]. These phenotypes corresponded to previously described gene signatures that have also been used to define subsets of TNBC in a recent smaller study [9]. The angiogenesis signature (VEGF metagene) has been described very recently as a "hypoxia signature" associated with poor outcome and expressed in distant metastases [34]. As shown in Figure 1, we observed the highest correlation between different types of immune cell metagenes. Similar relationships between the metagenes were detected in the validation cohort-B (Figure 1) and -C (Additional file 1, Supplementary Figure S4). The presence of B-lymphocytes in the tumor is the primary source of the expression of the B-Cell metagene that is largely composed of immunoglobulin genes [20,22]. In contrast, immunohistochemical analyses of IL-8 expression and analysis of gene expression data of breast cancer cell lines indicate that carcinoma cells are the main source of the IL-8 metagene (Figure 2).

**Table 2:** Principal biological phenotypes identified as metagenes among TNBC

Biological component	Metagene name	Correlation within metagene cluster	# of probesets in metagene cluster	Key markers	Reference
**Basal-like phenotype**	Basal-like	0.61	37	KRT-5,-6, -14, -17, SOX10, SFRP1, ELF5, EPHB3, GABRP	[4]
**Apocrine/androgen receptor signalling**	Apocrine	0.67	27	AR, FOXA1	[18,19]
**Immune system:**					[4,20,21,23-25]
• **B-Cell**	B-Cell	0.87	48	IgG	
• **T-Cell**	T-Cell	0.84	27	TCR, LCK, ITK	
• **MHC class II**	MHC-2	0.83	14	HLA-DR, -DM, -DP, -DQ	
• **MHC class I**	MHC-1	0.84	17	HLA-A, -B, -C, -E, -F, -G	
• **Interferone response**	IFN	0.76	14	OAS1, OAS2, OAS3, MX1	
Stroma*	Stroma	0.83	47	Decorin, Osteonectin, Fibronectin, COL5A1	[26,27]
**Claudin-CD24 signature**	Claudin-CD24	0.70	19	CLDN3, CLDN4, CD24, ELF3	[28,29]
**Proliferation**	Proliferation	0.74	47	BUB1, CDC2, STK6, BIRC5, TOP2A,	[35]
Blood *	Hemoglobin *	0.63	17	HBA1, HBA2, HBB	[30]
**Adipocytes**	Adipocyte	0.74	8	FABP4, PLIN, ADIPOQ, ADH1B	[4]
**Angiogenesis**	VEGF	0.57	7	VEGF, adrenomedullin, ANGPTL4	[34]
**Inflammation**	IL-8	0.52	4	IL-8, CXCL1, CXCL2	[31,32]
**HOXA gene cluster**	HOXA	0.52	8	HOXA-4, -5, -7, -9, -10, -11	[64]
**Histone gene cluster**	Histone	0.69	19	Histones H2A, H2B	[65]

* The *Stroma *and *Hemoglobin *metagenes displayed a bias between datasets related to different biopsy techniques (see Methods).

[AU Query: Please choose a title of no more than 15 words for Tables 3 and 4. All other information should be placed in a legend beneath each table. AU Response: Done, additional information transferred into footnotes]

### Relationship between TNBC and basal-like breast cancer (BLBC)

**Figure 3:**
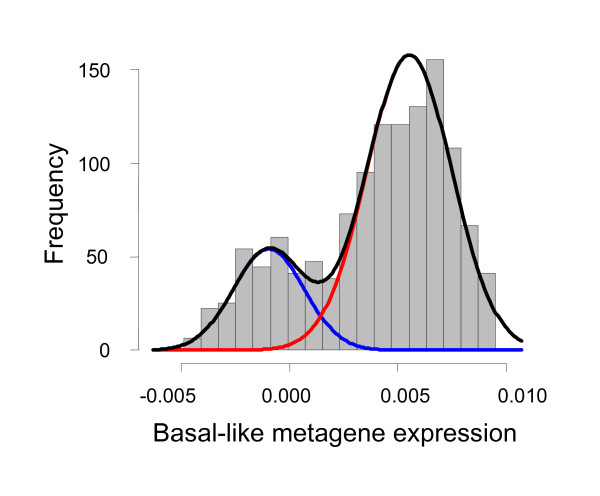
**Distribution of the expression of the basal-like metagene among TNBC of cohort-A**. The bimodal distribution of the expression of the basal-like metagene among the 394 TNBC samples in the finding cohort-A is shown. A mixture (black line) of two normal gaussian distributions (blue and red lines) was fitted to these data. The interception of the two gaussians was derived as a cutoff (0.0014) for the definition of basal-like tumors. Similar results were obtained for the validation cohorts-B, and -C, as well as from all samples combined.

We observed a clear bimodal distribution of the *basal-like *metagene score among TNBC (Figure 3). This bimodal distribution allows us to derive a cutoff to separate cases into high and low expression groups by fitting two normal distributions to the data (Figure 3). According to this cutoff, 72.8%, 73.0% and 69.7% of TNBC were defined as BLBC in the discovery cohort-A, validation cohort-B, and validation cohort-C, respectively. Table 3 compares the clinical characteristics of BLBC or non-BLBC triple negative cancers the discovery cohort-A. The positive association between high histological grade (G3, *P *< 0.001), younger age (*P *= 0.004) and BLBC were also observed in the validation cohort-C and validation cohort-B, respectively (Additional file 2, Supplementary Table S2).

**Table 3:** Clinical parameters of TNBC with basal-like breast cancer (BLBC) or non-BLBC phenotype

Parameter	Information available*		Non-BLBC (*n *= 107, 27.2%)	BLBC (*n *= 287, 72.8%)	Total (*n *= 394)	*P*-value
lymph node status	*n *= 308	LNN	50 (64.9%)	190 (82.3%)	240	
		N1	27 (35.1%)	41 (17.7%)	68	**0.002**
Age 50 yrs	*n *= 309	≤ 50 yr	27 (34.6%)	124 (53.7%)	151	
		> 50 yr	51 (65.4%)	107 (46.3%)	158	**0.004**
Tumor size	*n *= 309	≤ 2 cm	16 (20.5%)	69 (29.9%)	85	
		> 2 cm	62 (79.5%)	162 (70.1%)	224	0.14
Histological grade	*n *= 309	G3	45 (57.0%)	182 (79.1%)	227	
		G1&2	34 (43.0%)	48 (20.9%)	82	**< 0.001**

* Number of cases with available information on the specific parameter in the finding cohort-A

In unsupervised clustering of the metagenes the *basal-like *metagene clustered next to the *apocrine *metagene but showed a strong inverse correlation (Figure 1). To quantify the correlation between the *basal-like *metagene and all other metagenes from Table 2 we used quartiles of the respective metagenes. Additional file 2, Supplementary Table S3 presents the six metagenes that displayed significant correlations with the BLBC phenotype in both the discovery and validation cohorts. A positive correlation was found between the BLBC phenotype and the *proliferation *and angiogenesis (*VEGF*) metagenes. A negative correlation was observed for the apocrine/androgen receptor signaling and two immune system related metagenes (*MHC-2 *and *T-Cell *metagenes), as well as an adipocyte related signature.

Since we observed a negative correlation between the *basal-like *metagene and potential markers of normal breast tissue, such as the *adipocyte *metagene, we had to exclude the possibility that we are only distinguishing stroma-rich and stroma-poor samples. As shown in Additional file 1, Supplementary Figure S5, when metagenes for *proliferation*, *adipocytes *and *histones *were compared between BLBC, non-BLBC, and normal breast samples it is clearly demonstrated that the non-BLBC subtype is distinct from normal breast tissues in the expression of several metagenes. Proliferation genes have been previously shown to be the most important determinant of cancer vs normal signatures [35]. Furthermore, the strong bimodal distribution of the *basal-like *metagene argues against the possibility that this metagene is inversely describing the degree of contamination with normal tissue which should rather result in a continuous distribution. The non-BLBC tumors in our TNBC dataset mainly represent samples of the "molecular apocrine" type (16.5%), which demonstrates the inverse bimodal distribution as the *basal-like *metagene, and a relatively small group of "claudin-low" tumors (6.3%). The mutual relationship of these three metagenes is shown in Additional file 1, Supplementary Figure S6.

### Prognostic value of the different biological phenotypes in TNBC

**Figure 4:**
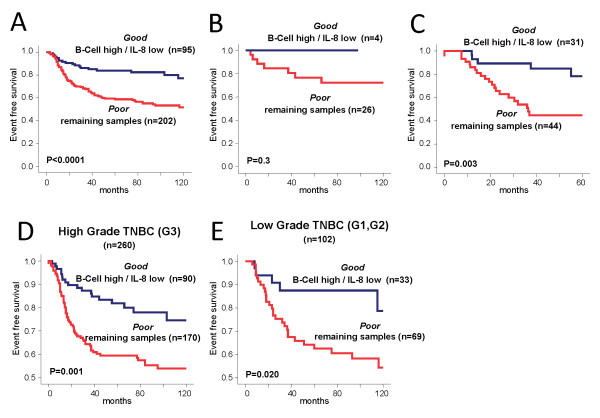
**Prognostic value of the combined B-Cell/IL-8 metagenes among TNBC**. Kaplan Meier analysis of event free survival of 297 TNBC patients with follow up from the finding cohort A. Samples were stratified according to prognostic predictor of the combined B-Cell/IL-8 metagenes. *"Good" *refers to 95 samples with both high B-Cell and low IL-8 metagene expression whereas all other samples (*n *= 202) are referred as "*Poor"*. **A) **Prognostic value of the B-Cell/IL8-metagene prognostic predictor in the 30 TNBC patients with follow up from the validation cohort-B. Samples were stratified as in (A). **B) **Prognostic value of the B-Cell/IL8-metagene prognostic predictor in the 75 TNBC patients with follow-up from the independent validation cohort-C. Samples were stratified as in (A). **C) **Prognostic value of the combined B-Cell/IL-8 metagenes among the subset of high grade (G3) TNBC tumors from all three cohorts -A, -B, and -C (*n *= 186). Samples were stratified as in (A). (Results from the individual cohorts are given in Additional file 1, Supplemental Figure S8). **D) **Prognostic value of the combined B-Cell/IL-8 metagenes among the subset of low to medium grade (G1 and G2) TNBC tumors from all three cohorts -A, -B, and -C (*n *= 77). Samples were stratified as in (A). (Results from the individual cohorts are given in Additional file 1, Supplemental Figure S8).

To assess the prognostic value of the metagenes, we analyzed the event free survival of patients as a function of metagene expression. The *basal-like *metagene had no significant effect on survival (Additional file 1, Supplementary Figure S7). In contrast, five other metagenes including the *IL-8, Histone, VEGF, B-Cell*, and *T-Cell *metagenes showed significant prognostic values when considered as continuous variables in univariate analysis (Additional file 2, Supplementary Table S4). In a stepwise multivariate Cox regression analysis only three of these, the *IL-8, Histone*, and the *B-Cell *metagenes, remained significant (Additional file 2, Supplementary Table S5). The *IL-8 *and *Histone *metagenes were positively correlated with one another in all data sets (see Figure 1). The *B-cell *and *IL-8 *metagenes were associated with prognosis but with an opposing direction. Based on these observations, we derived a *B-Cell */*IL-8 *metagene ratio as a prognostic index for TNBC. Figure 4A demonstrates that patients with a high expression of the *B-Cell *and low expression of the *IL8 *metagene have significantly better prognosis than other TNBC patients (HR 0.37, 95% CI 0.22 to 0.61; *P *< 0.001). The five-year event-free survival was 84 ± 4% for the good prognosis group (*n *= 95) compared to 59 ± 4% for the rest of the patients. In validation cohort B (*n *= 30), there was a non-significant trend for better survival for patients with high B-cell low IL8 metagene expression (*P *= 0.3, Figure 4B). Since this cohort has limited power due to the small sample size, we also tested the prognostic value on a separate and larger (*n *= 75) validation cohort of TNBC samples [16]. The B-cell/IL8 metagene ratio had significant prognostic value in this second validation cohort C, the hazard ratio (HR) was 0.26, (95% CI 0.10 to 0.68) and the five-year DFS was 78 ± 9% vs. 45 ± 8%, (*P *= 0.003) (Figure 4C). The prognostic value was independent of histological grade; Figure 4D, E shows pooled data from all three cohorts to increase sample size, (see also Additional file 1, Supplementary Figure S8 for the individual cohorts). Moreover, the prognostic value of the B-cell/IL8 metagene ratio was observed both in BLBC and non-BLBC TNBCs (*P *= 0.001 and *P *= 0.006, respectively; Additional file 1, Supplementary Figure S9). The proportion of BLBC cases was similar in the Good and Poor prognosis groups defined by the B-cell/IL8 metagene ratio (75.2% and 71.8%, respectively; *P *= 0.54).

To assess a potential predictive value for sensitivity to systemic adjuvant chemotherapy, the patients were stratified by adjuvant treatment. In the discovery cohort, 186 patients received no adjuvant systemic treatment and 81 patients received chemotherapy (mostly Cyclophosphamide Methotrexate Fluorouracil; CMF)). Better prognosis was observed for the high B-cell/low IL8 group in both untreated (*P *= 0.001) as well as chemotherapy treated patients (*P *= 0.05; not shown). A potential predictive value of the B-cell and IL8 metagenes was also analyzed in 191 patients with TNBC who received neoadjuvant chemotherapy. We assembled this cohort of samples with information on pathologically complete response (pCR) from seven datasets. As shown in Additional file 1, Supplementary Figure S10 the B-cell metagene had a modest predictive value with an area under the curve (AUC) of 0.606 consistent with our previous results [22]. The predictive value for the IL8 metagene was smaller (AUC -0.552). Combining both metagenes increased the AUC to 0.612 (95% CI 0.519 to 0.704; *P *= 0.018).

In multivariate Cox regression analysis, including lymph node status, age, tumor size, and histological grade, only the combined B-Cell/IL8-metagene score showed strong independent prognostic value in both the discovery cohort (HR 0.38, 95% CI 0.22 to 0.67, *P *= 0.001) and in the second, larger validation cohort-C, (HR 0.21, 95% CI 0.07 to 0.62, *P *= 0.005). The only other variable with borderline statistical significance (HR 0.40; 95% CI 0.17 to 0.99, *P *= 0.046) was lymph node status in validation cohort-C (Table 4). However, even in univariate analyses the remaining clinical variables did not show a significant prognostic value in the analyzed cohorts. This might be attributed to the fact that most TNBC are usually highly proliferating and grading is not as important for prognosis in this subtype as it is in ER positive disease; in addition, the power of our analysis may be limited to detecting the modest effect of age and tumor size on prognosis within this sample set. The inclusion of a term for chemotherapeutic treatment in the multivariate analysis further reduced the sample size to 213 patients in cohort-A (no treatment information was available for patients from validation cohort-B). Of these 213 patients only 37 were treated with chemotherapy. The combined B-Cell/IL8-metagene score remained significant (*P *= 0.001) in the corresponding multivariate analysis (Additional file 2, Supplementary Table S9A). Unexpectedly, chemotherapy treatment was associated with a worse prognosis probably due to chance or some form of selection bias to include higher risk patients in these public data sets (Additional file 2, Supplementary Table S9A). This selection bias is consistent with a significant higher portion of node positive patients in the chemotherapy group (*P *= 0.001) and a trend for a higher histological grade (*P *= 0.074; Additional file 2, Supplementary Table S9B).

**Table 4:** Multivariate analysis of EFS according to standard parameters and the combined B-Cell/IL8-metagene in TNBC

		Finding cohort A*				Validation cohort C*			
			
Variable		No. of patients^†^	Hazard ratio	95% CI	***P*-value**^‡^	No. of patients^§^	Hazard ratio	95% CI	***P*-value**^‡^
Lymph node status	LNN vs N1	210 vs 27	0.59	0.31 to 1.12	0.10	43 vs 29	0.40	0.17 to 0.99	**0.046**
Age	> 50 vs ≤ 50	113 vs 124	0.75	0.48 to 1.17	0.21	48 vs 24	1.68	0.65 to 4.38	0.29
Tumor size	≤ 2 cm vs > 2 cm	71 vs 166	0.73	0.44 to 1.21	0.22	11 vs 61	0.99	0.28 to 3.42	0.98
Histological grading	G3 vs G1 and 2	166 vs 71	1.11	0.68 to 1.81	0.68	59 vs 13	0.53	0.22 to 1.29	0.16
B-Cell/IL8-Signature	*Good *vs *Poor*^||^	78 vs 159	0.38	0.22 to 0.67	**0.001**	29 vs 43	0.21	0.07 to 0.62	**0.005**

* Results from multivariate Cox analysis of event free survival in the TNBC finding cohort A and validation cohort C are presented.

† information on all parameters was available for 237 of the 297 TNBC samples with follow up data from the finding cohort A.

‡ Significant *P*-values are given in bold

§ information on all parameters was available for 72 of the 76 TNBC samples with follow up data from the validation cohort C.

|| "*Good*" refers to high *B-Cell *metagene together with low *IL8 *metagene expression compared to all the remaining samples referred as "*Poor*".

### Relationship of the identified metagenes to known prognostic signatures

The correlation of several published prognostic gene signatures to the metagenes discovered within the pure TNBC cohort was analyzed by hierarchical clustering using the gene expression data from cohort-A (Additional file 4, Supplementary Methods Section 13). As shown in Additional file 1, Supplementary Figure S11, the "recurrence score" [36], "genomic grading index" (GGI) [37], and the "wound response signature" [38] display high correlation to the proliferation metagene. On the other hand the "7-gene immune response (IR) signature" [39], the "stroma derived prognostic predictor" (SDPP) [40], and the "368 gene medullary breast cancer signature" [16] were all highly correlated to immune cell metagenes. The magnitude of the correlation (R^2 ^= 0.4 to approximately 0.7) between the different immune metagenes and the related signatures is at the same high level as the correlation between genes within other metagene clusters (R^2 ^= 0.5 to approximately 0.7; Table 2). We demonstrated previously [22] that even if the different immune metagenes can discriminate between distinct types of immune cells, the actual infiltration of tumors generally represents a mixture of these different immune cells. In most cases, the differences in the proportions in this mixture are smaller than the global differences in lymphocyte infiltration between individual tumors. Therefore, different immune signatures often carry redundant prognostic information and can replace each other. In contrast to the immune cell metagenes no correlation between the IL8 metagene and other signatures were observed.

## Discussion

It has been suggested that TNBC represent a group of several molecularly [3] and clinically [41,42] distinct disease subtypes. We used gene expression data of a cohort of 394 TNBC to identify molecular subsets within this tumor type. The definition of TNBC was based on gene expression data which is not the standard definition used in the clinic. This might be a caveat but holds the promise that samples erroneously characterized as receptor-negative by immunohistochemistry do not introduce noise into our analysis. We identified 16 metagenes associated with several distinct biological processes that showed variable expression across TNBC (Table 2). Some of the metagenes seem to point to the distinct origins of these cancers [43,44]. These include the basal-like [4], the apocrine [18,19], and the claudin-low [28,29] subtypes of TNBC. Other metagenes were related to non-neoplastic cellular constituents of the tumor microenvironment including stroma [26,27], blood cell [30] and adipocytes [4], as well as signatures for angiogenesis [23,34] and inflammation [31-33]. Five metagenes appear to reflect the variable presence of immune cells and may contribute to the clinical behavior of the cancer [4,20-25,27,45] (Table 2).

Kreike *et al. *[9] detected similar metagenes among 97 TNBC analysed with a different microarray platform. That study suggested that the TNBC clinical phenotype can be equated to the BLBC molecular class determined by the centroid method [46] since 95% of the TNBCs were assigned *basal-like *molecular class [47]. However, the centroid method is highly susceptible to the composition of the dataset that is used to define the reference centroids [48] and variants of the method can lead to different results [49]. Bertucci *et al. *[50] identified only 71% of their 172 TNBC cases as *basal-like *when using a slightly different version of the centroid method for molecular classification. When we applied different versions of the centroid method to 1,364 breast cancers, 65% to 90% of the TNBC samples (*n *= 172) were assigned to the basal-like class depending on the method used (Additional file 2, Supplementary Table S6). In this paper we took a different approach and first identified metagenes and used these metagenes to define molecular subsets among TNBC. One of our metagenes corresponded closely to the gene signatures that are used to define BLBC in the centroid based methods. Our results indicate that BLBC defined based on the *basal-like *metagene expression represent around 73% of TNBC (Table 3 and Additional file 2, Supplementary Table S2).

The proportion of BLBC among TNBC in our study is similar to results from an immunohistochemical study by Rakha *et al. *[7] that defined BLBC by the expression of CK5/6, CK14, CK17 or EGFR. These authors observed a worse survival of the 165 patients with BLBC compared to the remaining 67 TNBC cases, which expressed none of these markers. However, we did not detect differences in the prognosis of BLBC and non-BLBC type triple negative cancers (Additional file 1, Supplementary Figure S7). In the study by Rakha *et al. *the prognostic effect was mainly confined to 103 untreated patients. Still, even when we analyzed untreated patients (*n *= 186) separately, we detected no prognostic value of the BLBC phenotype (not shown). Our results are also contrary to the immunohistochemical study of Cheang *et al. *[51], which used CK5/6 and EGFR antibodies for TNBC stratification. They also observed a worse prognosis of 336 BLBC TNBC compared to 303 non-BLBC TNBC. However, our study is not directly comparable to these prior reports because our definition of BLBC is fundamentally different from the IHC-based methods. Our results are in line with several other genomic profiling studies that reported limited prognostic value for the BLBC molecular class among clinically triple negative cancers [18,19,50].

We observed strong prognostic value for several of the other metagenes (Additional file 2, Supplementary Table S4). An improved prognosis was observed for patients with tumors displaying high expression of immune system related metagenes which supports recent reports [20,23-25,27,39,40,52,53]. An association with decreased survival was observed for high expression of inflammation (IL-8), an angiogenesis/hypoxia signature (VEGF) [34], and histone-related metagenes (Additional file 2, Supplementary Table S4 and Figure 1). A simple combination of high B-Cell and low IL8 metagene expression identifies a subset of TNBC patients (32% of all) with a favorable prognosis and a five-year event-free survival of 84%. In multivariate analysis, only this metagene ratio and lymph node status were significant predictors of TNBC in our cohort of patients (Table 4 and Figure 4D, E). Other known prognostic factors in breast cancer, such as age, tumor size and histological grade, were not significant in our cohorts, even in univariate analysis. Most TNBC are high grade and, therefore, grade is not as important for prognosis in this subtype as it is in ER positive disease. TNBCs are also often associated with younger age but the impact of age and tumor size for prognosis within this subtype is not yet fully clear. Still it cannot be excluded that a bias in our cohort is the reason for the lack of the significance of these factors. Our analyses of neoadjuvant treated TNBC samples suggest modest predictive value of the B-cell/IL8 metagene ratio for currently used chemotherapies [22,54] (Additional file 1, Supplementary Figure S10). We also observed a pure prognostic value in untreated patients of finding the cohort in line with other reports on B-cell metagene [24,27]. Treatment information on the samples from the validation cohort was not available.

Our observation is important since every currently available genomic prognostic signature, (for example, the 70-gene profile [55], Recurrence Score [36], Genomic Grading Index [37]), assigns poor prognostic risk status to all TNBC samples despite their variable outcome [56-58]. One of these signatures, the Rotterdam-76-gene prognostic signature [59], was developed in a way to allow prognostic stratification of ER-negative cancers. However, similar to other reports [9] we were not able to demonstrate a prognostic value for this signature (Additional file 1, Supplementary Figure S12).

We used an unsupervised class discovery approach to first identify the main molecular subtypes within the data and then assess the prognostic differences between the molecular subsets. Interestingly, when we performed an independent supervised analysis that compared TNBC cases with or without recurrence, we also identified IL-8 as the top ranked gene associated with poor prognosis (Additional file 1, Supplementary Figure S13 and Additional file 2, Supplementary Table S8). However, gene signatures obtained through supervised analysis were not superior to the molecular structure based prognostic predictions in validation (Additional file 1, Supplementary Figure S14). In addition, the biological interpretation of the empirically derived prognostic signature is more difficult than the interpretation of metagenes. In summary, we performed the largest unsupervised analysis of pooled gene expression data from TNBC. We describe a new prognostic signature for these cancers that identify about one-third of TNBC as relatively low risk for recurrence. These cancers are characterized by high B-cell and low IL-8 metagene expression and have about 84% recurrence-free survival at five-years. Whereas, this may not be sufficiently high to forego adjuvant chemotherapy, these observations pave the way to develop a clinically useful multivariate prognostic model for TNBC. A combined, prognostic score, including clinical variables, such as nodal status and perhaps tumor size, and molecular variables, such as optimized B-cell and IL-8 metagenes (measured by an RT-PCR or array-based method), may identify patients with very low risk of recurrence even with ER-, PgR- and HER2-negative breast cancer. Equally important, the prognostic importance of B-cells and the negative impact of IL-8 suggest potential novel therapeutic strategies for TNBC that can be tested in the clinic [31,32]. It could allow the selection of those patients who could profit most from novel immune stimulating drugs like anti-CTLA-4 antibodies that have shown promise in melanoma [60,61]. IL8 could also directly increase the survival of breast cancer stem cells after chemotherapy [62], which can be blocked with IL8 directed drugs [63]. Such an effect might explain the triple negative paradox with high relapse rates despite a good initial response to chemotherapy.

## Conclusions

In the largest and most comprehensive analysis of all available gene expression data in TNBC, we first identified structures in the molecular data without considering any clinical outcome. Subsequently, these molecular phenotypes were correlated with survival in multivariate analysis, including routine clinical and pathological variables. Our most important observation is that a high B-cell presence and low IL-8 activity identifies a good prognosis group, even in the absence of systemic therapy, among TNBC. These observations directly point to therapeutic interventions, such as the inhibition of the IL-8 pathway and activation of the immune system in the tumor microenvironment that could benefit patients with this disease.

## Abbreviations

AUC: area under the curve; BLBC: basal-like breast cancer; CK: cytokeratine; DMFS: distant metastasis free survival; EFS: event free survival; EGFR: epidermal growth factor receptor; ER: estrogen receptor; FNA: fine needle aspiration; GGI: genomic grading index; HER2: human epidermal growth factor receptor 2; HR: hazard ratio; IL: interleukine; IR: immune response; MHC: major histocompatibility complex; PgR: progesterone receptor; REMARK: recommendations for prognostic and tumor marker studies; RFS: Relapse free survival; SDPP: stroma derived prognostic predictor; TNBC: triple negative breast cancer; VEGF: vascular endothelial growth factor.

## Competing interests

The authors declare that they have no competing interests.

## Authors' contributions

AR, TK and UH conceived the study, carried out the analyses and wrote the manuscript. CL and LP added experimental data, participated in the interpretation of the data and in writing the manuscript. ER, LH, RG, CS AA, MS and VM provided patients and samples, obtained follow-up data and helped to draft the manuscript. DM and TK performed the statistical analysis. MK initiated the study and participated in the design and writing of the manuscript. All authors read and approved the final manuscript.

## Supplementary Material

Additional file 1 **Supplementary Figures S1 to S15**. An Adobe file containing 15 supplementary figures (S1 to S15). Click here for file

Additional file 2 **Supplementary Tables S1 to S7**. An Adobe file containing seven supplementary tables (S1 to S7). Click here for file

Additional file 3 **Supplementary Tables S8**. An Excel file containing a supplementary table (S8) containing lists of probesets and corresponding information from the supervised analysis by SAM. Click here for file

Additional file 4 **Supplementary Methods**. An Adobe file containing supplementary information on methodology and six additional supplementary figures (S16 to S21), which are referred to within this supplementary methods. Click here for file

Additional file 5 **Supplementary R files**. A zipped package containing an R script file of the analysis with respective links to the complete dataset files in GEO and a text file of the metagene probesets used in the R analysis. Click here for file

## References

[B1] Sotiriou C Pusztai L Gene-expression signatures in breast cancer N Engl J Med 2009 360 790 800 10.1056/NEJMra0801289 19228622

[B2] Wirapati P Sotiriou C Kunkel S Farmer P Pradervand S Haibe-Kains B Desmedt C Ignatiadis M Sengstag T Schütz F Goldstein DR Piccart M Delorenzi M Meta-analysis of gene expression profiles in breast cancer: toward a unified understanding of breast cancer subtyping and prognosis signatures Breast Cancer Res 2008 10 R65 10.1186/bcr2124 18662380 PMC2575538

[B3] Gusterson B Do 'basal-like' breast cancers really exist? Nat Rev Cancer 2009 9 128 134 10.1038/nrc2571 19132008

[B4] Perou CM Sørlie T Eisen MB van de Rijn M Jeffrey SS Rees CA Pollack JR Ross DT Johnsen H Akslen LA Fluge O Pergamenschikov A Williams C Zhu SX Lønning PE Børresen-Dale AL Brown PO Botstein D Molecular portraits of human breast tumours Nature 2000 406 747 752 10.1038/35021093 10963602

[B5] Rakha EA Reis-Filho JS Ellis IO Basal-like breast cancer: a critical review J Clin Oncol 2008 26 2568 2581 10.1200/JCO.2007.13.1748 18487574

[B6] Carey LA Dees EC Sawyer L Gatti L Moore DT Collichio F Ollila DW Sartor CI Graham ML Perou CM The triple negative paradox: primary tumor chemosensitivity of breast cancer subtypes Clin Cancer Res 2007 13 2329 2334 10.1158/1078-0432.CCR-06-1109 17438091

[B7] Rakha EA Elsheikh SE Aleskandarany MA Habashi HO Green AR Powe DG El-Sayed ME Benhasouna A Brunet JS Akslen LA Evans AJ Blamey R Reis-Filho JS Foulkes WD Ellis IO Triple-negative breast cancer: distinguishing between basal and nonbasal subtypes Clin Cancer Res 2009 15 2302 2310 10.1158/1078-0432.CCR-08-2132 19318481

[B8] Gluz O Liedtke C Gottschalk N Pusztai L Nitz U Harbeck N Triple-negative breast cancer - current status and future directions Ann Oncol 2009 20 1913 1927 10.1093/annonc/mdp492 19901010

[B9] Kreike B van Kouwenhove M Horlings H Weigelt B Peterse H Bartelink H van de Vijver MJ Gene expression profiling and histopathological characterization of triple-negative/basal-like breast carcinomas Breast Cancer Res 2007 9 R65 10.1186/bcr1771 17910759 PMC2242660

[B10] McShane LM Altman DG Sauerbrei W Taube SE Gion M Clark GM Statistics Subcommittee of the NCI-EORTC Working Group on Cancer Diagnostics Reporting recommendations for tumor marker prognostic studies J Clin Oncol 2005 23 9067 9072 10.1200/JCO.2004.01.0454 16172462

[B11] Simon RM Paik S Hayes DF Use of archived specimens in evaluation of prognostic and predictive biomarkers J Natl Cancer Inst 2009 101 1446 1452 10.1093/jnci/djp335 19815849 PMC2782246

[B12] Dupuy A Simon RM Critical review of published microarray studies for cancer outcome and guidelines on statistical analysis and reporting J Natl Cancer Inst 2007 99 147 157 10.1093/jnci/djk018 17227998

[B13] Gong Y Yan K Lin F Anderson K Sotiriou C Andre F Holmes FA Valero V Booser D Pippen JE Jr Vukelja S Gomez H Mejia J Barajas LJ Hess KR Sneige N Hortobagyi GN Pusztai L Symmans WF Determination of oestrogen-receptor status and ERBB2 status of breast carcinoma: a gene-expression profiling study Lancet Oncol 2007 8 203 211 10.1016/S1470-2045(07)70042-6 17329190

[B14] Karn T Metzler D Ruckhäberle E Hanker L Gätje R Solbach C Ahr A Schmidt M Holtrich U Kaufmann M Rody A Data driven derivation of cutoffs from a pool of 3,030 Affymetrix arrays to stratify distinct clinical types of breast cancer Breast Cancer Res Treat 2010 120 567 579 10.1007/s10549-009-0416-z 19455418

[B15] Karn T Pusztai L Ruckhäberle E Liedtke C Müller V Schmidt M Metzler D Wang J Coombes KR Gätje R Hanker L Solbach C Ahr A Holtrich U Rody A Kaufmann M Melanoma antigen family A identified by the bimodality index defines a subset of triple negative breast cancers as candidates for immune response augmentation Eur J Cancer 2011 [Epub ahead of print] 10.1016/j.ejca.2011.06.025 21741824

[B16] Sabatier R Finetti P Cervera N Lambaudie E Esterni B Mamessier E Tallet A Chabannon C Extra JM Jacquemier J Viens P Birnbaum D Bertucci F A gene expression signature identifies two prognostic subgroups of basal breast cancer Breast Cancer Res Treat 2011 126 407 420 10.1007/s10549-010-0897-9 20490655

[B17] The R Project for Statistical Computing http://www.r-project.org

[B18] Farmer P Bonnefoi H Becette V Tubiana-Hulin M Fumoleau P Larsimont D Macgrogan G Bergh J Cameron D Goldstein D Duss S Nicoulaz AL Brisken C Fiche M Delorenzi M Iggo R Identification of molecular apocrine breast tumours by microarray analysis Oncogene 2005 24 4660 4671 10.1038/sj.onc.1208561 15897907

[B19] Doane AS Danso M Lal P Donaton M Zhang L Hudis C Gerald WL An estrogen receptor-negative breast cancer subset characterized by a hormonally regulated transcriptional program and response to androgen Oncogene 2006 25 3994 4008 10.1038/sj.onc.1209415 16491124

[B20] Perou CM Jeffrey SS van de Rijn M Rees CA Eisen MB Ross DT Pergamenschikov A Williams CF Zhu SX Lee JC Lashkari D Shalon D Brown PO Botstein D Distinctive gene expression patterns in human mammary epithelial cells and breast cancers Proc Natl Acad Sci USA 1999 96 9212 9217 10.1073/pnas.96.16.9212 10430922 PMC17759

[B21] Palmer C Diehn M Alizadeh AA Brown PO Cell-type specific gene expression profiles of leukocytes in human peripheral blood BMC Genomics 2006 7 115 10.1186/1471-2164-7-115 16704732 PMC1479811

[B22] Rody A Holtrich U Pusztai L Liedtke C Gaetje R Ruckhaeberle E Solbach C Hanker L Ahr A Metzler D Engels K Karn T Kaufmann M T-cell metagene predicts a favorable prognosis in estrogen receptor-negative and HER2-positive breast cancers Breast Cancer Res 2009 11 R15 10.1186/bcr2234 19272155 PMC2688939

[B23] Desmedt C Haibe-Kains B Wirapati P Buyse M Larsimont D Bontempi G Delorenzi M Piccart M Sotiriou C Biological processes associated with breast cancer clinical outcome depend on the molecular subtypes Clin Cancer Res 2008 14 5158 5165 10.1158/1078-0432.CCR-07-4756 18698033

[B24] Schmidt M Böhm D von Törne C Steiner E Puhl A Pilch H Lehr HA Hengstler JG Kölbl H Gehrmann M The humoral immune system has a key prognostic impact in node-negative breast cancer Cancer Res 2008 68 5405 5413 10.1158/0008-5472.CAN-07-5206 18593943

[B25] Alexe G Dalgin GS Scanfeld D Tamayo P Mesirov JP DeLisi C Harris L Barnard N Martel M Levine AJ Ganesan S Bhanot G High expression of lymphocyte-associated genes in node-negative HER2+ breast cancers correlates with lower recurrence rates Cancer Res 2007 67 10669 10676 10.1158/0008-5472.CAN-07-0539 18006808

[B26] Farmer P Bonnefoi H Anderle P Cameron D Wirapati P Becette V André S Piccart M Campone M Brain E Macgrogan G Petit T Jassem J Bibeau F Blot E Bogaerts J Aguet M Bergh J Iggo R Delorenzi M A stroma-related gene signature predicts resistance to neoadjuvant chemotherapy in breast cancer Nat Med 2009 15 68 74 10.1038/nm.1908 19122658

[B27] Bianchini G Qi Y Alvarez RH Iwamoto T Coutant C Ibrahim NK Valero V Cristofanilli M Green MC Radvanyi L Hatzis C Hortobagyi GN Andre F Gianni L Symmans WF Pusztai L Molecular anatomy of breast cancer stroma and its prognostic value in estrogen receptor-positive and -negative cancers J Clin Oncol 2010 28 4316 4323 10.1200/JCO.2009.27.2419 20805453

[B28] Hennessy BT Gonzalez-Angulo AM Stemke-Hale K Gilcrease MZ Krishnamurthy S Lee JS Fridlyand J Sahin A Agarwal R Joy C Liu W Stivers D Baggerly K Carey M Lluch A Monteagudo C He X Weigman V Fan C Palazzo J Hortobagyi GN Nolden LK Wang NJ Valero V Gray JW Perou CM Mills GB Characterization of a naturally occurring breast cancer subset enriched in epithelial-to-mesenchymal transition and stem cell characteristics Cancer Res 2009 69 4116 4124 19435916 10.1158/0008-5472.CAN-08-3441 PMC2737191

[B29] Creighton CJ Li X Landis M Dixon JM Neumeister VM Sjolund A Rimm DL Wong H Rodriguez A Herschkowitz JI Fan C Zhang X He X Pavlick A Gutierrez MC Renshaw L Larionov AA Faratian D Hilsenbeck SG Perou CM Lewis MT Rosen JM Chang JC Residual breast cancers after conventional therapy display mesenchymal as well as tumor-initiating features Proc Natl Acad Sci USA 2009 106 13820 13825 10.1073/pnas.0905718106 19666588 PMC2720409

[B30] Whitney AR Diehn M Popper SJ Alizadeh AA Boldrick JC Relman DA Brown PO Individuality and variation in gene expression patterns in human blood Proc Natl Acad Sci USA 2003 100 1896 1901 10.1073/pnas.252784499 12578971 PMC149930

[B31] Waugh DJ Wilson C The interleukin-8 pathway in cancer Clin Cancer Res 2008 14 6735 6741 10.1158/1078-0432.CCR-07-4843 18980965

[B32] Angelo LS Kurzrock R Vascular endothelial growth factor and its relationship to inflammatory mediators Clin Cancer Res 2007 13 2825 2830 10.1158/1078-0432.CCR-06-2416 17504979

[B33] Bièche I Chavey C Andrieu C Busson M Vacher S Le Corre L Guinebretière JM Burlinchon S Lidereau R Lazennec G CXC chemokines located in the 4q21 region are up-regulated in breast cancer Endocr Relat Cancer 2007 14 1039 1052 10.1677/erc.1.01301 18045955

[B34] Hu Z Fan C Livasy C He X Oh DS Ewend MG Carey LA Subramanian S West R Ikpatt F Olopade OI van de Rijn M Perou CM A compact VEGF signature associated with distant metastases and poor outcomes BMC Med 2009 7 9 10.1186/1741-7015-7-9 19291283 PMC2671523

[B35] Whitfield ML George LK Grant GD Perou CM Common markers of proliferation Nat Rev Cancer 2006 6 99 106 10.1038/nrc1802 16491069

[B36] Paik S Shak S Tang G Kim C Baker J Cronin M Baehner FL Walker MG Watson D Park T Hiller W Fisher ER Wickerham DL Bryant J Wolmark N A multigene assay to predict recurrence of tamoxifen-treated, node-negative breast cancer N Engl J Med 2004 351 2817 2826 10.1056/NEJMoa041588 15591335

[B37] Sotiriou C Wirapati P Loi S Harris A Fox S Smeds J Nordgren H Farmer P Praz V Haibe-Kains B Desmedt C Larsimont D Cardoso F Peterse H Nuyten D Buyse M Van de Vijver MJ Bergh J Piccart M Delorenzi M Gene expression profiling in breast cancer: understanding the molecular basis of histologic grade to improve prognosis J Natl Cancer Inst 2006 98 262 272 10.1093/jnci/djj052 16478745

[B38] Chang HY Nuyten DS Sneddon JB Hastie T Tibshirani R Sørlie T Dai H He YD van't Veer LJ Bartelink H van de Rijn M Brown PO van de Vijver MJ Robustness, scalability, and integration of a wound-response gene expression signature in predicting breast cancer survival Proc Natl Acad Sci USA 2005 102 3738 3743 10.1073/pnas.0409462102 15701700 PMC548329

[B39] Teschendorff AE Miremadi A Pinder SE Ellis IO Caldas C An immune response gene expression module identifies a good prognosis subtype in estrogen receptor negative breast cancer Genome Biol 2007 8 R157 10.1186/gb-2007-8-8-r157 17683518 PMC2374988

[B40] Finak G Bertos N Pepin F Sadekova S Souleimanova M Zhao H Chen H Omeroglu G Meterissian S Omeroglu A Hallett M Park M Stromal gene expression predicts clinical outcome in breast cancer Nat Med 2008 14 518 527 10.1038/nm1764 18438415

[B41] Liedtke C Mazouni C Hess KR André F Tordai A Mejia JA Symmans WF Gonzalez-Angulo AM Hennessy B Green M Cristofanilli M Hortobagyi GN Pusztai L Response to neoadjuvant therapy and long-term survival in patients with triple-negative breast cancer J Clin Oncol 2008 26 1275 1281 10.1200/JCO.2007.14.4147 18250347

[B42] Liedtke C Hatzis C Symmans WF Desmedt C Haibe-Kains B Valero V Kuerer H Hortobagyi GN Piccart-Gebhart M Sotiriou C Pusztai L Genomic grade index is associated with response to chemotherapy in patients with breast cancer J Clin Oncol 2009 27 3185 3191 10.1200/JCO.2008.18.5934 19364972 PMC2716940

[B43] Weigelt B Reis-Filho JS Histological and molecular types of breast cancer: is there a unifying taxonomy? Nat Rev Clin Oncol 2009 6 718 730 10.1038/nrclinonc.2009.166 19942925

[B44] Prat A Perou CM Mammary development meets cancer genomics Nat Med 2009 15 842 844 10.1038/nm0809-842 19661985

[B45] Ruckhäberle E Karn T Engels K Turley H Hanker L Müller V Schmidt M Ahr A Gaetje R Holtrich U Kaufmann M Rody A Prognostic impact of thymidine phosphorylase expression in breast cancer - comparison of microarray and immunohistochemical data Eur J Cancer 2010 46 549 557 10.1016/j.ejca.2009.11.020 20022486

[B46] Hu Z Fan C Oh DS Marron JS He X Qaqish BF Livasy C Carey LA Reynolds E Dressler L Nobel A Parker J Ewend MG Sawyer LR Wu J Liu Y Nanda R Tretiakova M Ruiz Orrico A Dreher D Palazzo JP Perreard L Nelson E Mone M Hansen H Mullins M Quackenbush JF Ellis MJ Olopade OI Bernard PS The molecular portraits of breast tumors are conserved across microarray platforms BMC Genomics 2006 7 96 10.1186/1471-2164-7-96 16643655 PMC1468408

[B47] Kreike B van de Vijver MJ Are triple-negative tumours and basal-like breast cancer synonymous? Authors' response Breast Cancer Res 2007 9 405 10.1186/bcr1832 PMC2246182 18279542

[B48] Lusa L McShane LM Reid JF De Cecco L Ambrogi F Biganzoli E Gariboldi M Pierotti MA Challenges in projecting clustering results across gene expression-profiling datasets J Natl Cancer Inst 2007 99 1715 1723 10.1093/jnci/djm216 18000217

[B49] Weigelt B Mackay A A'hern R Natrajan R Tan DS Dowsett M Ashworth A Reis-Filho JS Breast cancer molecular profiling with single sample predictors: a retrospective analysis Lancet Oncol 2010 11 339 349 10.1016/S1470-2045(10)70008-5 20181526

[B50] Bertucci F Finetti P Cervera N Esterni B Hermitte F Viens P Birnbaum D How basal are triple-negative breast cancers? Int J Cancer 2008 123 236 240 10.1002/ijc.23518 18398844

[B51] Cheang MC Voduc D Bajdik C Leung S McKinney S Chia SK Perou CM Nielsen TO Basal-like breast cancer defined by five biomarkers has superior prognostic value than triple-negative phenotype Clin Cancer Res 2008 14 1368 1376 10.1158/1078-0432.CCR-07-1658 18316557

[B52] Huang E Cheng SH Dressman H Pittman J Tsou MH Horng CF Bild A Iversen ES Liao M Chen CM West M Nevins JR Huang AT Gene expression predictors of breast cancer outcomes Lancet 2003 361 1590 1596 10.1016/S0140-6736(03)13308-9 12747878

[B53] Calabrò A Beissbarth T Kuner R Stojanov M Benner A Asslaber M Ploner F Zatloukal K Samonigg H Poustka A Sültmann H Effects of infiltrating lymphocytes and estrogen receptor on gene expression and prognosis in breast cancer Breast Cancer Res Treat 2009 116 69 77 10.1007/s10549-008-0105-3 18592372

[B54] Denkert C Loibl S Noske A Roller M Müller BM Komor M Budczies J Darb-Esfahani S Kronenwett R Hanusch C von Törne C Weichert W Engels K Solbach C Schrader I Dietel M von Minckwitz G Tumor-associated lymphocytes as an independent predictor of response to neoadjuvant chemotherapy in breast cancer J Clin Oncol 2010 28 105 113 10.1200/JCO.2009.23.7370 19917869

[B55] van de Vijver MJ He YD van't Veer LJ Dai H Hart AA Voskuil DW Schreiber GJ Peterse JL Roberts C Marton MJ Parrish M Atsma D Witteveen A Glas A Delahaye L van der Velde T Bartelink H Rodenhuis S Rutgers ET Friend SH Bernards R A gene-expression signature as a predictor of survival in breast cancer N Engl J Med 2002 347 1999 2009 10.1056/NEJMoa021967 12490681

[B56] Fan C Oh DS Wessels L Weigelt B Nuyten DS Nobel AB van't Veer LJ Perou CM Concordance among gene-expression-based predictors for breast cancer N Engl J Med 2006 355 560 569 10.1056/NEJMoa052933 16899776

[B57] Wirapati P Sotiriou C Kunkel S Farmer P Pradervand S Haibe-Kains B Desmedt C Ignatiadis M Sengstag T Schütz F Goldstein DR Piccart M Delorenzi M Meta-analysis of gene expression profiles in breast cancer: toward a unified understanding of breast cancer subtyping and prognosis signatures Breast Cancer Res 2008 10 R65 10.1186/bcr2124 18662380 PMC2575538

[B58] Reyal F van Vliet MH Armstrong NJ Horlings HM de Visser KE Kok M Teschendorff AE Mook S van 't Veer L Caldas C Salmon RJ van de Vijver MJ Wessels LF A comprehensive analysis of prognostic signatures reveals the high predictive capacity of the proliferation, immune response and RNA splicing modules in breast cancer Breast Cancer Res 2008 10 R93 10.1186/bcr2192 19014521 PMC2656909

[B59] Wang Y Klijn JG Zhang Y Sieuwerts AM Look MP Yang F Talantov D Timmermans M Meijer-van Gelder ME Yu J Jatkoe T Berns EM Atkins D Foekens JA Gene-expression profiles to predict distant metastasis of lymph-node-negative primary breast cancer Lancet 2005 365 671 679 15721472 10.1016/S0140-6736(05)17947-1

[B60] Eggermont AM Testori A Maio M Robert C Anti-CTLA-4 antibody adjuvant therapy in melanoma Semin Oncol 2010 37 455 459 10.1053/j.seminoncol.2010.09.009 21074060

[B61] Calabrò L Danielli R Sigalotti L Maio M Clinical studies with anti-CTLA-4 antibodies in non-melanoma indications Semin Oncol 2010 37 460 467 10.1053/j.seminoncol.2010.09.006 21074061

[B62] Liu S Wicha MS Targeting breast cancer stem cells J Clin Oncol 2010 28 4006 4012 10.1200/JCO.2009.27.5388 20498387 PMC4872314

[B63] Ginestier C Liu S Diebel ME Korkaya H Luo M Brown M Wicinski J Cabaud O Charafe-Jauffret E Birnbaum D Guan JL Dontu G Wicha MS CXCR1 blockade selectively targets human breast cancer stem cells *in vitro *and in xenografts J Clin Invest 2010 120 485 497 10.1172/JCI39397 20051626 PMC2810075

[B64] Grier DG Thompson A Kwasniewska A McGonigle GJ Halliday HL Lappin TR The pathophysiology of HOX genes and their role in cancer J Pathol 2005 205 154 171 10.1002/path.1710 15643670

[B65] Stein GS Stein JL van Wijnen AJ Lian JB Histone gene transcription: a model for responsiveness to an integrated series of regulatory signals mediating cell cycle control and proliferation/differentiation interrelationships J Cell Biochem 1994 54 393 404 10.1002/jcb.240540406 8014188

