# Supplementary material for: A clinically relevant gene signature in triple negative and basal-like breast cancer
Source: Breast Cancer Res. 2011 Oct 6;13(5):R97. doi: 10.1186/bcr3035 (PMC3262210; doi:10.1186/bcr3035)
Supplement: Additional file 1 — Supplementary Figures S1 to S15. An Adobe file containing 15 supplementary figures (S1 to S15). [file bcr3035-S1.PDF]

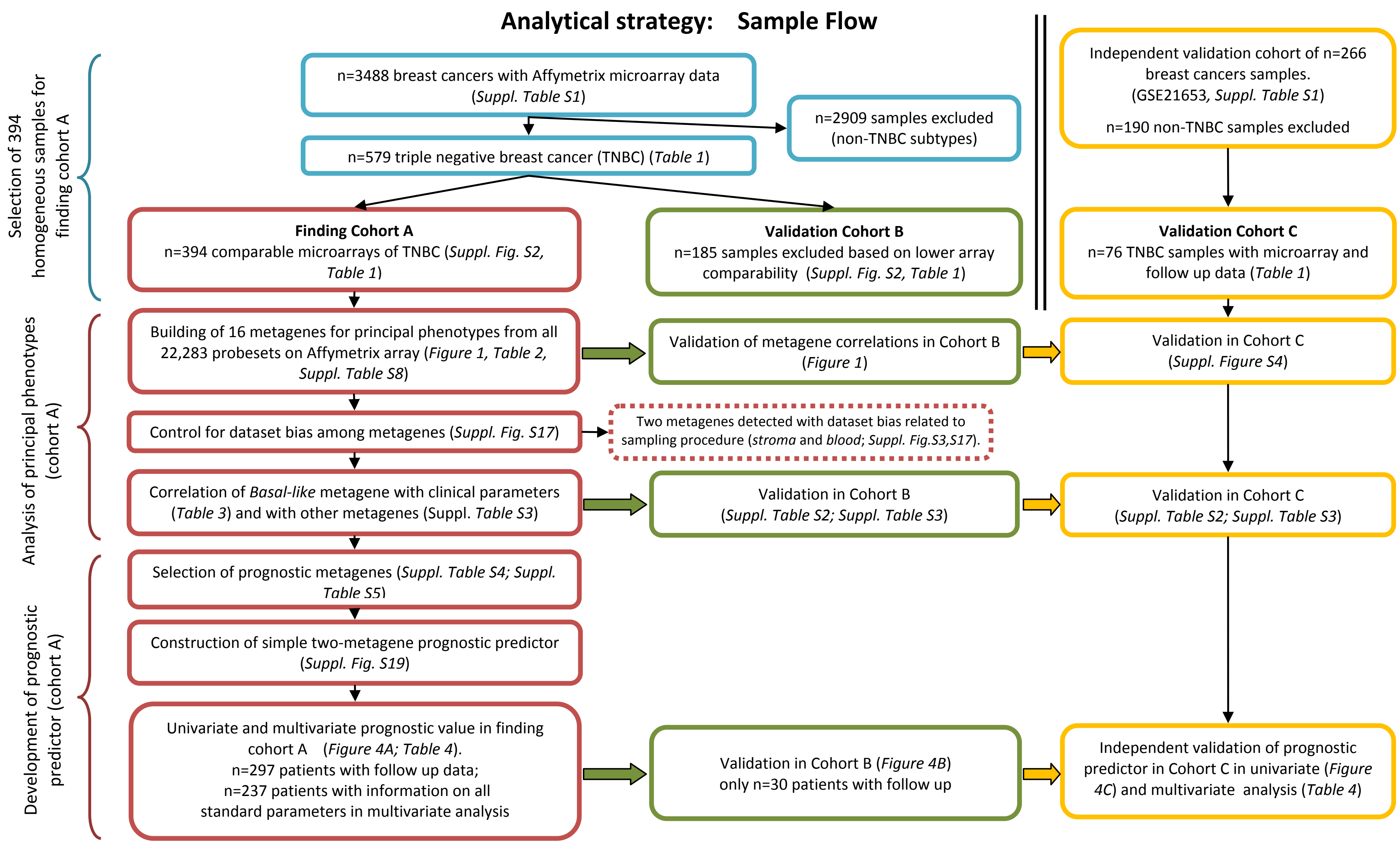

Supplementary Figure S1: Sample flow in analysis according to REMARK criteria (McShane et al. J Clin Oncol. 2005;23:9067).

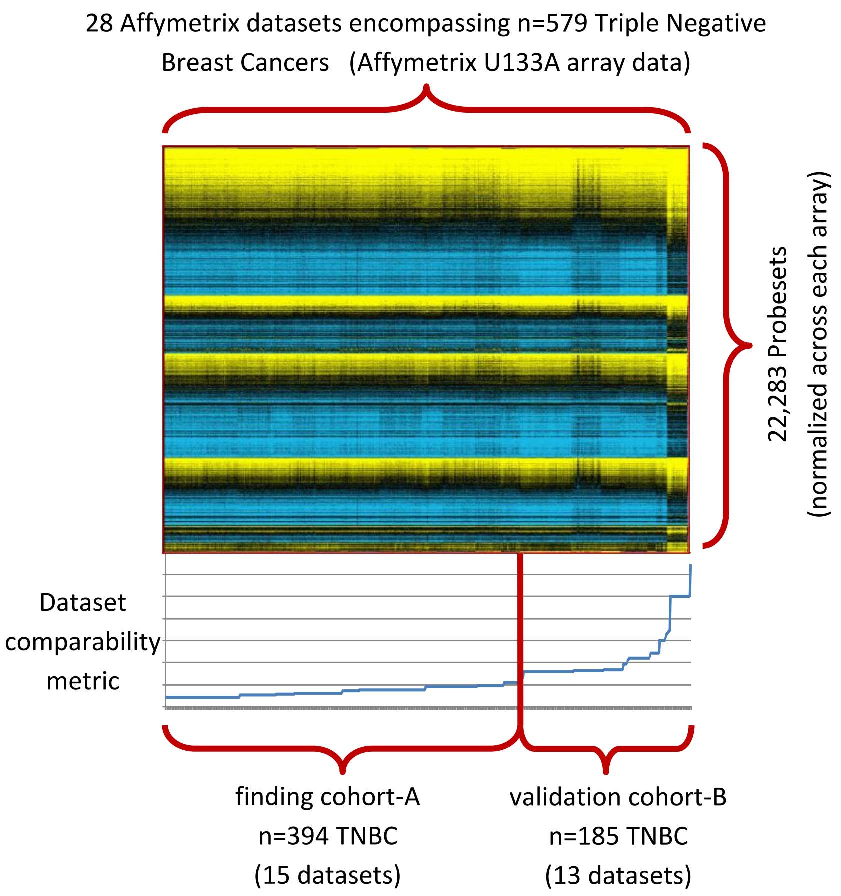

Supplementary Figure S2: Selection of the TNBC finding cohort from multiple datasets based on dataset comparibility

Triple negative breast cancers (TNBC, n=579) from 28 datasets were sorted by dataset according to a dataset comparability metric (horizontally). Shown are the full array data of normalized Affymetrix U133A microarrays. The 15 most comparable datasets encompassing n=394 TNBC samples were subsequently used as a finding cohort-A and the remaining 13 datasets (n=185 TNBC samples) withhold as validation cohort-B.

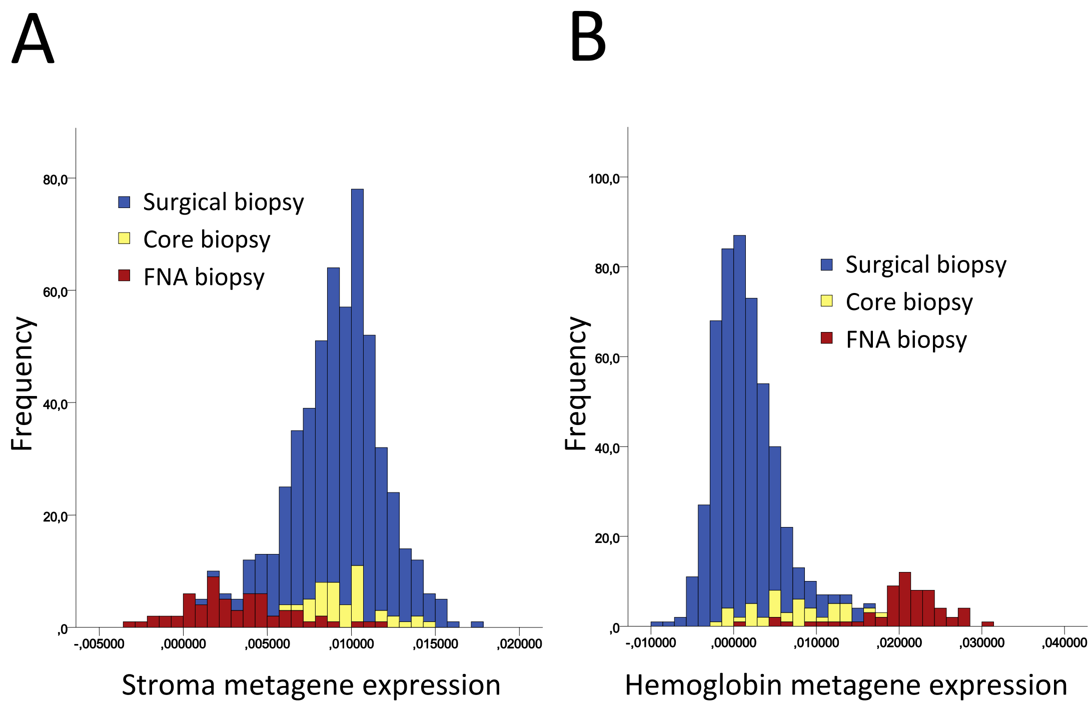

Supplementary Figure S3: Influence of biopsy method on expression of stroma and hemoglobin metagenes

The distribution of the expression of the stroma (A) and hemoglobin (B) metagenes among the n=579 TNBC samples of cohort-A and -B is shown. Different colours are used according to the applied biopsy method. Samples obtained by fine needle aspiration (FNA) are characterized by low stroma metagene expression and high hemoglobin metagene expression. Such samples are known to contain relative high amounts of blood and low amounts of stromal tissue. (Identical results were obtained when the analysis was performed using cohorts-A and –B separately).

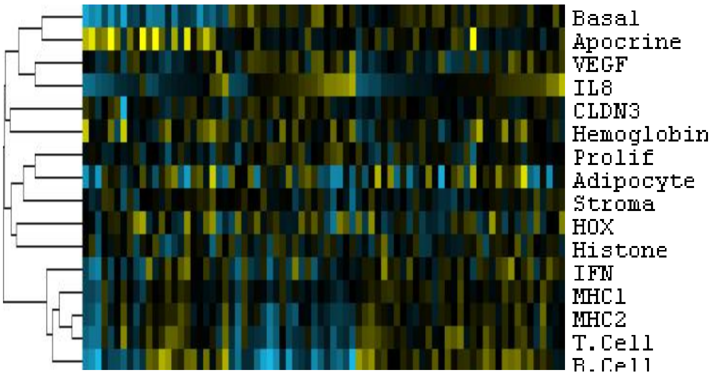

Supplementary Figure S4: Correlation of the expression of the 16 metagenes among 76 TNBC samples from validation cohort-C

A heatmap of expression values of the 16 metagenes is shown for the 76 TNBC samples from validation cohort-C. The dendrogram at the left presents the results from hierarchical clustering of the metagenes. Samples were sorted as in Figure 1 according to (1.) Basal-like phenotype, (2.) low vs. high B-Cell metagene, and (3.) the expression value of the IL-8 metagene.

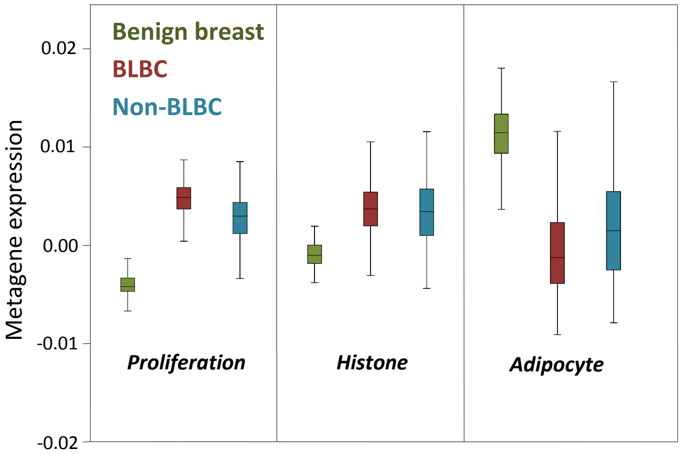

Supplementary Figure S5: Relationship of non-BLBC samples to benign breast tissue

Box plots comparing the expression of *Proliferation*, *Histone*, and *Adipocyte* metagenes between samples of benign breast tissue, and tumors of the BLBC and Non-BLBC subtype of TNBC. Benign breast tissue is characterized by low expression of both *Proliferation* and *Histone* metagenes but high expression of the *Adipocyte* metagene. In contrast tumor samples of both BLBC and Non-BLBC type are similar to each other but differ significantly from benign breast in the expression of all three metagenes.

(Affymetrix expression data from n=140 normal breast biopsies were obtained from GSE10780: Chen DT, Nasir A, Culhane A, Venkataramu C, Fulp W, Rubio R, Wang T, Agrawal D, McCarthy SM, Gruidl M *et al*: Proliferative genes dominate malignancy-risk gene signature in histologically-normal breast tissue. *Breast Cancer Res Treat* 2009.)

A

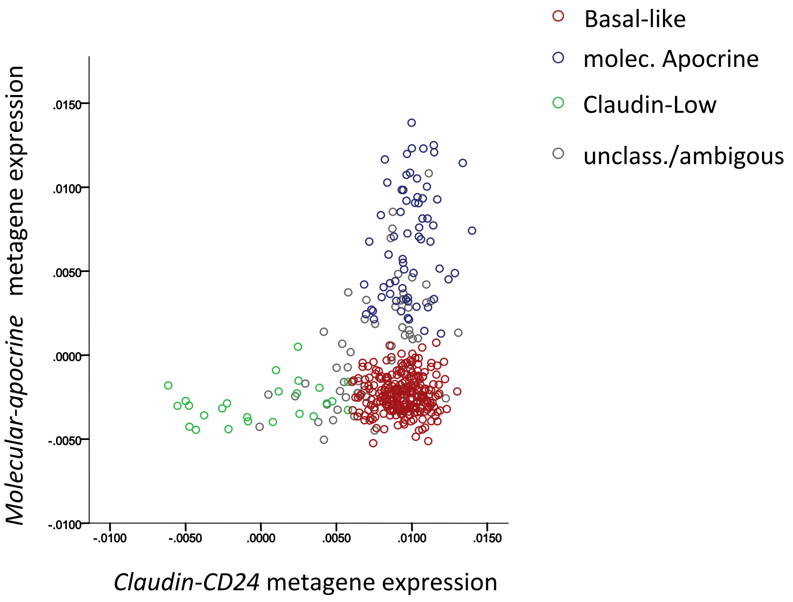

B

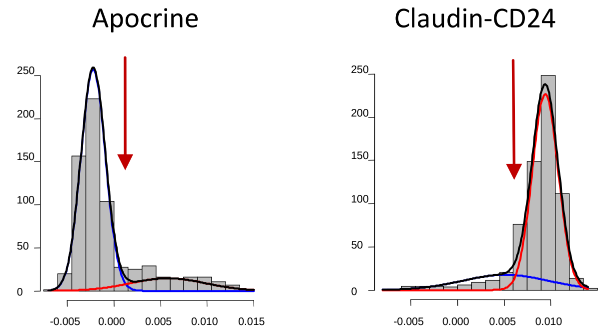

Supplementary Figure S6: Mutual relationship between the *Apocrine*, *Claudin-CD24*, and *Basal-like* metagenes in TNBC

A) Scatter plot of the expression of the *Apocrine* and the *Claudin-CD24* metagenes among the 394 TNBC samples from the finding cohort-A. Samples are coloured according to their classification as either Basal-like (red, 63.2%), Molecular-Apocrine (blue, 16.5%), or Claudin-Low (green, 6.3%). Classifications were performed based on cutoffs derived from the distribution of the respective metagenes (given in B). Samples assigned to two different subtypes based on these cutoffs were classified as "unclassified/ambigous" (grey, 14.0%) in this plot.

B) Histogramms of the expression of the *Apocrine* and *Claudin-CD24* metagenes used for derivation of the respective cutoff values (arrows) through fitting a mixture of two normal distributions.

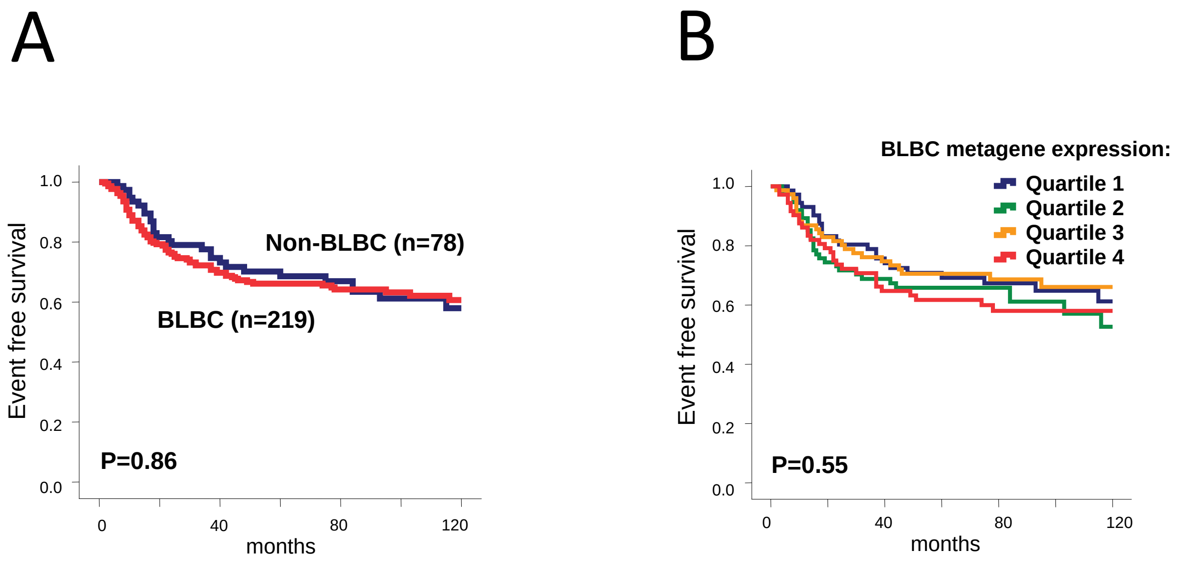

Supplementary Figure S7: **Analysis of the prognostic value of the Basal-like metagene among TNBC of cohort A**

A) Kaplan Meier analysis of event free survival of TNBC patients from cohort-A stratified as BLBC (n=219) or Non-BLBC (n=78) based on the cutoff (0.0014) derived from the distribution of the *Basal-like* metagene.

B) The TNBC cohort-A was stratified into quartiles according to the expression of the *Basal-like* metagene and Kaplan Meier analysis of event free survival of the corresponing groups performed. However no trend for a prognostic value was detected among the 297 patients with follow up information. Similar results were obtained in the validation cohorts (not shown).

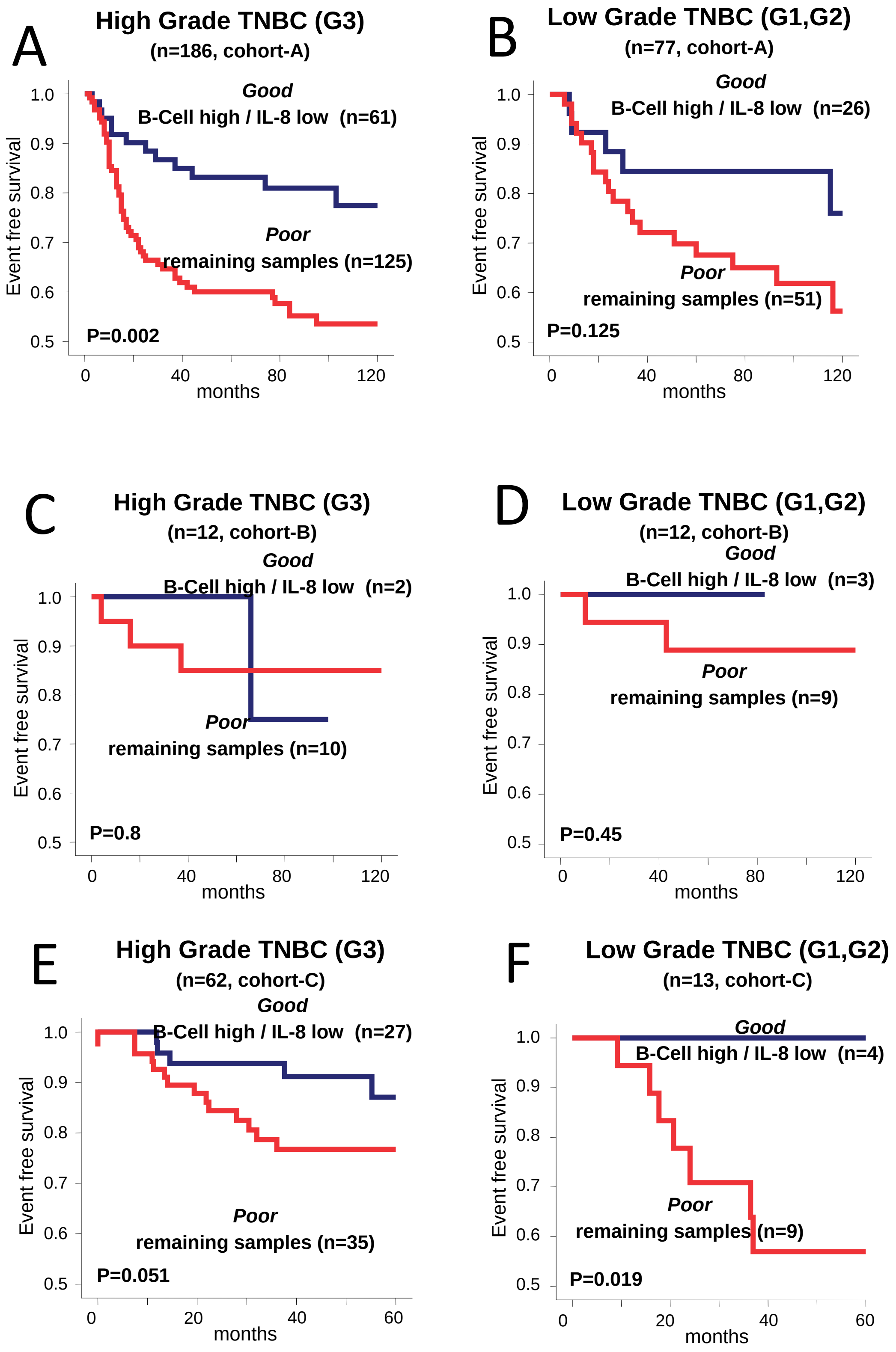

Suppl. Figure S8: Prognostic value of the the combined B-Cell/IL-8 metagenes among TNBC in subgroups according to pathohistological grading in the three individual cohorts

Kaplan Meier analysis of event free survival of TNBC patients with follow up from the finding cohort-A (in panels A and B), the validation cohort-B (in panels C and D), and the validation cohort-C (in panels E and F). The cohorts were further stratified according to pathohistological grading with either high grade (G3) tumors in panels A, C, and E or low grade (G1,G2) tumors in panels B, D, and F. Samples were stratified according to prognostic predictor of the combined B-Cell/IL-8 metagenes. *"Good"* refers to samples with both high B-Cell and low IL-8 metagene expression whereas all other samples are referred as "*Poor*".

A

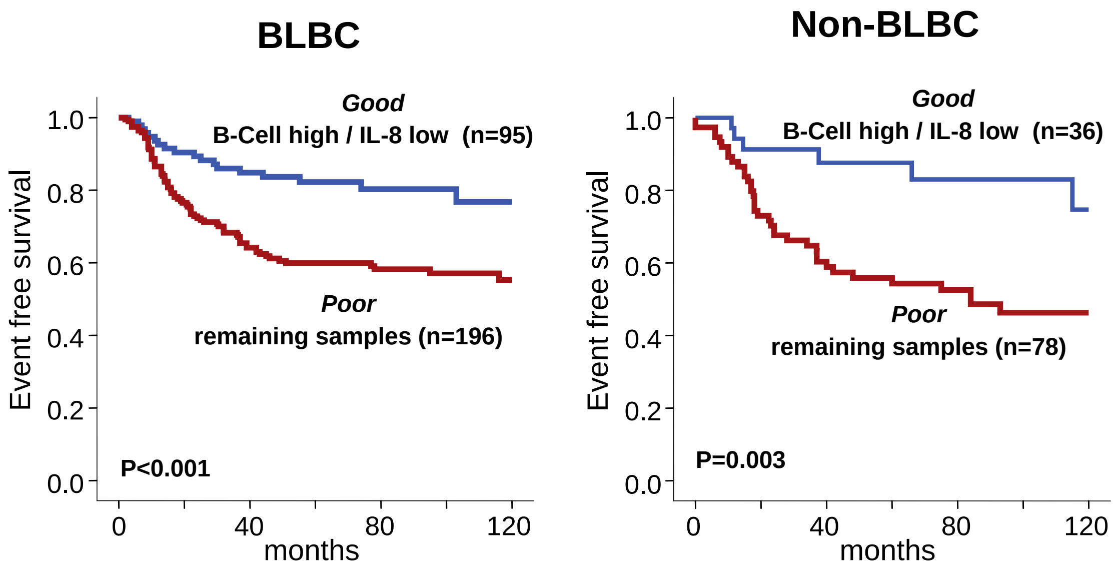

B

		BLBC			Non-BLBC		
**Cohort:**	**n (total)**	**P-Value**	**n Good**	**n Poor**	**P-Value**	**n Good**	**n Poor**
cohort-A	297	**0.004**	71	148	**0.001**	24	54
cohort-B	30	0.640	3	17	0.159	2	8
cohort-C	75	**0.011**	21	31	0.110	10	13
cohort A+B	327	**0.006**	74	165	**0.004**	26	62
all combined	402	**<0.001**	95	196	**0.003**	36	78

Supplementary Figure S9: Prognostic value of the combined B-Cell/IL-8 metagenes within the groups of *Basal-like* and *Non-basal-like* TNBC.

A) Kaplan Meier analysis of event free survival of 402 TNBC patients with follow up from all three cohorts (cohort-A, -B, and –C). Samples were stratified according to prognostic predictor of the combined B-Cell/IL-8 metagenes. *"Good"* refers to 131 samples with both high B-Cell and low IL-8 metagene expression whereas all other samples (n=274) are referred as "*Poor*". Two separate Kaplan Meier graphs are presented for patients with tumors either classified as Basal-like breast cancer (BLBC, left) or Non-BLBC (right). The B-Cell/IL8 metagene ratio demonstrated a prognostic value in both subgroups (HR 0.41, 95%CI 0.25-0.69, P<0.001; and HR 0.30, 95%CI 0.13-0.70, P=0.003; respectively).

B) Results of the respective Kaplan Meier analyses of the individual cohorts are given using the same stratification strategy as in (*A*).

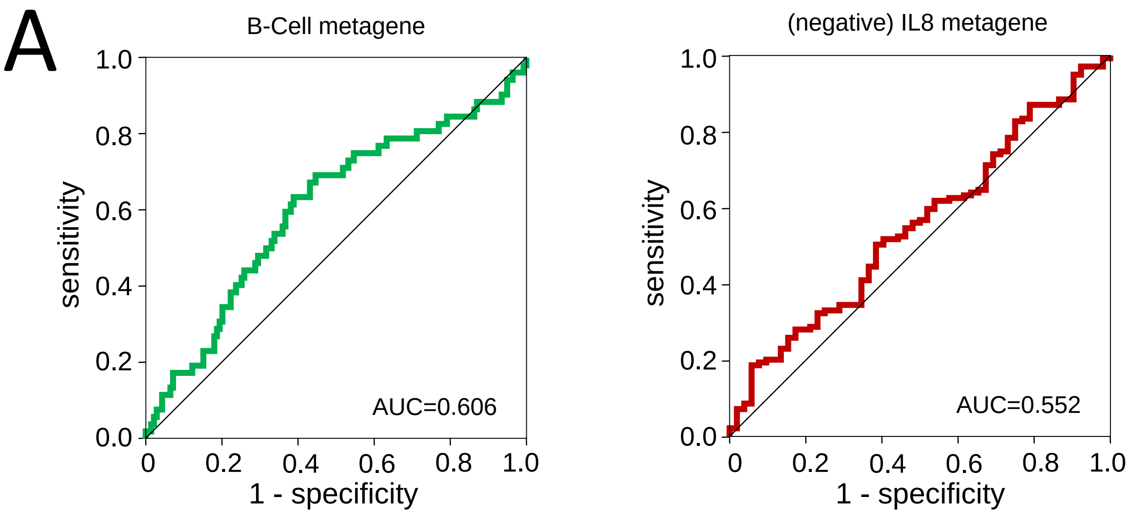

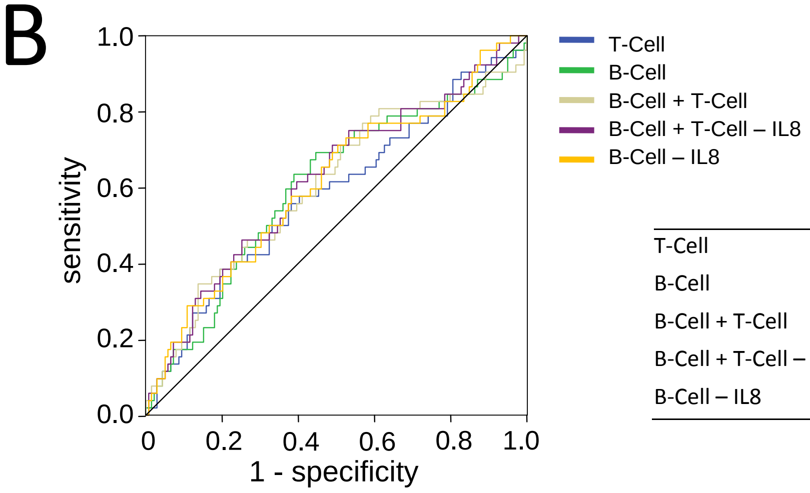

	AUC	SE	P-Value
T-Cell	0.584	0.048	0.076
B-Cell	0.606	0.048	0.025
B-Cell + T-Cell	0.607	0.048	0.023
B-Cell + T-Cell – IL8	0.619	0.047	0.012
B-Cell – IL8	0.612	0.047	0.018

Supplementary Figure S10: Analysis of the predictive value of the combined B-Cell/IL-8 metagenes for response to neoadjuvant chemotherapy in TNBC.

A) Neoadjuvant treated TNBC samples with information on pathological complete response (pCR) and available Affymetrix expression data were assembled from 7 datasets (MDA133, GSE16716, GSE18728, GSE19697, GSE20194, GSE20271, Frankfurt-3). Only pretherapeutic biopsies that were not microdissected were included (n=191 nonredundant samples) of which 52 (27%) experienced a pCR. Two separate ROC curves for prediction of pCR by the B-Cell metagene and no-pCR by the IL8 metagene are shown with an area under the curve (AUC) of 0.606 and 0.552, respectively.

B) ROC curves of the T-Cell and B-Cell metagenes as well as combinations of both metagenes and the IL8 metagene are shown for the prediction of pCR as in (*A*). The respective AUC values are given in the table on the right. Combinations of B-Cell and IL8 metagenes or addition of the T-Cell metagene slightly increase the AUC to a maximum of 0.619.

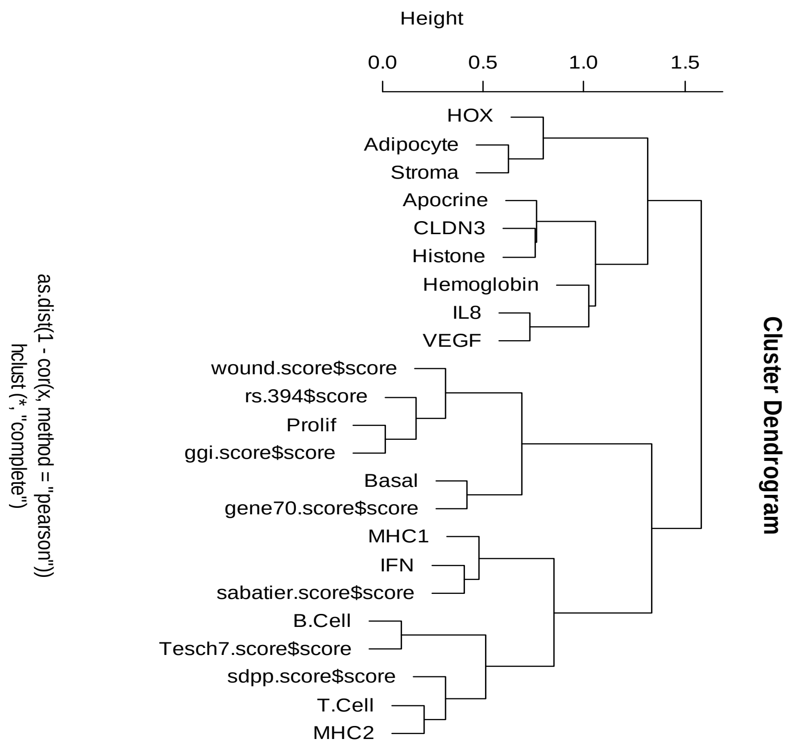

wound.score$score	=	"Wound response signature"
rs.394$score	=	"Recurrence Score"
ggi.score$score	=	"Genomic Grading Index"
gene70.score$score	=	"Amsterdam 70 gene signature"
sabatier.score$score	=	"Sabatier - 368-gene-medullary-BC-signature"
Tesch7.score$score	=	"Teschendorff - 7-gene immune response (IR) signature"
sdpp.score$score	=	"Finak – Stroma Derived Prognostic Predictor"
CLDN3	=	Claudin-CD24 metagene
Prolif	=	Proliferation metagene

Supplementary Figure S11: Relationship of previously published gene signatures to the metagenes detected within TNBC.

The correlation of several published gene signatures to the metagenes discovered within the pure TNBC cohort was analyzed by hierarchical clustering using gene expression data from the homogenous cohort-A of 394 TNBC. "*Recurrence Score*" (Paik et al. 2004 N Engl J Med. 351:2817), "*Genomic Grading Index*" (GGI; Sotiriou et al. 2006 J Natl Cancer Inst. 98:262), and the "*Wound response signature*" (Chang et al. 2005 Proc Natl Acad Sci USA 102:3738) display high correlation to the proliferation metagene. The "*7-gene immune response (IR) signature*", the "*Stroma derived prognostic predictor*" (SDPP; Finak et al. 2008 Nat Med. 14:518), and the "*368 gene medullary breast cancer signature*" (Sabatier et al. 2011 Breast Cancer Res Treat. 126:407) were all highly correlated to immune cell metagenes. No correlations of the signatures to the IL8 metagene were observed.

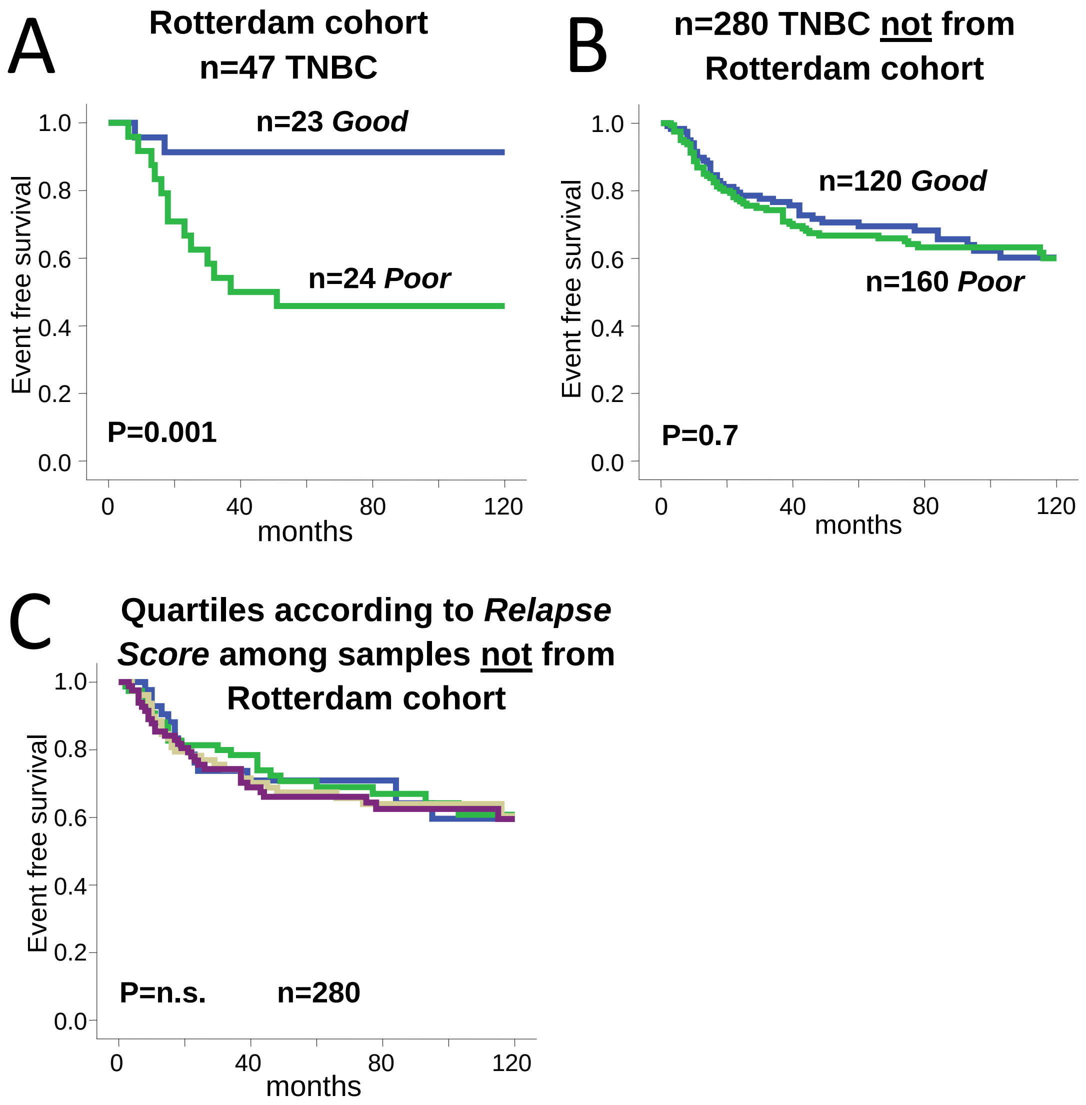

Supplementary Figure S12: Prognostic value of the *Rotterdam Signature* in TNBC

A) The n=47 TNBC cases from the Rotterdam finding cohort were stratified according to the *Relapse Score* derived from the 16 genes of the *Rotterdam signature* as requested for ER negative samples (Wang et al. 2005). Kaplan Meier analysis of event free survival is shown for the samples with a *Good* and *Poor* Relapse Score, respectively.

B) For validation 280 TNBC cases <u>not</u> from the Rotterdam cohort with follow up information were stratified as either *Good* or *Poor Prognosis* according to the Relapse Score as in (A). As shown no significant prognostic value was detected both in the complete cohort as well as in the individual datasets (not shown).

C) The same analysis as in (B) was performed using quartiles according to the *Relapse Score* of the *Rotterdam* signature.

Analytical strategy: Development of prognostic predictors

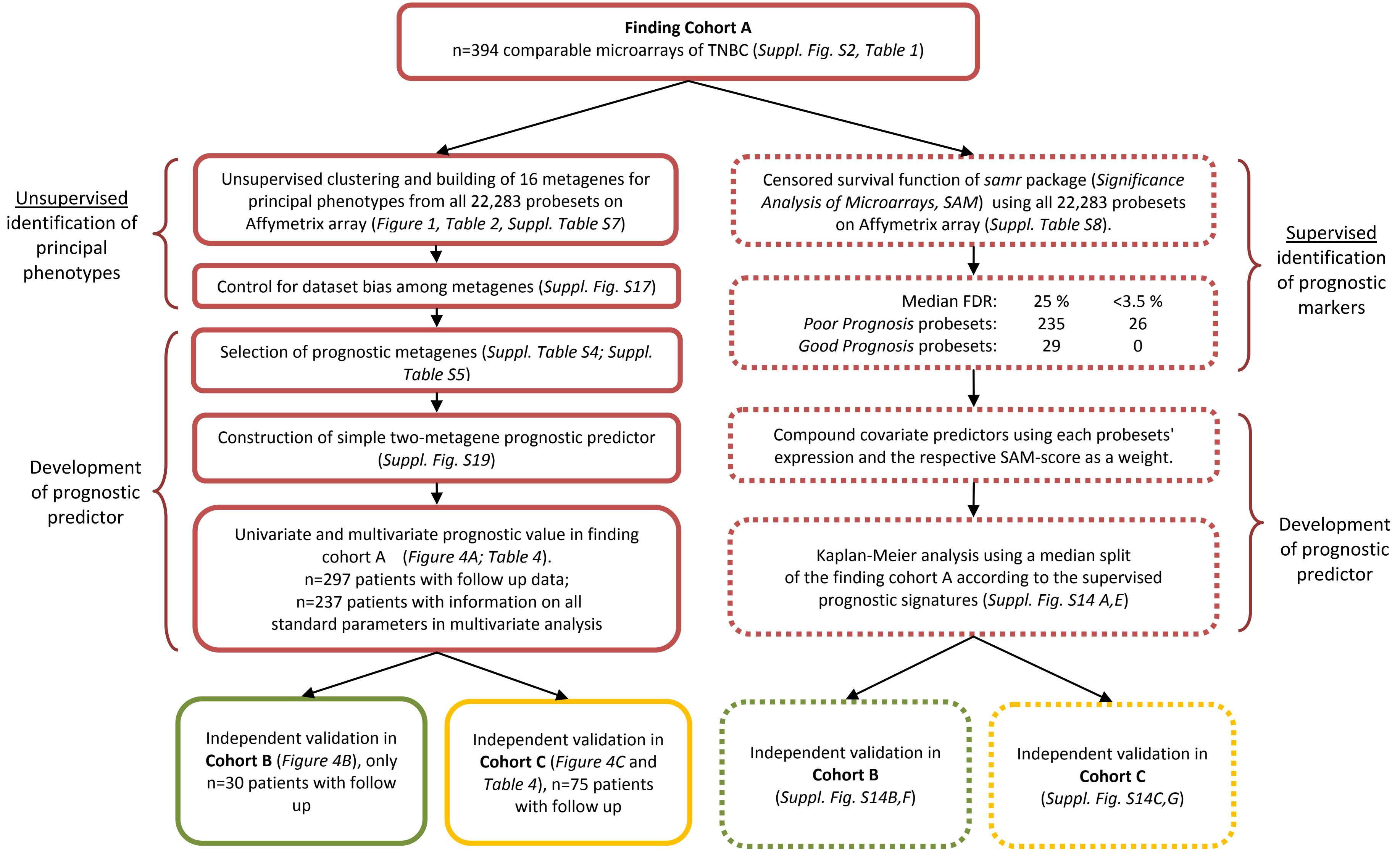

Supplementary Figure S13: Development and validation of prognostic predictors according to REMARK criteria (McShane et al. J Clin Oncol. 2005;23:9067).

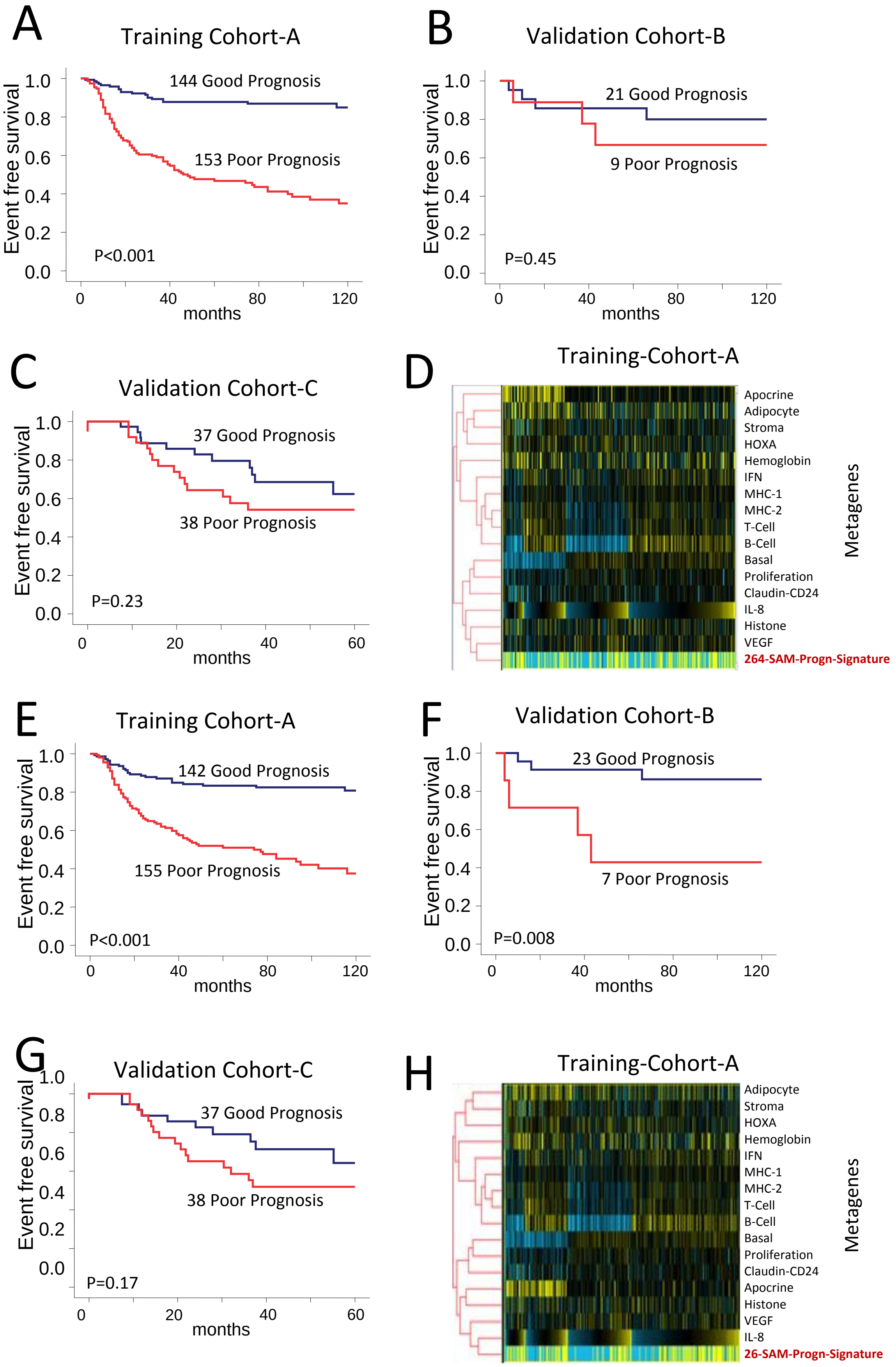

Supplementary Figure S14 ***– (continued) –***

Supplementary Figure S14: Supervised Prognostic Classification using Significance Analysis of Microarrays (SAM) in TNBC

The Cox score option for censored survival data of *Significance Analysis of Microarrays* (SAM; Tusher et al. Proc Natl Acad Sci U S A. 2001 ;98:5116) was applied to the finding cohort-A using the R-package *samr* and including all probesets on the Affymetrix U133A array.

A) - D): 235 probsets associated with poor prognosis and 29 probesets associated with good prognosis were identified with a median false discovery rate of 25% (Suppl. Table S8). A supervised prognostic signature was derived as a compound covariate predictor using each probesets' expression value and the respective SAM-Score as a weight. Kaplan-Meier curves using a median split of the cohorts according to the supervised prognostic signature demonstrate a significant difference in prognosis in the training cohort (A). In contrast, only a trend was found among the validation cohorts (B and C). Panel D displays a cluster analysis of the metagenes from Figure 1 and the SAM-derived prognostic signature as continous variables in the finding cohort-A. The SAM-derived prognostic signature clustered together with IL-8, Histone and VEGF metagenes in one cluster (similar results were obtained using the two validation cohorts-B and –C).

E) - H): When the stringency of the SAM-analysis was increased (δ=0.5) only 26 probesets associated with poor prognosis were identified with a median false discovery rate of 3.5% (no probesets associated with good prognosis were identified using this higher stringency; Suppl. Table S8). Panels (E), (F), and (G) show the corresponding analyses to panels (A), (B), and (C), respectively, using the prognostic signature derived from these 26 probesets. A significant difference in prognosis was found for validation cohort B (panel F) but only a trend for validation cohort C (panel G). The cluster analysis in panel (H) demonstrates that this 26-probeset-signature displayed the highest correlation to the IL-8 metagene (the same result was obtained using validation cohorts–B and –C; not shown).

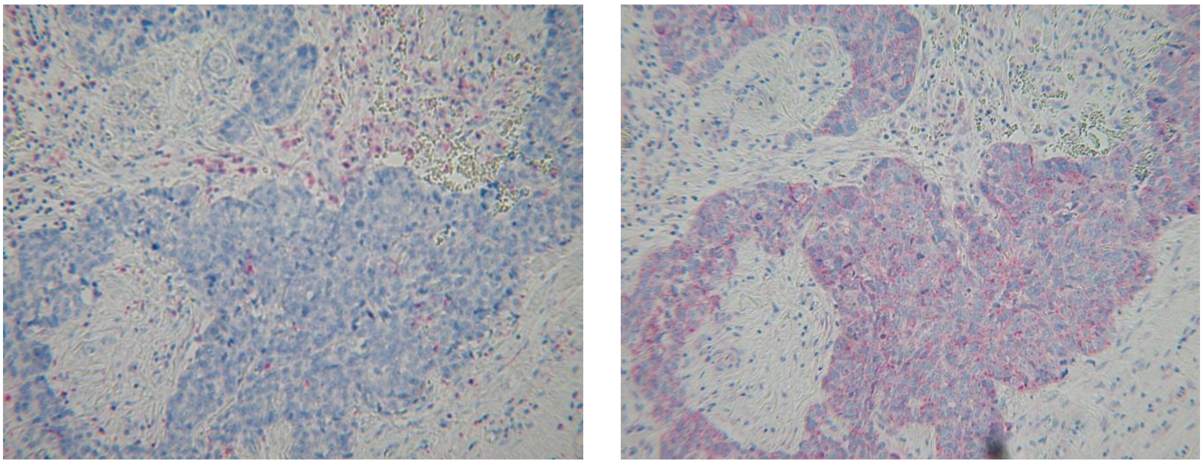

Supplementary Figure S15: Immunohistochemical analyses of the cellular source of IL8 expression in TNBC through comparison with macrophage marker CD68

A) Detection of macrophages by a CD68 antibody (red staining) in a triple negative breast cancer from the Frankfurt cohort with high expression of the IL-8 metagene.
B) An adjacent section of the same tumor as in (A) is stained with an IL-8 antibody. The main source of IL-8 expression (red staining) results from carcinoma cells and not macrophages.
