# Supplementary material for: A clinically relevant gene signature in triple negative and basal-like breast cancer
Source: Breast Cancer Res. 2011 Oct 6;13(5):R97. doi: 10.1186/bcr3035 (PMC3262210; doi:10.1186/bcr3035)
Supplement: Additional file 2 — Supplementary Tables S1 to S7. An Adobe file containing seven supplementary tables (S1 to S7). [file bcr3035-S2.PDF]

Supplementary Table S1: Summary of Affymetrix microarray datasets used in this study

*Dataset **	*Data Source*	*% of samples (complete datasets)*								*No. of samples*							
		Age ≤50	***Tumor size ≤2cm***	***LNN***	***G3***	***No systemic treatment***	***endocrine treatment***	***chemotherapy***	***Relapse***	***total***	***TNBC***	***Finding cohort-A***	***Validation cohort-B***	***Dataset Comparability metric C***	***DMFS data only***	***surgical biopsies***	***Reference***
Rotterdam-EMC344	*GSE2034, GSE5327*	*55*	*35*	*100*	*72*	*100*	*0*	*0*	*28*	*344*	*82*	*82*	*0*	*0.0085*	*x*	*+*	*i,ii,iii*
TransBIG	*GSE7390*	*80*	*18*	*100*	*75*	*100*	*0*	*0*	*43*	*198*	*40*	*40*	*0*	*0.0110*		*+*	*iv*
Mainz	*GSE11121*	*33*	*33*	*100*	*71*	*100*	*0*	*0*	*38*	*200*	*21*	*21*	*0*	*0.0115*	*x*	*+*	*v*
Stockholm	*GSE1456*				*74*	*45*	*55*	*0*	*40*	*159*	*25*	*25*	*0*	*0.0119*		*+*	*vi*
Uppsala	*GSE3494 (n=251), GSE6232 (n=5), GSE4922 (n=1), GSE2990 (n=1)*	*37*	*41*	*68*	*59*	*86*	*14*	*0*	*26*	*258*	*27*	*27*	*0*	*0.0124*		*+*	*vii,viii*
Frankfurt-2	*GSE31519*	*50*	*0*	*39*	*56*				*33*	*67*	*19*	*19*	*0*	*0.0146*			*ix*
Frankfurt	*GSE31519*	*33*	*42*	*83*	*83*	*0*	*0*	*100*	*33*	*119*	*24*	*24*	*0*	*0.0155*		*+*	*x*
New York	*GSE2603*	*40*	*11*	*49*		*0*	*4*	*96*	*48*	*99*	*35*	*35*	*0*	*0.0155*	*x*	*+*	*xi*
Oxford-Untreated	*GSE2990 (n=61), GSE6532 (n=8)*	*54*	*46*	*100*	*60*	*100*	*0*	*0*	*45*	*69*	*13*	*13*	*0*	*0.0157*		*+*	*xii*
Hamburg-2	*GSE31519*	*0*	*0*	*71*	*40*	*0*	*100*	*0*	*20*	*77*	*7*	*7*	*0*	*0.0180*	*x*	*+*	*xiii*
Hamburg-1	*GSE31519*	*53*	*33*	*80*	*86*	*0*	*0*	*100*	*47*	*77*	*15*	*15*	*0*	*0.0182*		*+*	*xiv*
Signapore	*GSE5364*									*183*	*36*	*36*	*0*	*0.0186*		*+*	*xv*
MDA133	*mdanderson.org*	*38*	*21*	*24*	*83*					*133*	*29*	*29*	*0*	*0.0189*		*-*	*xvi*
Boston	*GSE3744*				*100*					*40*	*16*	*16*	*0*	*0.0224*		*+*	*xvii*
Tampa	*GSE10780*									*39*	*5*	*5*	*0*	*0.0262*		*+*	*xviii*
Rotterdam-EMC204	*GSE12276*	*55*	*22*		*71*	*63*	*25*	*13*		*204*	*56*	*0*	*56*	*0.0320*		*+*	*xix*
MDA100	*GSE16716*	*48*	*3*	*18*	*73*	*0*	*0*	*100*		*100*	*33*	*0*	*33*	*0.0327*		*-*	*xx*
EORTC	*GSE1561*		*0*	*43*	*68*					*49*	*21*	*0*	*21*	*0.0338*		*-*	*xxi*
Genentech	*GSE12763*									*30*	*5*	*0*	*5*	*0.0389*		*+*	*xxii*
Frankfurt-3	*GSE31519*	*0*	*50*	*50*	*50*	*0*	*100*	*0*	*50*	*52*	*2*	*0*	*2*	*0.0427*		*+*	*xxiii*
San Francisco	*E-TABM-158*	*52*	*35*	*52*	*45*	*17*	*13*	*70*	*22*	*118*	*23*	*0*	*23*	*0.0441*	*x*	*+*	*xxiv*
Paris	*GSE13787*				*100*					*23*	*10*	*0*	*10*	*0.0487*		*+*	*xxv*
Berlin	*GSE6596*	*29*	*29*		*71*					*24*	*7*	*0*	*7*	*0.0598*		*+*	*xxvi*
Veridex-Tam	*GSE12093*			*100*		*0*	*100*	*0*	*0*	*136*	*1*	*0*	*1*	*0.0650*	*x*	*+*	*xxvii*
London-2	*GSE9195*	*0*	*100*	*100*	*100*	*0*	*100*	*0*	*0*	*77*	*2*	*0*	*2*	*0.0663*		*+*	*xxviii*
London	*GSE6532*	*50*	*0*	*0*	*100*	*0*	*100*	*0*	*50*	*87*	*2*	*0*	*2*	*0.0687*		*+*	*xxix*
expO	*GSE2109*	*35*	*33*	*42*	*83*					*301*	*22*	*0*	*22*	*0.1001*		*+*	*xxx*
Edinburgh	*GSE5462*					*0*	*100*	*0*		*116*	*1*	*0*	*1*	*0.1285*		*-*	*xxxi*
Oxford-Tamoxifen	*GSE6532*									*109*	*0*			*n.a.*			*xii*
TOTAL:		***48***	***25***	***68***	***72***	***57***	***12***	***31***	***35***	***3488***	***579***	***394***	***185***			***495***	

Additional datasets:

**Validation-Cohort-C**	*GSE21653*									*266*	*76*	*0*	*0*			*+*	*xxxii*
Normal breast biopsies	*GSE10780*									*140*							*xxxiii*

**** Remarks**: Datasets are sorted according to the comparability of the microarrays of only TNBC samples. The complete TransBIG dataset contains indepen*dent replicate samples from 19 patients of the Uppsala cohort and 22 patients of the Oxford-Untreated cohort. Affymetrix HG-U133A microarrays were applied in all studies except that in part HG-U133Plus arrays were used in datasets GSE2109, GSE3744, GSE6532, GSE9195, GSE10780, GSE12763, GSE12276, and GSE13787. In these cases only the probe sets identical to HG-U133A arrays were used.

References for Supplementary Table S1:

[i] Wang Y, Klijn JG, Zhang Y, Sieuwerts AM, Look MP, Yang F, Talantov D, Timmermans M, Meijer-van Gelder ME, Yu J, Jatkoe T, Berns EM, Atkins D, Foekens JA. Gene-expression profiles to predict distant metastasis of lymph-node-negative primary breast cancer. Lancet. 2005; 365(9460):671-9.

[ii] Minn AJ, Gupta GP, Padua D, Bos P, Nguyen DX, Nuyten D, Kreike B, Zhang Y, Wang Y, Ishwaran H, Foekens JA, van de Vijver M, Massagué J. Lung metastasis genes couple breast tumor size and metastatic spread. Proc Natl Acad Sci U S A. 2007 Apr 17;104(16):6740-5.

[iii] Creighton CJ, Kent Osborne C, van de Vijver MJ, Foekens JA, Klijn JG, Horlings HM, Nuyten D, Wang Y, Zhang Y, Chamness GC, Hilsenbeck SG, Lee AV, Schiff R. Molecular profiles of progesterone receptor loss in human breast tumors. Breast Cancer Res Treat. 2009 Mar;114(2):287-99.

[iv] Desmedt C, Piette F, Loi S, Wang Y, Lallemand F, Haibe-Kains B, Viale G, Delorenzi M, Zhang Y, d'Assignies MS, Bergh J, Lidereau R, Ellis P, Harris AL, Klijn JG, Foekens JA, Cardoso F, Piccart MJ, Buyse M, Sotiriou C; TRANSBIG Consortium. Strong time dependence of the 76-gene prognostic signature for node-negative breast cancer patients in the TRANSBIG multicenter independent validation series. Clin Cancer Res. 2007 Jun 1;13(11):3207-14.

[v] Schmidt M, Böhm D, von Törne C, Steiner E, Puhl A, Pilch H, Lehr HA, Hengstler JG, Kölbl H, Gehrmann M. The humoral immune system has a key prognostic impact in node-negative breast cancer. Cancer Res. 2008 Jul 1;68(13):5405-13.

[vi] Pawitan Y, Bjohle J, Amler L, Borg AL, Egyhazi S, Hall P, Han X, Holmberg L, Huang F, Klaar S, Liu ET, Miller L, Nordgren H, Ploner A, Sandelin K, Shaw PM, Smeds J, Skoog L, Wedren S, Bergh J. Gene expression profiling spares early breast cancer patients from adjuvant therapy: derived and validated in two population-based cohorts. Breast Cancer Res. 2005;7(6):R953-64.

[vii] Miller LD, Smeds J, George J, Vega VB, Vergara L, Ploner A, Pawitan Y, Hall P, Klaar S, Liu ET, Bergh J. An expression signature for p53 status in human breast cancer predicts mutation status, transcriptional effects, and patient survival. Proc Natl Acad Sci U S A. 2005 Sep 20;102(38):13550-5.

[viii] Ivshina AV, George J, Senko O, Mow B, Putti T, Smeds J, Lindahl T, Pawitan Y, Hall P, Nordgren H, Wong, John EL, Liu ET, Bergh J, Kuznetsov VA, Miller LD. Genetic reclassification of histologic grade delineates new clinical subtypes of breast cancer. Cancer Res. 2006 Nov 1;66(21):10292-301.

[ix] Rody A, Karn T, Solbach C, Gaetje R, Munnes M, Kissler S, Ruckhäberle E, Minckwitz GV, Loibl S, Holtrich U, Kaufmann M. The erbB2+ cluster of the intrinsic gene set predicts tumor response of breast cancer patients receiving neoadjuvant chemotherapy with docetaxel, doxorubicin and cyclophosphamide within the GEPARTRIO trial. Breast. 2007 Jun;16(3):235-40.

[x] Rody A, Holtrich U, Gaetje R, Gehrmann M, Engels K, von Minckwitz G, Loibl S, Diallo-Danebrock R, Ruckhäberle E, Metzler D, Ahr A, Solbach C, Karn T, Kaufmann M. Poor outcome in estrogen receptor-positive breast cancers predicted by loss of plexin B1. Clin Cancer Res. 2007 Feb 15;13(4):1115-22.

[xi] Minn AJ, Gupta GP, Siegel PM, Bos PD, Shu W, Giri DD, Viale A, Olshen AB, Gerald WL, Massagué J. Genes that mediate breast cancer metastasis to lung. Nature. 2005 Jul 28;436(7050):518-24.

[xii] Sotiriou C, Wirapati P, Loi S, Harris A, Fox S, Smeds J, Nordgren H, Farmer P, Praz V, Haibe-Kains B, Desmedt C, Larsimont D, Cardoso F, Peterse H, Nuyten D, Buyse M, Van de Vijver MJ, Bergh J, Piccart M, Delorenzi M. Gene expression profiling in breast cancer: understanding the molecular basis of histologic grade to improve prognosis. J Natl Cancer Inst. 2006 Feb 15;98(4):262-72.

[xiii] Rody A, Holtrich U, Muller V, et al. c-Kit: identification of coregulated genes by gene expression profiling and clinical relevance of two breast cancer subtypes with stem cell like features. 2006. ASCO Annual Meeting Proceedings Part I. J Clin Oncol 2006;24:622.

[xiv] Rody A, Karn T, Ruckhäberle E, Hanker L, Metzler D, Müller V, Solbach C, Ahr A, Gätje R, Holtrich U, Kaufmann M. Loss of Plexin B1 is highly prognostic in low proliferating ER positive breast cancers--results of a large scale microarray analysis. Eur J Cancer. 2009 Feb;45(3):405-13.

[xv] Yu K, Ganesan K, Tan LK, Laban M, Wu J, Zhao XD, Li H, Leung CH, Zhu Y, Wei CL, Hooi SC, Miller L, Tan P. A precisely regulated gene expression cassette potently modulates metastasis and survival in multiple solid cancers. PLoS Genet. 2008 Jul 18;4(7):e1000129.

[xvi] Hess KR, Anderson K, Symmans WF, Valero V, Ibrahim N, Mejia JA, Booser D, Theriault RL, Buzdar AU, Dempsey PJ, Rouzier R, Sneige N, Ross JS, Vidaurre T, Gómez HL, Hortobagyi GN, Pusztai L. Pharmacogenomic predictor of sensitivity to preoperative chemotherapy with paclitaxel and fluorouracil, doxorubicin, and cyclophosphamide in breast cancer. J Clin Oncol. 2006 Sep 10;24(26):4236-44.

[xvii] Richardson AL, Wang ZC, De Nicolo A, Lu X, Brown M, Miron A, Liao X, Iglehart JD, Livingston DM, Ganesan S. X chromosomal abnormalities in basal-like human breast cancer. Cancer Cell. 2006 Feb;9(2):121-32.

[xviii] Chen DT, Nasir A, Culhane A, Venkataramu C, Fulp W, Rubio R, Wang T, Agrawal D, McCarthy SM, Gruidl M, Bloom G, Anderson T, White J, Quackenbush J, Yeatman T. Proliferative genes dominate malignancy-risk gene signature in histologically-normal breast tissue. Breast Cancer Res Treat. 2009 Mar 6.

[xix] Bos PD, Zhang XH, Nadal C, Shu W, Gomis RR, Nguyen DX, Minn AJ, van de Vijver MJ, Gerald WL, Foekens JA, Massagué J. Genes that mediate breast cancer metastasis to the brain. Nature. 2009 Jun 18;459(7249):1005-9.

[xx] Popovici V, Chen W, Gallas BG, Hatzis C, Shi W, Samuelson FW, Nikolsky Y, Tsyganova M, Ishkin A, Nikolskaya T, Hess KR, Valero V, Booser D, Delorenzi M, Hortobagyi GN, Shi L, Symmans WF, Pusztai L. Effect of training sample size and classification difficulty on the accuracy of genomic predictors. Breast Cancer Res. 2010 Jan 11;12(1):R5.

[xxi] Farmer P, Bonnefoi H, Becette V, Tubiana-Hulin M, Fumoleau P, Larsimont D, Macgrogan G, Bergh J, Cameron D, Goldstein D, Duss S, Nicoulaz AL, Brisken C, Fiche M, Delorenzi M, Iggo R. Identification of molecular apocrine breast tumours by microarray analysis. Oncogene. 2005 Jul 7;24(29):4660-71.

[xxii] Gene Expression Omnibus. Series GSE12763 http://www.ncbi.nlm.nih.gov/geo/query/acc.cgi?acc=GSE12763

[xxiii] Ruckhäberle E, Rody A, Engels K, Gaetje R, von Minckwitz G, Schiffmann S, Grösch S, Geisslinger G, Holtrich U, Karn T, Kaufmann M. Microarray analysis of altered sphingolipid metabolism reveals prognostic significance of sphingosine kinase 1 in breast cancer. Breast Cancer Res Treat. 2008 Nov;112(1):41-52.

[xxiv] Chin K, DeVries S, Fridlyand J, Spellman PT, Roydasgupta R, Kuo WL, Lapuk A, Neve RM, Qian Z, Ryder T, Chen F, Feiler H, Tokuyasu T, Kingsley C, Dairkee S, Meng Z, Chew K, Pinkel D, Jain A, Ljung BM, Esserman L, Albertson DG, Waldman FM, Gray JW. Genomic and transcriptional aberrations linked to breast cancer pathophysiologies. Cancer Cell. 2006 Dec;10(6):529-41.

[xxv] Marty B, Maire V, Gravier E, Rigaill G, Vincent-Salomon A, Kappler M, Lebigot I, Djelti F, Tourdès A, Gestraud P, Hupé P, Barillot E, Cruzalegui F, Tucker GC, Stern MH, Thiery JP, Hickman JA, Dubois T. Frequent PTEN genomic alterations and activated phosphatidylinositol 3-kinase pathway in basal-like breast cancer cells. Breast Cancer Res. 2008;10(6):R101. Epub 2008 Dec 3.

[xxvi] Klein A, Wessel R, Graessmann M, Jürgens M, Petersen I, Schmutzler R, Niederacher D, Arnold N, Meindl A, Scherneck S, Seitz S, Graessmann A. Comparison of gene expression data from human and mouse breast cancers: identification of a conserved breast tumor gene set. Int J Cancer. 2007 Aug 1;121(3):683-8.

[xxvii] Zhang Y, Sieuwerts AM, McGreevy M, Casey G, Cufer T, Paradiso A, Harbeck N, Span PN, Hicks DG, Crowe J, Tubbs RR, Budd GT, Lyons J, Sweep FC, Schmitt M, Schittulli F, Golouh R, Talantov D, Wang Y, Foekens JA. The 76-gene signature defines high-risk patients that benefit from adjuvant tamoxifen therapy. Breast Cancer Res Treat. 2008 Sep 23. [Epub ahead of print]

[xxviii] Loi S, Haibe-Kains B, Desmedt C, Wirapati P, Lallemand F, Tutt AM, Gillet C, Ellis P, Ryder K, Reid JF, Daidone MG, Pierotti MA, Berns EM, Jansen MP, Foekens JA, Delorenzi M, Bontempi G, Piccart MJ, Sotiriou C. Predicting prognosis using molecular profiling in estrogen receptor-positive breast cancer treated with tamoxifen. BMC Genomics. 2008 May 22;9:239.

[xxix] Loi S, Haibe-Kains B, Desmedt C, Lallemand F, Tutt AM, Gillet C, Ellis P, Harris A, Bergh J, Foekens JA, Klijn JG, Larsimont D, Buyse M, Bontempi G, Delorenzi M, Piccart MJ, Sotiriou C. Definition of clinically distinct molecular subtypes in estrogen receptor-positive breast carcinomas through genomic grade. J Clin Oncol. 2007 Apr 1;25(10):1239-46.

[xxx] The International Genomics Consortium (IGC). The expO project (Expression Project For Oncology) http://www.intgen.org/

[xxxi] Miller WR, Larionov AA, Renshaw L, Anderson TJ, White S, Murray J, Murray E, Hampton G, Walker JR, Ho S, Krause A, Evans DB, Dixon JM. Changes in breast cancer transcriptional profiles after treatment with the aromatase inhibitor, letrozole. Pharmacogenet Genomics. 2007 Oct;17(10):813-26.

[xxxii] Sabatier R, Finetti P, Cervera N, Lambaudie E, Esterni B, Mamessier E, Tallet A, Chabannon C, Extra JM, Jacquemier J, Viens P, Birnbaum D, Bertucci F. A gene expression signature identifies two prognostic subgroups of basal breast cancer. Breast Cancer Res Treat. 2010 May 21. [Epub ahead of print]

[xxxiii] Chen DT, Nasir A, Culhane A, Venkataramu C, Fulp W, Rubio R, Wang T, Agrawal D, McCarthy SM, Gruidl M, Bloom G, Anderson T, White J, Quackenbush J, Yeatman T. Proliferative genes dominate malignancy-risk gene signature in histologically-normal breast tissue. Breast Cancer Res Treat. 2010 Jan;119(2):335-46.

Supplementary Table S2: **Comparison of clinical parameters of TNBC with basal-like breast cancer (BLBC) or non-BLBC phenotype in the validation *cohorts -B and –C***

		Cohort-B					*Cohort-C*				
**Parameter**		**informa-tion available***	**total (n=185)**	**non-BLBC (n=50, 27.0%)**	**BLBC (n=135, 73.0%)**	**P-Value**	**informa-tion available***	**total (n=76)**	**non-BLBC (n=23, 30.3%)**	**BLBC (n=53, 69.7%)**	**P-Value**
lymph node status	LNN	n=96	36	11 (35.5%)	25 (38.5%)	n.s. (0.8)	n=76	44	12 (52.2%)	32 (60.4%)	n.s. (0.6)
	N1		60	20 (64.5%)	40 (61.5%)			32	11 (47.8%)	21 (39.6%)	
Age 50 yrs	≤ 50 yr	n=140	66	13 (33.3%)	53 (52.5%)	0.058	n=76	27	3 (13.0%)	24 (45.3%)	0.009
	> 50 yr		74	26 (66.7%)	48 (47.5%)			49	20 (87.0%)	29 (54.7%)	
Tumor size	≤ 2 cm	n=151	29	7 (16.7%)	22 (20.2%)	n.s. (0.8)	n=73	11	3 (13.6%)	8 (15.7%)	n.s. (1.0)
	> 2 cm		122	35 (83.3%)	87 (79.8%)			62	19 (86.4%)	43 (84.3%)	
Histological grade	G3	n=156	110	21 (55.3%)	89 (75.4%)	0.024	n=76	62	18 (78.3%)	44 (83.0%)	n.s. (0.8)
	G1&2		46	17 (44.7%)	29 (24.6%)			14	5 (21.7%)	9 (17.0%)	

* *Number of cases with available information on the specific parameter*

Supplementary Table S3: Correlation of the BLBC phenotype with other metagenes in TNBC

**Metagene**	**Correlation**	**Pearson-R, P-Value**		
		finding cohort-A (n=394)	validation cohort-B (n=185)	validation cohort-C (n=76)
Proliferation	positive	R=0.371, P<0.001	R=0.372, P<0.001	R=0.249, P=0.030
VEGF	positive	R=0.151, P=0.003	R=0.121, P=0.099*	R=0.249, P=0.030
Apocrine	negative	R=-0.401, P<0.001	R=-0.533, P<0.001	R=-0.474, P<0.001
MHC-2	negative	R=-0.233, P<0.001	R=-0.140, P=0.057*	R=-0.100, P=n.s.
Adipocyte	negative	R=-0.182, P<0.001	R=-0.184, P=0.012	R=0.064, P=n.s.
T-Cell	negative	R=-0.161, P=0.001	R=-0.129, P=0.079*	R=-0.064, P=n.s.

** metagene only trended to significance in the validation cohort*

Supplementary Table S4: **Univariate Cox Regression of metagenes as continous variables for event free survival in the finding cohort-A:**

Metagene	B	SE	Wald statistic	P-Value*
**IL-8**	**103.1**	**27.6**	**13.91**	**<0.001**
**Histone**	**106.1**	**34.9**	**9.25**	**0.002**
**VEGF**	**94.5**	**35.9**	**6.92**	**0.009**
**B-Cell**	**-44.7**	**19.4**	**5.30**	**0.021**
**T-Cell**	**-80.1**	**36.0**	**4.96**	**0.026**
Proliferation	37.3	45.6	0.67	0.414
Basal-like	15.7	29.7	0.28	0.597
Claudin-CD24	16.8	32.8	0.26	0.609
Apocrine	10.7	26.4	0.17	0.684
Adipocyte	-24.2	22.0	1.21	0.272
Stroma §	74.4 (76.4)	41.8 (43.3)	3.17 (3.11)	0.075 (0.078)
IFN	-13.1	35.7	0.14	0.713
MHC-1	-90.5	47.3	3.66	0.056
MHC-2	-61.0	33.2	3.37	0.066
Hemoglobin §	16.0 (22.6)	24.7 (29.8)	0.42 (0.58)	0.518 (0.447)
HOXA	-35.3	36.9	0.91	0.340

* significant P-Values are given in bold

§ for Stroma and Hemoglobin metagenes the results of the analysis including only surgical biopsies are given in parentheses

Supplementary Table S5: **Result of stepwise Cox Regression model including all metagenes as continous variables for event free survival in the finding cohort-A:**

Metagene*	B	SE	Wald statistic	P-Value*
IL-8	115.6	28.6	16.34	<0.001
Histone	125.6	34.8	13.02	<0.001
B-Cell	-56.6	8.2	8.23	0.004

* Only IL-8, Histone, and B-Cell metagenes remained in the final step because of their independent effect on event free survival. All 13 other metagenes were excluded from the stepwise cox regression model based on the P<0.05 criterion.

Supplementary Table S6: Comparison of the BLBC classification based on distribution of the basal-like metagene to the centroid method for subtype definition

SSP-prediction in cohort of all subtypes (n=1364 total, n=172 TNBC) *

**metagene definition**	**Centroid-Single Sample Predictor (data not centered)**			
	unclassified	Basal-like	other subtype	TOTAL
Non-BLBC	30 (54.5%)	13 (23.6%)	12 (21.8%)	55
BLBC	16 (13.7%)	98 (83.8%)	3 (2.5%)	117
				172
**metagene definition**	**Centroid-Single Sample Predictor (centered data)**			
	unclassified	Basal-like	other subtype	TOTAL
Non-BLBC	0	36 (65.5%)	19 (34.5%)	55
BLBC	0	114 (97.4%)	3 (2.6%)	117
				172

SSP-prediction in pure TNBC cohort (n=579) †

**metagene definition**	**Centroid-Single Sample Predictor (data not centered)**			
	unclassified	Basal-like	other subtype	TOTAL
Non-BLBC	107 (68.2%)	32 (20.4%)	18 (11.4%)	157
BLBC	50 (11.8%)	364 (86.3%)	8 (1.9%)	422
				579

* A nearest centroid single sample predictor (SSP) method either with or without centering as described by Weigelt et al. (2010, Lancet Oncol; 11:339) was applied to seven larger datasets (Frankfurt, Mainz, NewYork, Stockholm, Transbig, Uppsala, Rotterdam) to assign a total of 1364 breast cancer samples to a molecular subtype (according to Hu et al. 2006, BMC Genomics, 7:96). The data for the 172 TNBC samples from this cohort that are included in our study are shown. Results are in line with data from Weigelt et al. that the basal-subtype is the most reproducible when using the centroid method. More than 97% of samples defined as BLBC based on the metagene distribution are assigned to basal-like or "unclassified" by the centroid method. The majority of Non-BLBC samples based on the metagene distribution are also assigned to the unclassified or basal-like group by the centroid method depending on the variant of the method (non-centered or centered data, respectively).

† Application of the centroid method to the cohorts-A and -B of 579 TNBC samples (without non-TNBC samples) results in a positive predictive value (PPV) of 86.3 % for the prediction of the Basal-like SSP-subtype from the distribution of the metagene. Only 20.4 % of the Non-BLBC samples based on metagene distribution were assigned to Basal-like subtype by the centroid method, most are unclassified. Data centering is not applicable to a complete ER negative cohort as described (Lusa et al. 2007, J Natl Cancer Inst. 99:1715).

Supplementary Table S7:

List of 355 Affymetrix probe sets used for metagene calculation:

Metagene	Affymetrix ProbeSet	GeneSymbol	DatasetBias KruskalFindCohort
IL-8	204470_at	CXCL1	14.8
IL-8	202859_x_at	IL8	35.9
IL-8	211506_s_at	IL8	54
IL-8	209774_x_at	CXCL2	18.1
VEGF	200632_s_at	NDRG1	40
VEGF	210513_s_at	VEGF	46.8
VEGF	211527_x_at	VEGF	72.4
VEGF	210512_s_at	VEGF	45.2
VEGF	212171_x_at	VEGF	60.8
VEGF	202912_at	ADM	26.8
VEGF	221009_s_at	ANGPTL4	36.8
Proliferation	206102_at	KIAA0186	50.1
Proliferation	209172_s_at	CENPF	39.5
Proliferation	203418_at	CCNA2	42.7
Proliferation	204026_s_at	ZWINT	42.2
Proliferation	203213_at	CDC2	56.6
Proliferation	203214_x_at	CDC2	47.6
Proliferation	210559_s_at	CDC2	45.6
Proliferation	204170_s_at	CKS2	79.1
Proliferation	204092_s_at	STK6	55
Proliferation	208079_s_at	STK6	38.9
Proliferation	203362_s_at	MAD2L1	43.9
Proliferation	213226_at	CCNA2	59.5
Proliferation	202095_s_at	BIRC5	61.3
Proliferation	202613_at	CTPS	49
Proliferation	201291_s_at	TOP2A	92.1
Proliferation	201292_at	TOP2A	31.5
Proliferation	222039_at	LOC146909	49.7
Proliferation	204822_at	TTK	52.2
Proliferation	202954_at	UBE2C	31.8
Proliferation	203755_at	BUB1B	32.3
Proliferation	202705_at	CCNB2	35.5
Proliferation	204962_s_at	CENPA	65.2
Proliferation	209642_at	BUB1	46.5
Proliferation	202870_s_at	CDC20	44.2
Proliferation	209408_at	KIF2C	46.5
Proliferation	221520_s_at	CDCA8	50.7
Proliferation	218039_at	NUSAP1	24.5
Proliferation	206364_at	KIF14	47.2
Proliferation	204641_at	NEK2	28
Proliferation	207828_s_at	CENPF	43.6
Proliferation	219918_s_at	ASPM	36.8
Proliferation	204444_at	KIF11	45
Proliferation	218542_at	C10orf3	44.5
Proliferation	213008_at	FLJ10719	67.4
Proliferation	213007_at	FLJ10719	43.9
Proliferation	218009_s_at	PRC1	43.3
Proliferation	210052_s_at	TPX2	11.3
Proliferation	203764_at	DLG7	53
Proliferation	214710_s_at	CCNB1	49
Proliferation	218355_at	KIF4A	60
Proliferation	202580_x_at	FOXM1	35.4
Proliferation	221436_s_at	CDCA3	31.2
Proliferation	218755_at	KIF20A	24.8
Proliferation	218663_at	HCAP-G	49.2
Proliferation	219148_at	PBK	51.1
Proliferation	218585_s_at	RAMP	36.8
Proliferation	218726_at	DKFZp762E1312	52.2

(continued)

Metagene	Affymetrix ProbeSet	GeneSymbol	DatasetBias KruskalFindCohort
Basal-like	202341_s_at	TRIM2	149.9
Basal-like	202342_s_at	TRIM2	46
Basal-like	215945_s_at	TRIM2	65.5
Basal-like	203074_at	ANXA8	26
Basal-like	202504_at	TRIM29	18.4
Basal-like	211002_s_at	TRIM29	19.6
Basal-like	204268_at	S100A2	26.3
Basal-like	201820_at	KRT5	19.5
Basal-like	204855_at	SERPINB5	31.7
Basal-like	209351_at	KRT14	13.2
Basal-like	205157_s_at	KRT17	19
Basal-like	212236_x_at	KRT17	17.1
Basal-like	209800_at	KRT16	42.2
Basal-like	209126_x_at	KRT6B	26.7
Basal-like	213680_at	KRT6B	19.8
Basal-like	209125_at	KRT6A	26.6
Basal-like	214580_x_at	KRT6A	20.1
Basal-like	1438_at	EPHB3	55.6
Basal-like	204600_at	EPHB3	19.3
Basal-like	218176_at	MAGEF1	64.5
Basal-like	205044_at	GABRP	24.8
Basal-like	202035_s_at	SFRP1	70.3
Basal-like	202036_s_at	SFRP1	51.7
Basal-like	202037_s_at	SFRP1	38.2
Basal-like	209842_at	SOX10	33.6
Basal-like	220425_x_at	ROPN1	39.6
Basal-like	206560_s_at	MIA	47.4
Basal-like	209843_s_at	SOX10	64.4
Basal-like	220624_s_at	ELF5	52.1
Basal-like	220625_s_at	ELF5	38.1
Basal-like	212730_at	DMN	33.5
Basal-like	219615_s_at	KCNK5	21.8
Basal-like	209504_s_at	PLEKHB1	25.3
Basal-like	213260_at	FOXC1	26.7
Basal-like	218963_s_at	KRT23	27.2
Basal-like	205487_s_at	VGLL1	25.3
Basal-like	215729_s_at	VGLL1	30.5
Claudin-CD24	201650_at	KRT19	34.3
Claudin-CD24	201596_x_at	KRT18	29.7
Claudin-CD24	209008_x_at	KRT8	20.4
Claudin-CD24	209016_s_at	KRT7	25.7
Claudin-CD24	205980_s_at	ARHGAP8	76
Claudin-CD24	37117_at	ARHGAP8	52.6
Claudin-CD24	203953_s_at	CLDN3	54
Claudin-CD24	203954_x_at	CLDN3	30.8
Claudin-CD24	201428_at	CLDN4	44.2
Claudin-CD24	201839_s_at	TACSTD1	45.1
Claudin-CD24	218186_at	RAB25	16.1
Claudin-CD24	201510_at	ELF3	13.8
Claudin-CD24	210827_s_at	ELF3	46.7
Claudin-CD24	208650_s_at	CD24	48.9
Claudin-CD24	209772_s_at	CD24	47.8
Claudin-CD24	208651_x_at	CD24	40.9
Claudin-CD24	209771_x_at	CD24	71
Claudin-CD24	216379_x_at	CD24	70.1
Claudin-CD24	266_s_at	CD24	48.5
Apocrine	204941_s_at	ALDH3B2	76.4
Apocrine	204942_s_at	ALDH3B2	36.4
Apocrine	211110_s_at	AR	48.6
Apocrine	211621_at	AR	90.5
Apocrine	209173_at	AGR2	39
Apocrine	207131_x_at	GGT1	49.5
Apocrine	208284_x_at	GGT1	46.6
Apocrine	209919_x_at	GGT1	40.9
Apocrine	211417_x_at	GGT1	57.1
Apocrine	215603_x_at	GGT1	73.9
Apocrine	211416_x_at	GGTLA4	81.7
Apocrine	206463_s_at	DHRS2	57.4
Apocrine	214079_at	DHRS2	65.9
Apocrine	211682_x_at	UGT2B28	20.4
Apocrine	206714_at	ALOX15B	69.3
Apocrine	206509_at	PIP	47.3
Apocrine	204667_at	FOXA1	70.6
Apocrine	218211_s_at	MLPH	74.2
Apocrine	214451_at	TFAP2B	42.3
Apocrine	204607_at	HMGCS2	65.2
Apocrine	214243_s_at	SERHL	32.5
Apocrine	217276_x_at	dJ222E13.1	38.5
Apocrine	217284_x_at	dJ222E13.1	53.1
Apocrine	213441_x_at	SPDEF	48.2
Apocrine	214404_x_at	SPDEF	31.9
Apocrine	220192_x_at	SPDEF	22.8
Apocrine	215686_x_at	TFAP2B	30.1

(continued)

Metagene	Affymetrix ProbeSet	GeneSymbol	DatasetBias KruskalFindCohort
Histone	208583_x_at	HIST1H2AJ	55.5
Histone	208523_x_at	HIST1H2BI	44.7
Histone	209398_at	HIST1H1C	26.8
Histone	208180_s_at	H4FH	25.2
Histone	202708_s_at	HIST2H2BE	31.4
Histone	208546_x_at	HIST1H2BH	28.2
Histone	208490_x_at	HIST1H2BF	15.8
Histone	208527_x_at	HIST1H2BE	33.1
Histone	208579_x_at	H2BFS	54.9
Histone	209806_at	HIST1H2BK	20.3
Histone	209911_x_at	HIST1H2BD	46.3
Histone	222067_x_at	HIST1H2BD	17.1
Histone	214290_s_at	HIST2H2AA	17.1
Histone	218280_x_at	HIST2H2AA	18.1
Histone	215071_s_at	HIST1H2AC	18
Histone	210387_at	HIST1H2BG	66.7
Histone	215779_s_at	HIST1H2BG	56.5
Histone	214469_at	HIST1H2AE	21.1
Histone	214455_at	H2BFL	25.6
Adipocyte	203980_at	FABP4	21
Adipocyte	205913_at	PLIN	19.2
Adipocyte	207175_at	ADIPOQ	21.9
Adipocyte	209612_s_at	ADH1B	43.5
Adipocyte	209613_s_at	ADH1B	42.6
Adipocyte	206488_s_at	CD36	36.1
Adipocyte	209555_s_at	CD36	24.6
Adipocyte	209763_at	CHRDL1	55.8
Stroma	202766_s_at	FBN1	114.7
Stroma	207172_s_at	CDH11	127.4
Stroma	207173_x_at	CDH11	139.1
Stroma	200665_s_at	SPARC	115.1
Stroma	202465_at	PCOLCE	151.3
Stroma	201185_at	PRSS11	119.3
Stroma	201069_at	MMP2	130.7
Stroma	202273_at	PDGFRB	170.7
Stroma	204114_at	NID2	136
Stroma	201792_at	AEBP1	136.4
Stroma	201744_s_at	LUM	76.5
Stroma	201438_at	COL6A3	153.1
Stroma	202310_s_at	COL1A1	135.5
Stroma	202403_s_at	COL1A2	107.9
Stroma	202404_s_at	COL1A2	100.2
Stroma	201852_x_at	COL3A1	153.7
Stroma	215076_s_at	COL3A1	115.2
Stroma	211161_s_at	COL3A1	106.2
Stroma	221729_at	COL5A2	118.8
Stroma	221730_at	COL5A2	131.2
Stroma	202311_s_at	COL1A1	129.1
Stroma	203325_s_at	COL5A1	109.9
Stroma	212488_at	COL5A1	127.5
Stroma	212489_at	COL5A1	140
Stroma	210809_s_at	POSTN	86.2
Stroma	212667_at	SPARC	106.6
Stroma	209596_at	DKFZp564I1922	99.6
Stroma	209955_s_at	FAP	100
Stroma	201893_x_at	DCN	113.2
Stroma	211896_s_at	DCN	113.1
Stroma	211813_x_at	DCN	133.7
Stroma	209335_at	DCN	90
Stroma	213001_at	ANGPTL2	144.6
Stroma	213004_at	ANGPTL2	133.1
Stroma	208851_s_at	THY1	108.4
Stroma	213869_x_at	THY1	94.2
Stroma	213909_at	LRRC15	84.9
Stroma	204619_s_at	CSPG2	101.6
Stroma	204620_s_at	CSPG2	125.8
Stroma	221731_x_at	CSPG2	97.8
Stroma	221541_at	LCRISP2	111
Stroma	211571_s_at	CSPG2	185
Stroma	215646_s_at	CSPG2	173.6
Stroma	211719_x_at	FN1	113.2
Stroma	210495_x_at	FN1	121.8
Stroma	216442_x_at	FN1	113
Stroma	212464_s_at	FN1	107.1
IFN	202411_at	IFI27	22.2
IFN	202086_at	MX1	27
IFN	205483_s_at	G1P2	27.1
IFN	203153_at	IFIT1	29.8
IFN	204747_at	IFIT3	32.2
IFN	213797_at	RSAD2	44.9
IFN	204439_at	IFI44L	30.8
IFN	214453_s_at	IFI44	26
IFN	205552_s_at	OAS1	62
IFN	204972_at	OAS2	68.8
IFN	218400_at	OAS3	48.7
IFN	219352_at	HERC6	28.3
IFN	205660_at	OASL	28.5
IFN	210797_s_at	OASL	46.5

Metagene	Affymetrix ProbeSet	GeneSymbol	DatasetBias KruskalFindCohort
MHC-1	200905_x_at	HLA-E	49.9
MHC-1	217456_x_at	HLA-E	66.5
MHC-1	210514_x_at	HLA-G	20.6
MHC-1	209140_x_at	HLA-B	87.1
MHC-1	208812_x_at	HLA-B	79.9
MHC-1	216526_x_at	HLA-C	67.2
MHC-1	214459_x_at	HLA-C	53.7
MHC-1	211529_x_at	HLA-G	19.4
MHC-1	211528_x_at	HLA-G	43
MHC-1	208729_x_at	HLA-B	40.9
MHC-1	211911_x_at	HLA-B	43.3
MHC-1	217436_x_at	HLA-J	42
MHC-1	204806_x_at	HLA-F	21.7
MHC-1	221875_x_at	HLA-F	26.9
MHC-1	211799_x_at	HLA-A	35.8
MHC-1	213932_x_at	HLA-A	60.9
MHC-1	215313_x_at	HLA-A	63.9
T-Cell	209083_at	CORO1A	65.3
T-Cell	204891_s_at	LCK	44.9
T-Cell	206666_at	GZMK	36.2
T-Cell	201720_s_at	LAPTM5	102
T-Cell	201721_s_at	LAPTM5	46
T-Cell	204912_at	IL10RA	24.1
T-Cell	206150_at	TNFRSF7	49.1
T-Cell	204563_at	SELL	63.7
T-Cell	209670_at	TRA@	41.6
T-Cell	204118_at	CD48	40.3
T-Cell	205831_at	CD2	36.8
T-Cell	210915_x_at	TRBC1	36.9
T-Cell	213193_x_at	TRBC1	39.6
T-Cell	213539_at	CD3D	50.5
T-Cell	211796_s_at	TRBC1	46.9
T-Cell	211339_s_at	ITK	27.4
T-Cell	203416_at	CD53	30.6
T-Cell	211742_s_at	EVI2B	29.4
T-Cell	212588_at	PTPRC	31.2
T-Cell	209671_x_at	TRA@	72
T-Cell	210972_x_at	TRA@	71.2
T-Cell	211902_x_at	TRA@	87.3
T-Cell	220330_s_at	SAMSN1	32.7
T-Cell	38149_at	KIAA0053	42.6
T-Cell	219014_at	PLAC8	27.7
T-Cell	204661_at	CD52	54.7
T-Cell	34210_at	CD52	37.8
MHC-2	201137_s_at	HLA-DPB1	22.2
MHC-2	203932_at	HLA-DMB	38.9
MHC-2	209619_at	CD74	50.4
MHC-2	204670_x_at	HLA-DRB1	27.2
MHC-2	208306_x_at	HLA-DRB1	31.8
MHC-2	209312_x_at	HLA-DRB1	27.1
MHC-2	215193_x_at	HLA-DRB1	29.8
MHC-2	208894_at	HLA-DRA	35.5
MHC-2	210982_s_at	HLA-DRA	48.6
MHC-2	211991_s_at	HLA-DPA1	50.7
MHC-2	212671_s_at	HLA-DQA1	42
MHC-2	217478_s_at	HLA-DMA	35
MHC-2	212998_x_at	HLA-DQB1	48.1
MHC-2	211654_x_at	HLA-DQB1	29.1

(continued)

Metagene	Affymetrix ProbeSet	GeneSymbol	DatasetBias KruskalFindCohort
B-Cell	211639_x_at	IGHM	48.5
B-Cell	211633_x_at	IGHG1	65.6
B-Cell	211641_x_at	IGHG1	52.2
B-Cell	211634_x_at	IGHM	45.8
B-Cell	211635_x_at	IGHG3	44.1
B-Cell	211640_x_at	IGHG1	41.1
B-Cell	211798_x_at	IGLJ3	57.6
B-Cell	211881_x_at	IGLJ3	51.5
B-Cell	211637_x_at	LOC388078	51.2
B-Cell	216491_x_at	IGHM	54
B-Cell	211908_x_at	IGHG1	58.8
B-Cell	211650_x_at	IGHG1	46.4
B-Cell	216510_x_at	IGHG1	60.5
B-Cell	217281_x_at	IGHG1	60.3
B-Cell	211643_x_at	IGKC	32.5
B-Cell	213502_x_at	LOC91316	25.6
B-Cell	211430_s_at	IGH@	21.9
B-Cell	209138_x_at	IGLC2	18.1
B-Cell	214677_x_at	IGL@	18.1
B-Cell	215121_x_at	IGL@	21.3
B-Cell	215379_x_at	IGL@	15
B-Cell	221651_x_at	IGKC	28.2
B-Cell	221671_x_at	IGKC	27
B-Cell	211644_x_at	IGKC	36.9
B-Cell	214669_x_at	LOC440871	19.4
B-Cell	211645_x_at	IGKC	29.4
B-Cell	215176_x_at	IGKC	31.4
B-Cell	217378_x_at	LOC391427	35.1
B-Cell	217157_x_at	IGKC	35.3
B-Cell	214836_x_at	IGKC	20
B-Cell	216207_x_at	IGKV1D-13	27.8
B-Cell	217480_x_at	LOC339562	30.3
B-Cell	216576_x_at	- - -	32.6
B-Cell	216401_x_at	- - -	35.9
B-Cell	215946_x_at	IGLL1	30.5
B-Cell	214916_x_at	IGH@	46.3
B-Cell	216557_x_at	IGHG1	62.6
B-Cell	211868_x_at	IGHG1	46.5
B-Cell	216984_x_at	IGLJ3	46
B-Cell	217148_x_at	IGLC2	41
B-Cell	216542_x_at	MGC27165	68.6
B-Cell	214768_x_at	IGKC	27.7
B-Cell	214973_x_at	IGHD	54.8
B-Cell	217235_x_at	IGLJ3	44.3
B-Cell	215949_x_at	IGHM	69.1
B-Cell	214777_at	IGKC	39
B-Cell	217179_x_at	IGL@	26
Hemoglobin	204419_x_at	HBG2	133.7
Hemoglobin	204848_x_at	HBG1	115.9
Hemoglobin	209116_x_at	HBB	164.4
Hemoglobin	204018_x_at	HBA1	158.4
Hemoglobin	209458_x_at	HBA1	154.7
Hemoglobin	211745_x_at	HBA1	150.8
Hemoglobin	214414_x_at	HBA2	152
Hemoglobin	211696_x_at	HBB	155.4
Hemoglobin	217232_x_at	HBB	155.2
Hemoglobin	217414_x_at	HBA2	153.7
Hemoglobin	211699_x_at	HBA1	147.7
Hemoglobin	213515_x_at	HBG2	158.6
HOXA	206289_at	HOXA4	27.4
HOXA	206847_s_at	HOXA7	43.9
HOXA	209905_at	HOXA9	41.1
HOXA	214651_s_at	HOXA9	27.8
HOXA	213844_at	HOXA5	38.2
HOXA	213147_at	HOXA10	30.1
HOXA	213150_at	HOXA10	20.9
HOXA	213823_at	HOXA11	43
