# Supplementary material for: A clinically relevant gene signature in triple negative and basal-like breast cancer
Source: Breast Cancer Res. 2011 Oct 6;13(5):R97. doi: 10.1186/bcr3035 (PMC3262210; doi:10.1186/bcr3035)
Supplement: Additional file 4 — Supplementary Methods. An Adobe file containing supplementary information on methodology and six additional supplementary figures (S16 to S21), which are referred to within this supplementary methods. [file bcr3035-S4.PDF]

Supplementary Methods

All analyses were performed according to the REMARK recommendations for tumor marker studies[A]. A respective diagram of the complete analytical strategy and the flow of patients through the study, including the number of patients included in each stage of the analysis is given in Supplementary Figure S1. The analyses were performed using Bioconductor (http://www.bioconductor.org/) and the R software environment (http://www.r-project.org/) and SPSS version 17.0. The genefu package was used for implementation of published gene signatures (http://www.bioconductor.org /packages/release/bioc/html/genefu.html). Fishers exact test was applied for the analysis of associations between categorical parameters. All reported P values are two sided and P values of less than 0.05 were considered to indicate a significant result.

1. Assembly of microarray data:

To assemble a highly homogeneous dataset of microarrays of triple negative breast cancers we used (i) only one single array platform (Affymetrix U133A and U133Plus2) and (ii) included only samples defined as triple negative by a consistent method based on the gene expression itself of ER, PR, and HER2 as previously described[B,C]. For a reasonable sample size pooling datasets was necessary. A major concern of this procedure are systematic technical differences between individual datasets ("batch effects"). Many adaption methods as e.g. Z-normalization often do not eliminate but rather blur such effects. Thus we applied two further strategies to cope with this problem. First, we rigorously selected only highly comparable datasets for the finding cohort. Second, we controlled for biased genes which still show associations with the dataset vector. These two strategies are described below:

Affymetrix microarray data from 28 datasets encompassing a total of n=3488 primary breast cancers were assembled. Expression data were analyzed using the MAS5.0 algorithm[D] of the *affy* package[E] of

the Bioconductor software project[F]. Subsequently data were $\log_2$-transformed, median-centered across arrays, and the expression values of all the probesets from the U133A array were multiplied by a scale factor *S* so that the magnitude (sum of the squares of the values) equals one. Triple negative breast cancers (TNBC, n=579) were identified based on gene expression of ER, PgR, and HER2 on microarray as described previously[C]. Gene expression data of these 579 TNBC have been deposited into the GEO database (accession number GSE31519). To select only comparable samples for the finding cohort a metric to compare different datasets was developed. We derived a simple comparability metric *C* from the sum of the squared differences of the mean (μ) within a specific dataset and among all datasets, respectively, normalized by the standard deviation (σ) calculated for all genes (*g*) on the array:

$$C_{dataset_i} = \sum_{g=1}^{n} \left(\frac{\mu_{g,\, dataset_i} - \mu_{g,\, total}}{\sigma_{g,\, total}} \right)^2$$

As shown in Supplementary Figure S2 all datasets were sorted according to this metric and those 15 datasets with lowest values (norm. $C \leq 0.03$; see also Suppl. Figure S16) encompassing n=394 samples selected as finding cohort-**A** (Supplementary Figure S2). The excluded datasets encompassing n=185 samples were withhold as validation cohort-**B** (Supplementary Figure S1).

Since the number of patients with follow up in the validation cohort-**B** revealed as too small (n=30) to validate the final prognostic predictor an additional independent validation cohort-**C**[G] was also analyzed for validation. However, this cohort-C was not available and thus never touched during the preceding analysis steps (Supplementary Figure S1). Clinical data of all three cohorts are given in Table 1.

2. Building of metagenes for principal molecular phenotypes among TNBC

High feature to sample ratio is one of the most severe limitations of genomic profiling methods leading to an inflation of α-values[H]. Therefore, unsupervised clustering was applied for feature reduction based on the assumption that the expression of a large number of genes is highly inter-

dependent. This dependence can be attributed to the expression of sets of genes in different cell types in the sample or to differentiation steps and pathways associated with specific expression profiles. The command line version of the CLUSTER 3.0 program was applied using pairwise single-linkage and Pearson correlation as distance metric (available at http://bonsai.ims.u-tokyo.ac.jp/~mdehoon/software/cluster). As described previously, genes which did not show a correlation with other genes above a certain threshold (0.5) were suspected to represent noise and therefore discarded from further analysis[I]. To build "metagenes" for the principal vectors we selected only those clusters which contained at least 10 elements and a minimal average correlation of 0.7 or 25 elements with a correlation of 0.6, respectively. This strategy rediscovered previously described gene clusters for basal-like genes[J], an apocrine/androgen receptor signalling signature[K,L], a stromal signature[M,N], the claudin-CD24 signature[O,P] as well as several gene clusters related different types of immune and blood cells[I,J,Q,R,S,T,U],. A higher cutoff of 0.8 for correlation was applied to differentiate these individual subclusters associated with distinct types of immune cells. In addition we included four metagenes for clusters containing less than 10 elements but which are well known for their biological phenotype, namely angiogenesis[V],[S] , adipocytes[J], inflammation[W,X,Y], and a cluster of HOXA genes (Table 2 and Figure 1). Metagene expression values were determined by calculating the mean of the normalized expression values of all probesets in the respective cluster as previously described[I]. A list of all 355 applied Affymetrix probesets is given in Supplementary Table S7.

3. Control for biased probesets and metagenes

We aimed to control for metagenes which display a possible bias related to technical differences between datasets. As a metric for the dependence of each individual probeset on the dataset vector we used the standard Kruskal-Wallis rank test. The distribution of the rank sum statistics for all 22,283 probesets from the U133A array in the finding cohort-A of n=394 samples is shown in Supplementary Figure S17A. We controlled for this dataset bias throughout the analysis by tagging each of the 22,283 Affy probesets with its Kruskal stat value. Thereby the influence of dataset bias

was monitored during unsupervised analyses. Biased probesets tend to cluster together and can easily be detected by their tagged Kruskal stats. The median Kruskal-Wallis rank sum statistic and inter quartile range (IQR) of the probesets of the 16 metagenes from Table 2 are presented in Supplementary Figure S17B. The values for each individual probeset are given in Supplementary Table S7. Only the *Stroma* and *Hemoglobin* metagenes display a high bias between datasets. This effect originates from the inclusion of two datasets which were obtained from fine needle aspiration (FNA) samples (Supplementary Figure S3). Such samples generally contain relative high amounts of blood and low amounts of stromal tissue. Thus to avoid a dataset bias in all analyses concerning the *Stroma* and *Hemoglobin* metagenes four datasets with FNA and core biopsies were excluded and only datasets containing surgical biopsy samples (n=365 and n=130 in the finding and validation cohorts-A and -B, respectively) were used (Supplementary Table S1).

4. Control for platform bias between U133A and U133Plus2.0 Affymetrix arrays

A platform bias between U133A and U133Plus2.0 arrays has been reported in a previous study by Symmans et al. 2010[Z] for SET index gene signature (Suppl. Fig. S3 in the Supplementary Appendix of Symmans et al) and a correction factor was used in this study to adjust for this bias. In contrast, no platform bias was observed for ESR1 and HER2 gene expression in the same study (Suppl. Fig. S4 of Symmans et al.). We obtained similar results when we compared the distribution of ESR1, PgR, and HER2 gene expression from samples profiled on either U133A or U133Plus2.0 arrays in our full cohort of 3488 samples. As demonstrated in Supplementary Figure S18A no platform bias was observed. We have also observed a high consistency of cutoffs for ER, PgR, and HER2 genes in different datasets previously[C]. Therefore the primary selection of the triple negative cohort of 579 samples is not influenced by the type of array used in the respective individual studies. 118 (20.4%) of the 579 TNBC were profiled on U133 Plus 2.0 arrays, but only 21 (5.3%) of the 394 samples from the finding cohort-A. The bias between the two different U133 platforms described in the report by Symmans et al is not systematic but only effects certain probesets since no effect on ESR1, PgR, and HER2 was

observed. Thus any adaption has to be done on a gene by gene (or metagene) basis. Therefore we checked the inter-platform agreement as well as the inter-laboratory agreement of the metagenes analyzed in our study using the raw data from a 2x2 factorial study from the Symmans et al publication (GEO accession number GSE17700). As shown in Supplementary Figure S18B we observed good correlation for both types of agreement and no systematic type of bias was detected. Therefore no platform correction was performed for the metagenes analyzed in our study.

5. Survival analyses

Follow up data were available for 2348 of the total 3488 samples and 327 of the 579 TNBC samples (given lack of follow up data in 12 datasets, see Supplementary Table S1). All survival intervals were measured from the time of surgery to the distinct survival endpoint used in the individual datasets. For 11 datasets relapse free survival (RFS) was used as an endpoint (n=1429 total, n=167 TNBC) while for 6 dataset only distant metastasis free survival (DMFS) was available. Thus any local recurrence events are missing from these 6 datasets. In the conduct of the presented analysis event free survival (EFS) was calculated as preferentially corresponding to the RFS endpoint, but measured with respect to the DMFS endpoint if RFS was not available. We have previously shown[C] that the effect of using these different endpoints was rather small in the overall dataset. However, all results from survival analyses were verified by examining the effect of the different endpoints in stratified analyses. Follow up data for those women in whom the envisaged end point was not reached were censored as of the last follow-up date or at 120 months. Subjects with missing values were excluded from the analyses. We constructed Kaplan-Meier curves and used the log-rank test to determine the univariate significance of the variables. Cox regression analysis was applied to analyze the univariate hazard ratio of individual metagenes as continous factors. A Cox proportional-hazards model was used to simultaneously examine the effects of multiple covariates on survival. The effect of each individual variable was assessed with the use of the Wald test and described by the hazard ratio, with

a 95 percent confidence interval (95% CI). In stepwise backward selection models variables were excluded using P=0.05 as cutoff.

6. Development of a prognostic predictor from the IL-8 and B-Cell metagenes in the training cohort-A

As shown in Suppl. Figures S16 and S17 we used both an unsupervised and a supervised approach (next section) to develop prognostic predictors for TNBC. In the unsupervised approach three metagenes (IL-8, Histone, and B-Cell metagenes) revealed independent prognostic value in multivariate cox regression in the finding cohort-A. Since IL-8 and Histone metagenes are positively correlated and both inversely related to the B-Cell metagene we aimed for constructing a simple prognostic predictor for TNBC from a combination of the IL-8 and B-Cell metagenes. Cutoffs for dichotomizing of the IL-8 and B-Cell metagene were optimized stepwise (0.001) in the finding cohort-A as shown in Suppl. Figure S19. Those cutoffs were selected which displayed (i) high significance in univariate Cox regression concurrently with (ii) mostly equally sized sample groups.

Combination of the two dichotomized metagene variables were applied to the finding cohort-A to obtain a binary prognostic predictor. TNBC patients with tumors displaying both a high expression of the B-Cell metagene and low expression of the IL8 metagene displayed a superior prognosis compared to the remaining samples (Figure 4).

7. Supervised prognostic signature generation by SAM

In an independent approach we also applied a supervised classification using all genes on the Affymetrix microarrays to identify a prognostic gene expression signature (right part of the diagram in Supplementary Figure S13). The *Cox* score option of *Significance Analysis of Microarrays* (SAM)[AA] using the R-package *samr* was applied to the finding cohort-A (297 TNBC samples with follow up) as training set. A delta value of 0.3 resulted in 235 probesets associated with poor prognosis and 29 probesets associated with good prognosis with a median false discovery rate of 25%. A more

stringent delta value of 0.5 resulted in 26 probesets associated with poor prognosis with a median false discovery rate < 3.5% (no probesets associated with good prognosis were identified using this higher stringency. The 235 and 29 probesets, respectively, are listed in Supplementary Table S8. This Table also gives information which 26 probesets were obtained by higher stringency. Interestingly, the two probesets of IL-8 on the Affymetrix array are ranked at position 1 and 4 in the poor prognosis probeset list. On the other hand, most of the 29 probesets in the good prognosis probeset list are associated with immune cells.

Supervised prognostic signatures were derived as a compound covariate predictor using each probesets' expression value and the respective SAM-Score as a weight. The results of Kaplan-Meier analysis using a median split of the cohorts according to the supervised prognostic signatures for both the 264 probesets signature (lower stringency) and the 26 probeset signature (higher stringency) are given in Supplementary Figure S14 A-C and E-G, respectively, for all three cohorts.

As expected the 264-SAM-derived-probes prognostic signature had a high prognostic value in the training-set (Supplementary Figure S14A). In contrast, only a trend towards a better prognosis was observed in the validation cohorts-B and –C (Supplementary Figures S14B and S14C). When the 264-SAM-derived-probes prognostic signature score as a continous variable was clustered together with the metagenes from Figure 1 the highest correlation was observed to the cluster containing IL-8, Histone, and VEGF metagenes in both finding cohort-A (Supplementary Figure S14D) and validation cohorts (not shown).

The prognostic value of the signature derived from higher stringency (26-SAM-derived-probes) was analysed in Suppl. Figure S14 panels (E), (F), and (G), respectively. A significant difference in prognosis was found for validation cohort B (panel F) but only a trend for validation cohort C (panel G). The cluster analysis in panel (H) demonstrates that this 26-SAM-derived-probes signature displayed the highest correlation to the IL-8 metagene (the same result was obtained using validation cohorts–B and –C; not shown).

8. Centroid-based definition of Molecular subtypes

We applied a recently published implementation of different variants of the centroid method to assign breast cancer samples to a molecular subtype[BB]. Detailed information and corresponding R-code can be downloaded from the authors of this study at:

http://rock.icr.ac.uk/collaborations/Mackay/centroid.correlations.Eset/ExpressionSet%20Nearest%20Centroid%20Correlations.pdf

For the results presented in Supplementary Table S6 we performed Spearman's rank correlations on all probes both with centering and without centering using the centroids according to Hu et al. [CC] downloaded from

http://rock.icr.ac.uk/collaborations/Mackay/centroid.correlations.Eset/Hu306.centroids.txt

The analyses were performed independently in seven larger datasets (Frankfurt, Mainz, NewYork, Stockholm, Transbig, Uppsala, Rotterdam) to assign a total of 1364 breast cancer samples to a molecular subtype. Subsequently the resulting subtype definitions of the 172 TNBC samples from these datasets were compared to the BLBC vs. Non-BLBC definition deduced from the distribution of the Basal-like metagene.

In addition, the analysis was also performed using the complete cohorts-A and –B of 579 TNBC samples only (without Non-TNBC subtypes). In this case only the variant of the method without centering was applied since centering of a complete ER negative cohort results in distortion of the data as previously shown[DD],[C].

9. Definition of molecular subtypes based on bimodal metagene distributions

Cutoffs for bimodally expressed metagenes (*Basal-like*, *Apocrine*, *Claudin-CD24*) were derived from fitting a mixture of two normal gaussian distributions to the observed distributions by maximum

likelihood optimization using the optim function in R as described by Venables and Ripley[EE]. The resulting cutoffs are shown in Supplementary Figure 3 (*Basal-like*) and Supplementary Figure S6B (*Apocrine*, *Claudin-CD24*). These cutoff values were subsequently used to categorize TNBC samples into the following subgroups: *Basal-like*, *molecular-Apocrine*, and *Claudin-Low*. Any samples classified to more than one group using the distribution-derived cutoff values were assigned as "*unclassified/ambigous*". Among the 394 TNBC samples from the finding cohort-A we detected 249 *Basal-like* (63.2%), 65 *Molecular-Apocrine* (16.5%), and 25 *Claudin-Low* (6.3%) samples by this method. 55 samples (14.0%) were assigned to the "unclassified/ambigous" group. The relationship of the three metagenes is demonstrated in the scatter plot in Supplementary Figure S6A. Most of the samples classified by this method as *Molecular-Apocrine* or *Claudin-Low* were either assigned to the "unclassified" or the "basal-like" group when the centroid method was applied (60-80% depending on the specific variant of the method as given in the preceding section above).

High expression of Adipocyte markers and low expression of proliferation markers are features of both normal tissue as well as the so called "*normal-like*" subtype of breast cancer. Using the above method we were not able to discriminate a "*normal-like*" subtype since the *Adipocyte* metagene and the *Proliferation* metagene did not display bimodal distributions. Similarily immune metagenes displayed a continous distribution of expression values suggesting that these markers rather describe mixtures of cells than subtypes of distinct origin.

10. Immunohistochemical analysis

To analyze the cellular source of expression in samples which show a high expression of the B-cell and IL-8 metagenes we performed immunohistochemistry using specific antibodies. CD20 (clone B-Ly1, Dianova, Hamburg, Germany) was used as marker for B lymphocytes. A polyclonal IL-8 (AF-208-NA) was obtained from R&D Systems (Minneapolis, MN). Briefly, paraffin sections (2 µm) were mounted on Superfrost Plus slides, dewaxed in xylene and rehydrated through graduated ethanol to

water. Antigens were retrieved by microwaving sections in 1 mM EDTA (pH 8.0) for 20 min at 800 W. Blocking was performed using antibody dilution buffer (DCSDiagnostics, Hamburg, Germany) at room temperature for 15 min. Subsequently, antibodies were diluted 1:100 individually in this buffer. Sections were incubated with antibodies for 1 h at room temperature. For negative controls, the primary antibodies were replaced with phosphate-buffered saline. For secondary antibody incubations and detection, the Dako REAL Detection System Alkaline Phosphatase/RED (Dako, Glostrup, Denmark) was used following the protocol of the supplier and sections were counterstained with Mayer's hematoxylin.

11. Relationship of B-Cell and IL-8 metagenes to the medullary subtype of TNBC

The good prognosis of TNBC with high lymphocyte content is in line with properties of medullary breast cancer, a tumor subtype with high amounts of immune cell infiltrates and favourable prognosis. Since this subtype represents <3% of all breast cancers it could account only for a tiny minority of TNBC in our dataset. Nevertheless, we analyzed a publicly available microarray dataset[FF] of medullary and ductal TNBC for expression of B-Cell and IL-8 metagenes. As shown in Suppl. Figure S20 the expression of the metagenes largely overlap between the two histologically defined cancer subtypes and thus the prognostic value of the predictor cannot be explained by the identification medullary breast cancer samples alone.

12. Relationship of the B-Cell/IL-8 prognostic predictor to proliferation in TNBC

As shown in Supplementary Figure S21 no difference in the expression of the proliferation metagene was observed when TNBC samples were stratified according to the prognostic predictor based on high expression of the B-Cell metagene and low expression of the IL-8 metagene.

13. Relationship of previously published gene signatures to the metagenes detected within TNBC.

The correlation of several published gene signatures with the metagenes discovered within the pure TNBC cohort was analyzed by calculating the Pearson correlation coefficient between signature scores in the finding cohort of TNBC. The following gene signatures were included in this analysis: Recurrence score[GG], genomic grade index[HH], Amsterdam signature[II], wound response signature[JJ], 7-gene immune response module[KK], stroma derived prognostic predictor[LL], and a medullary like signature[G]. The *genefu* R-package[MM,NN] was used to calculate signature score as continuous variables. The correlations between these gene signatures and all 16 identified metagenes in TNBC were visualized through hierarchical clustering.

Supplementary Methods References:

[A] McShane LM, Altman DG, Sauerbrei W, Taube SE, Gion M, Clark GM; Statistics Subcommittee of the NCI-EORTC Working Group on Cancer Diagnostics. Reporting recommendations for tumor marker prognostic studies. J Clin Oncol. 2005 Dec 20;23(36):9067-72.

[B] Gong Y, Yan K, Lin F, Anderson K, Sotiriou C, Andre F, Holmes FA, Valero V, Booser D, Pippen JE Jr, Vukelja S, Gomez H, Mejia J, Barajas LJ, Hess KR, Sneige N, Hortobagyi GN, Pusztai L, Symmans WF (2007) Determination of oestrogen-receptor status and ERBB2 status of breast carcinoma: a gene-expression profiling study. Lancet Oncol 8(3):203–211.

[C] Karn T, Metzler D, Ruckhäberle E, Hanker L, Gätje R, Solbach C, Ahr A, Schmidt M, Holtrich U, Kaufmann M, Rody A. Data driven derivation of cutoffs from a pool of 3,030 Affymetrix arrays to stratify distinct clinical types of breast cancer. Breast Cancer Res Treat. 2009 May 20.

[D] Affymetrix (2001) Statistical algorithms reference guide, Technical report, Affymetrix.

[E] Gautier L, Cope L, Bolstad BM, Irizarry RA. affy--analysis of Affymetrix GeneChip data at the probe level. Bioinformatics. 2004 Feb 12;20(3):307-15.

[F] Gentleman RC, Carey VJ, Bates DM, Bolstad B, Dettling M, Dudoit S, Ellis B, Gautier L, Ge Y, Gentry J, Hornik K, Hothorn T, Huber W, Iacus S, Irizarry R, Leisch F, Li C, Maechler M, Rossini AJ, Sawitzki G, Smith C, Smyth G, Tierney L, Yang JY, Zhang J. Bioconductor: open software development for computational biology and bioinformatics. Genome Biol. 2004;5(10):R80.

[G] Sabatier R, Finetti P, Cervera N, Lambaudie E, Esterni B, Mamessier E, Tallet A, Chabannon C, Extra JM, Jacquemier J, Viens P, Birnbaum D, Bertucci F. A gene expression signature identifies two prognostic subgroups of basal breast cancer. Breast Cancer Res Treat. 2010 May 21. [Epub ahead of print]

[H] Simon R, Radmacher MD, Dobbin K, McShane LM. Pitfalls in the use of DNA microarray data for diagnostic and prognostic classification. J Natl Cancer Inst. 2003 Jan 1;95(1):14-8.

[I] Rody A, Holtrich U, Pusztai L, Liedtke C, Gaetje R, Ruckhaeberle E, Solbach C, Hanker L, Ahr A, Metzler D, Engels K, Karn T, Kaufmann M. T-cell metagene predicts a favorable prognosis in estrogen receptor-negative and HER2-positive breast cancers. Breast Cancer Res. 2009;11(2):R15.

[J] Perou CM, Sørlie T, Eisen MB, van de Rijn M, Jeffrey SS, Rees CA, Pollack JR, Ross DT, Johnsen H, Akslen LA, Fluge O, Pergamenschikov A, Williams C, Zhu SX, Lønning PE, Børresen-Dale AL, Brown PO, Botstein D. Molecular portraits of human breast tumours. Nature. 2000 Aug 17;406(6797):747-52.

[K] Farmer P, Bonnefoi H, Becette V, Tubiana-Hulin M, Fumoleau P, Larsimont D, Macgrogan G, Bergh J, Cameron D, Goldstein D, Duss S, Nicoulaz AL, Brisken C, Fiche M, Delorenzi M, Iggo R. Identification of molecular apocrine breast tumours by microarray analysis. Oncogene. 2005 Jul 7;24(29):4660-71.

[L] Doane AS, Danso M, Lal P, Donaton M, Zhang L, Hudis C, Gerald WL. An estrogen receptor-negative breast cancer subset characterized by a hormonally regulated transcriptional program and response to androgen. Oncogene. 2006 Jun 29;25(28):3994-4008.

[M] Farmer P, Bonnefoi H, Anderle P, Cameron D, Wirapati P, Becette V, André S, Piccart M, Campone M, Brain E, Macgrogan G, Petit T, Jassem J, Bibeau F, Blot E, Bogaerts J, Aguet M, Bergh J, Iggo R, Delorenzi M. A stroma-related gene signature predicts resistance to neoadjuvant chemotherapy in breast cancer. Nat Med. 2009 Jan;15(1):68-74.

[N] Bianchini G, Qi Y, Alvarez RH, Iwamoto T, Coutant C, Ibrahim NK, Valero V, Cristofanilli M, Green MC, Radvanyi L, Hatzis C, Hortobagyi GN, Andre F, Gianni L, Symmans WF, Pusztai L. Molecular anatomy of breast cancer stroma and its prognostic value in estrogen receptor-positive and -negative cancers. J Clin Oncol. 2010 Oct 1;28(28):4316-23.

[O] Hennessy BT, Gonzalez-Angulo AM, Stemke-Hale K, Gilcrease MZ, Krishnamurthy S, Lee JS, Fridlyand J, Sahin A, Agarwal R, Joy C, Liu W, Stivers D, Baggerly K, Carey M, Lluch A, Monteagudo C, He X, Weigman V, Fan C, Palazzo J, Hortobagyi GN, Nolden LK, Wang NJ, Valero V, Gray JW, Perou CM, Mills GB. Characterization of a

naturally occurring breast cancer subset enriched in epithelial-to-mesenchymal transition and stem cell characteristics. Cancer Res. 2009 May 15;69(10):4116-24.

[P] Creighton CJ, Li X, Landis M, Dixon JM, Neumeister VM, Sjolund A, Rimm DL, Wong H, Rodriguez A, Herschkowitz JI, Fan C, Zhang X, He X, Pavlick A, Gutierrez MC, Renshaw L, Larionov AA, Faratian D, Hilsenbeck SG, Perou CM, Lewis MT, Rosen JM, Chang JC. Residual breast cancers after conventional therapy display mesenchymal as well as tumor-initiating features. Proc Natl Acad Sci U S A. 2009 Aug 18;106(33):13820-5.

[Q] Perou CM, Jeffrey SS, van de Rijn M, Rees CA, Eisen MB, Ross DT, Pergamenschikov A, Williams CF, Zhu SX, Lee JC, Lashkari D, Shalon D, Brown PO, Botstein D. Distinctive gene expression patterns in human mammary epithelial cells and breast cancers. Proc Natl Acad Sci U S A. 1999 Aug 3;96(16):9212-7.

[R] Palmer C, Diehn M, Alizadeh AA, Brown PO. Cell-type specific gene expression profiles of leukocytes in human peripheral blood. BMC Genomics. 2006 May 16;7:115.

[S] Desmedt C, Haibe-Kains B, Wirapati P, Buyse M, Larsimont D, Bontempi G, Delorenzi M, Piccart M, Sotiriou C. Biological processes associated with breast cancer clinical outcome depend on the molecular subtypes. Clin Cancer Res. 2008 Aug 15;14(16):5158-65.

[T] Schmidt M, Böhm D, von Törne C, Steiner E, Puhl A, Pilch H, Lehr HA, Hengstler JG, Kölbl H, Gehrmann M. The humoral immune system has a key prognostic impact in node-negative breast cancer. Cancer Res. 2008 Jul 1;68(13):5405-13.

[U] Alexe G, Dalgin GS, Scanfeld D, Tamayo P, Mesirov JP, DeLisi C, Harris L, Barnard N, Martel M, Levine AJ, Ganesan S, Bhanot G, High expression of lymphocyte-associated genes in node-negative HER2+ breast cancers correlates with lower recurrence rates, Cancer Res. 2007 Nov 15;67(22):10669-76

[V] Hu Z, Fan C, Livasy C, He X, Oh DS, Ewend MG, Carey LA, Subramanian S, West R, Ikpatt F, Olopade OI, van de Rijn M, Perou CM. A compact VEGF signature associated with distant metastases and poor outcomes. BMC Med. 2009 Mar 16;7:9.

[W] Waugh DJ, Wilson C. The interleukin-8 pathway in cancer. Clin Cancer Res. 2008 Nov 1;14(21):6735-41.

[X] Angelo LS, Kurzrock R. Vascular endothelial growth factor and its relationship to inflammatory mediators. Clin Cancer Res. 2007 May 15;13(10):2825-30.

[Y] Bièche I, Chavey C, Andrieu C, Busson M, Vacher S, Le Corre L, Guinebretière JM, Burlinchon S, Lidereau R, Lazennec G. CXC chemokines located in the 4q21 region are up-regulated in breast cancer. Endocr Relat Cancer. 2007 Dec;14(4):1039-52.

[Z] Symmans WF, Hatzis C, Sotiriou C, Andre F, Peintinger F, Regitnig P, Daxenbichler G, Desmedt C, Domont J, Marth C, Delaloge S, Bauernhofer T, Valero V, Booser DJ, Hortobagyi GN, Pusztai L. Genomic index of sensitivity to endocrine therapy for breast cancer. J Clin Oncol. 2010 Sep 20;28(27):4111-9.

[AA] Tusher VG, Tibshirani R, Chu G. Significance analysis of microarrays applied to the ionizing radiation response. Proc Natl Acad Sci U S A. 2001 Apr 24;98(9):5116-21.

[BB] Weigelt B, Mackay A, A'hern R, Natrajan R, Tan DS, Dowsett M, Ashworth A, Reis-Filho JS. Breast cancer molecular profiling with single sample predictors: a retrospective analysis. Lancet Oncol. 2010 Apr;11(4):339-49.

[CC] Hu Z, Fan C, Oh DS, Marron JS, He X, Qaqish BF, Livasy C, Carey LA, Reynolds E, Dressler L, Nobel A, Parker J, Ewend MG, Sawyer LR, Wu J, Liu Y, Nanda R, Tretiakova M, Ruiz Orrico A, Dreher D, Palazzo JP, Perreard L, Nelson E, Mone M, Hansen H, Mullins M, Quackenbush JF, Ellis MJ, Olopade OI, Bernard PS, Perou CM. The molecular portraits of breast tumors are conserved across microarray platforms. BMC Genomics. 2006 Apr 27;7:96.

[DD] Lusa L, McShane LM, Reid JF, De Cecco L, Ambrogi F, Biganzoli E, Gariboldi M, Pierotti MA. Challenges in projecting clustering results across gene expression-profiling datasets. J Natl Cancer Inst. 2007 Nov 21;99(22):1715-23.

[EE] Venables WN, Ripley BD (2002) Modern Applied Statistics with S, chap 16.3, 4th edn. Springer. ISBN 0-387-95457-0.

[FF] Bertucci F, Finetti P, Cervera N, Charafe-Jauffret E, Mamessier E, Adélaïde J, Debono S, Houvenaeghel G, Maraninchi D, Viens P, Charpin C, Jacquemier J, Birnbaum D. Gene expression profiling shows medullary breast cancer is a subgroup of basal breast cancers. Cancer Res. 2006 May 1;66(9):4636-44.

[GG] Paik S, Shak S, Tang G, Kim C, Baker J, Cronin M, Baehner FL, Walker MG, Watson D, Park T, Hiller W, Fisher ER, Wickerham DL, Bryant J, Wolmark N. A multigene assay to predict recurrence of tamoxifen-treated, node-negative breast cancer. N Engl J Med. 2004 Dec 30;351(27):2817-26. Epub 2004 Dec 10.

[HH] Sotiriou C, Wirapati P, Loi S, Harris A, Fox S, Smeds J, Nordgren H, Farmer P, Praz V, Haibe-Kains B, Desmedt C, Larsimont D, Cardoso F, Peterse H, Nuyten D, Buyse M, Van de Vijver MJ, Bergh J, Piccart M, Delorenzi M. Gene expression profiling in breast cancer: understanding the molecular basis of histologic grade to improve prognosis. J Natl Cancer Inst. 2006 Feb 15;98(4):262-72.

[II] van 't Veer LJ, Dai H, van de Vijver MJ, He YD, Hart AA, Mao M, Peterse HL, van der Kooy K, Marton MJ, Witteveen AT, Schreiber GJ, Kerkhoven RM, Roberts C, Linsley PS, Bernards R, Friend SH. Gene expression profiling predicts clinical outcome of breast cancer. Nature. 2002 Jan 31;415(6871):530-6.

[JJ] Chang HY, Sneddon JB, Alizadeh AA, Sood R, West RB, Montgomery K, Chi JT, van de Rijn M, Botstein D, Brown PO. Gene expression signature of fibroblast serum response predicts human cancer progression: similarities between tumors and wounds. PLoS Biol. 2004 Feb;2(2):E7. Epub 2004 Jan 13.

[KK] Teschendorff AE, Miremadi A, Pinder SE, Ellis IO, Caldas C. An immune response gene expression module identifies a good prognosis subtype in estrogen receptor negative breast cancer. Genome Biol. 2007;8(8):R157.

[LL] Finak G, Bertos N, Pepin F, Sadekova S, Souleimanova M, Zhao H, Chen H, Omeroglu G, Meterissian S, Omeroglu A, Hallett M, Park M. Stromal gene expression predicts clinical outcome in breast cancer. Nat Med. 2008 May;14(5):518-27.

[MM] genefu R package: Relevant Functions for Gene Expression Analysis, Especially in Breast Cancer [http://cran.r-project.org/web/packages/genefu/]

[NN] Haibe-Kains B, Desmedt C, Rothé F, Piccart M, Sotiriou C, Bontempi G. A fuzzy gene expression-based computational approach improves breast cancer prognostication. Genome Biol. 2010;11(2):R18. Epub 2010 Feb 15.

Supplementary Figures:

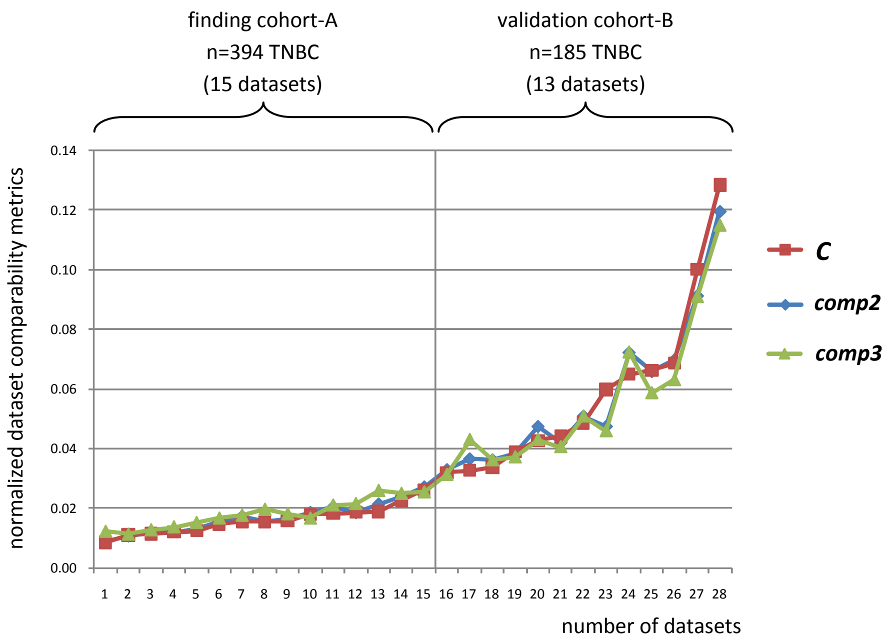

Supplementary Figure S16: Selection methods for comparable Affymetrix datasets

We derived a simple comparability metric *C* from the sum of the squared differences of the mean (μ) within a specific dataset and among all datasets, respectively, normalized by the standard deviation (σ) calculated for all genes (g) on the array:

$$C_{\ dataset_i} = \sum_{g=1}^{n} \left(\frac{\mu_{g,\ dataset_i} - \mu_{g,\ total}}{\sigma_{g,\ total}} \right)^2$$

We also considered a metric without normalizing by the standard deviation:

$$comp2_{\ dataset_i} = \sum_{g=1}^{n} (\mu_{g,\ dataset_i} - \mu_{g,\ total})^2$$

and using the mean of the means among datasets instead of the global mean:

$$comp3_{\ dataset_i} = \sum_{g=1}^{n} (\mu_{g,dataset_i} - \frac{\sum_{k=1}^{m} \mu_{g,\ dataset_k}}{m})^2$$

In the above figure the results of all three methods were compared on a normalized scale. An increase was observed for all three metrics between 0.02 and 0.03. The selected cutoff of $C \leq 0.03$ resulted in the inclusion of 15 datasets encompassing n=394 TNBC samples in the finding cohort-A. The excluded datasets encompassing n=185 samples were withhold as validation cohort-B.

A

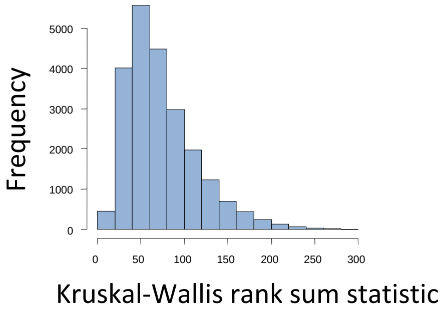

B

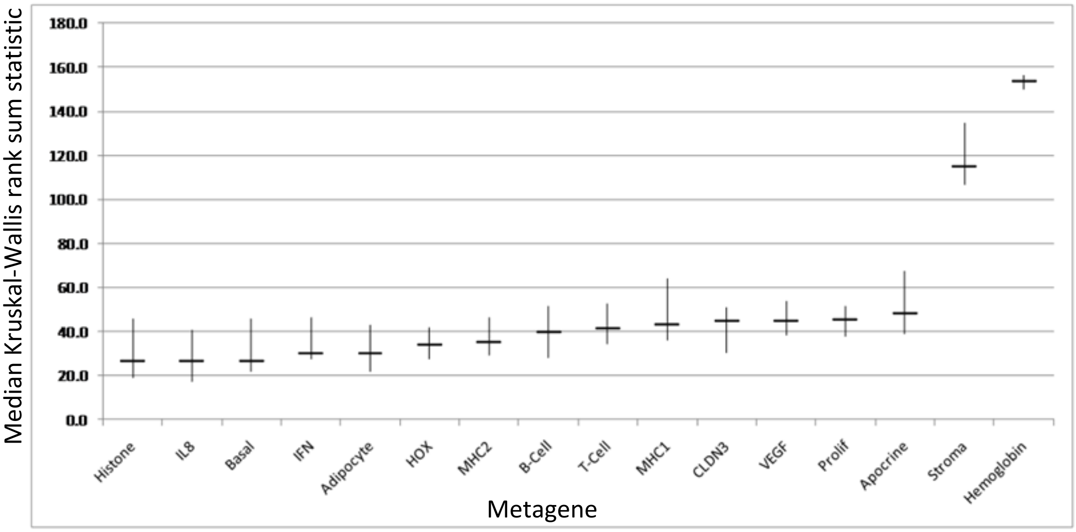

Supplementary Figure S17: Analysis of metagenes for potential dataset bias

A) The standard Kruskal-Wallis rank test was used for the dependence of each individual probeset to the vector of the 15 different datasets in the finding cohort-A of n=394 samples. The distribution of the rank sum statistics for all 22,283 probesets from the U133A array is shown.

B) Median Kruskal-Wallis rank sum statistic (horizontal lines) and inter quartile range (IQR, vertical lines) of the probesets from the 16 metagenes are shown. Only the Stroma and Hemoglobin metagenes display a high bias between datasets. This effect originates from the inclusion of two datasets obtained from FNA samples which contain high amounts of blood and low amounts of stromal tissue.

A

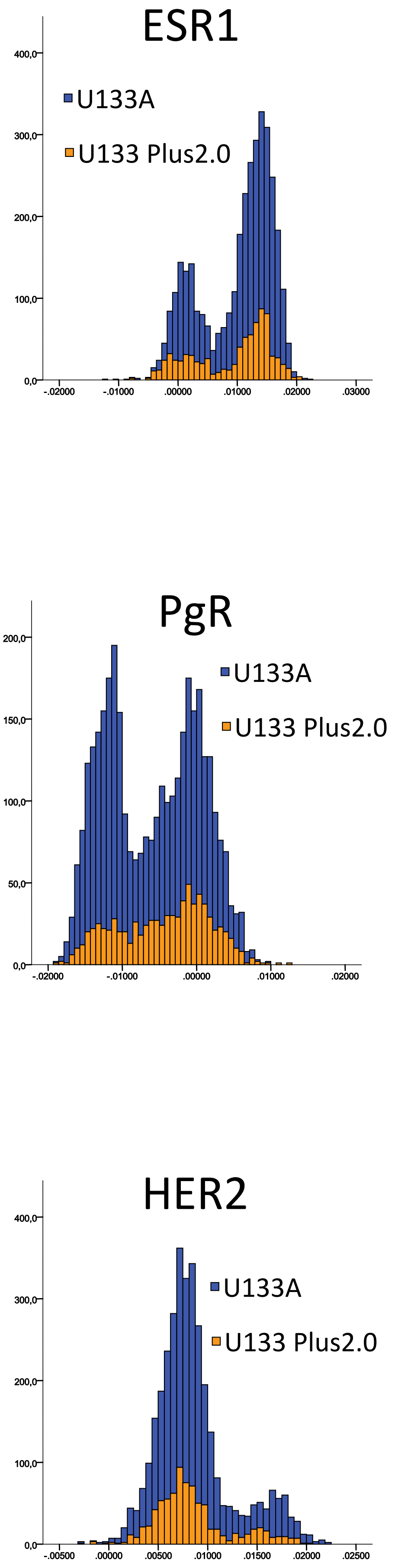
ESR1
U133A
U133 Plus2.0
400,0
300,0
200,0
100,0
0,0
-.02000
-.01000
.00000
.01000
.02000
.03000
PgR
U133A
U133 Plus2.0
200,0
150,0
100,0
50,0
0,0
-.02000
-.01000
.00000
.01000
.02000
HER2
U133A
U133 Plus2.0
400,0
300,0
200,0
100,0
0,0
-.00500
.00000
.00500
.01000
.01500
.02000
.02500

B

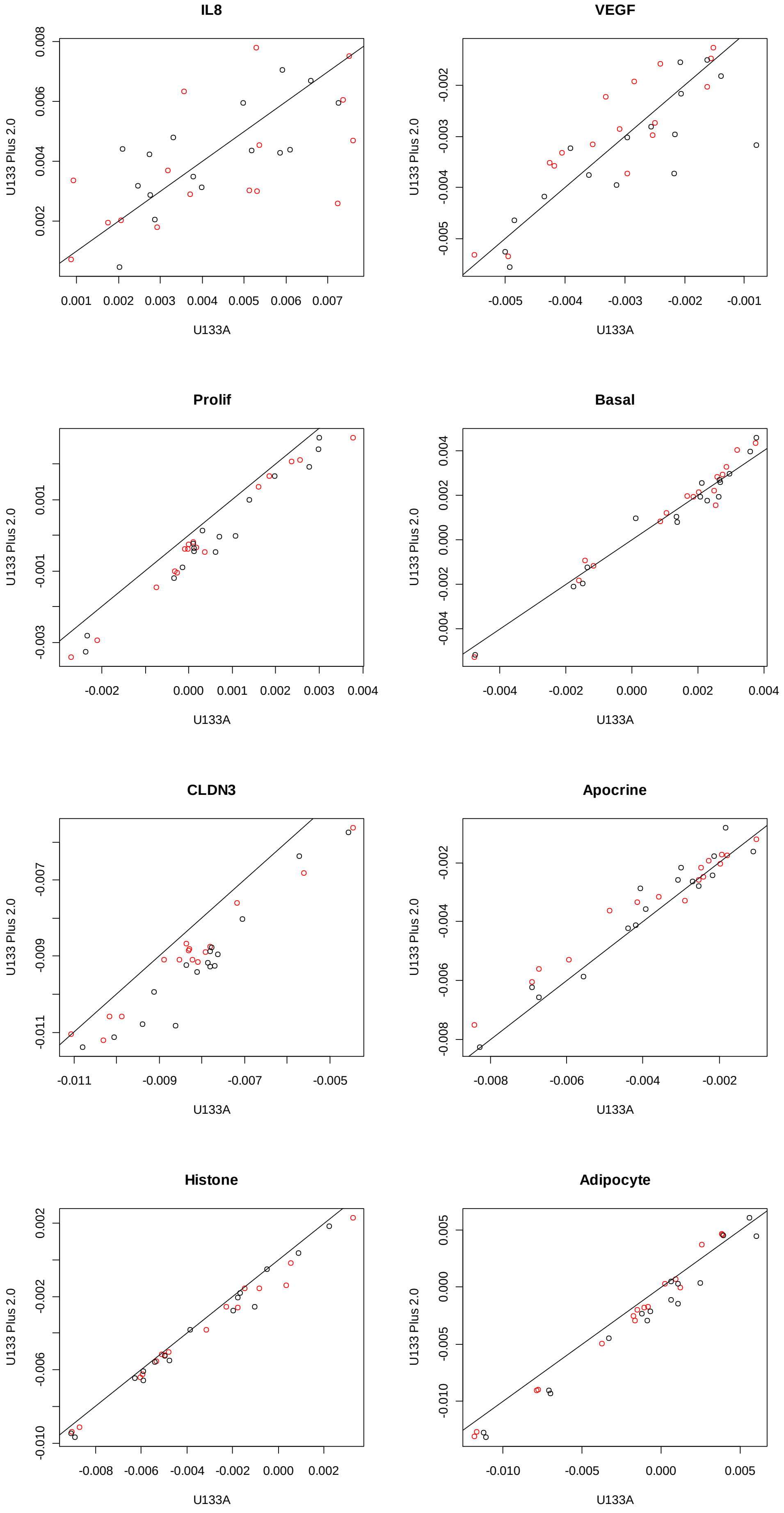
IL8
U133 Plus 2.0
0.002
0.004
0.006
0.008
0.001 0.002 0.003 0.004 0.005 0.006 0.007
U133A
VEGF
U133 Plus 2.0
-0.005
-0.004
-0.003
-0.002
-0.005 -0.004 -0.003 -0.002 -0.001
U133A
Prolif
U133 Plus 2.0
-0.003
-0.001
0.001
-0.002 0.000 0.001 0.002 0.003 0.004
U133A
Basal
U133 Plus 2.0
-0.004
-0.002
0.000
0.002
0.004
-0.004 -0.002 0.000 0.002 0.004
U133A
CLDN3
U133 Plus 2.0
-0.011
-0.009
-0.007
-0.011 -0.009 -0.007 -0.005
U133A
Apocrine
U133 Plus 2.0
-0.008
-0.006
-0.004
-0.002
-0.008 -0.006 -0.004 -0.002
U133A
Histone
U133 Plus 2.0
-0.010
-0.006
-0.002
0.002
-0.008 -0.006 -0.004 -0.002 0.000 0.002
U133A
Adipocyte
U133 Plus 2.0
-0.010
-0.005
0.000
0.005
-0.010 -0.005 0.000 0.005
U133A

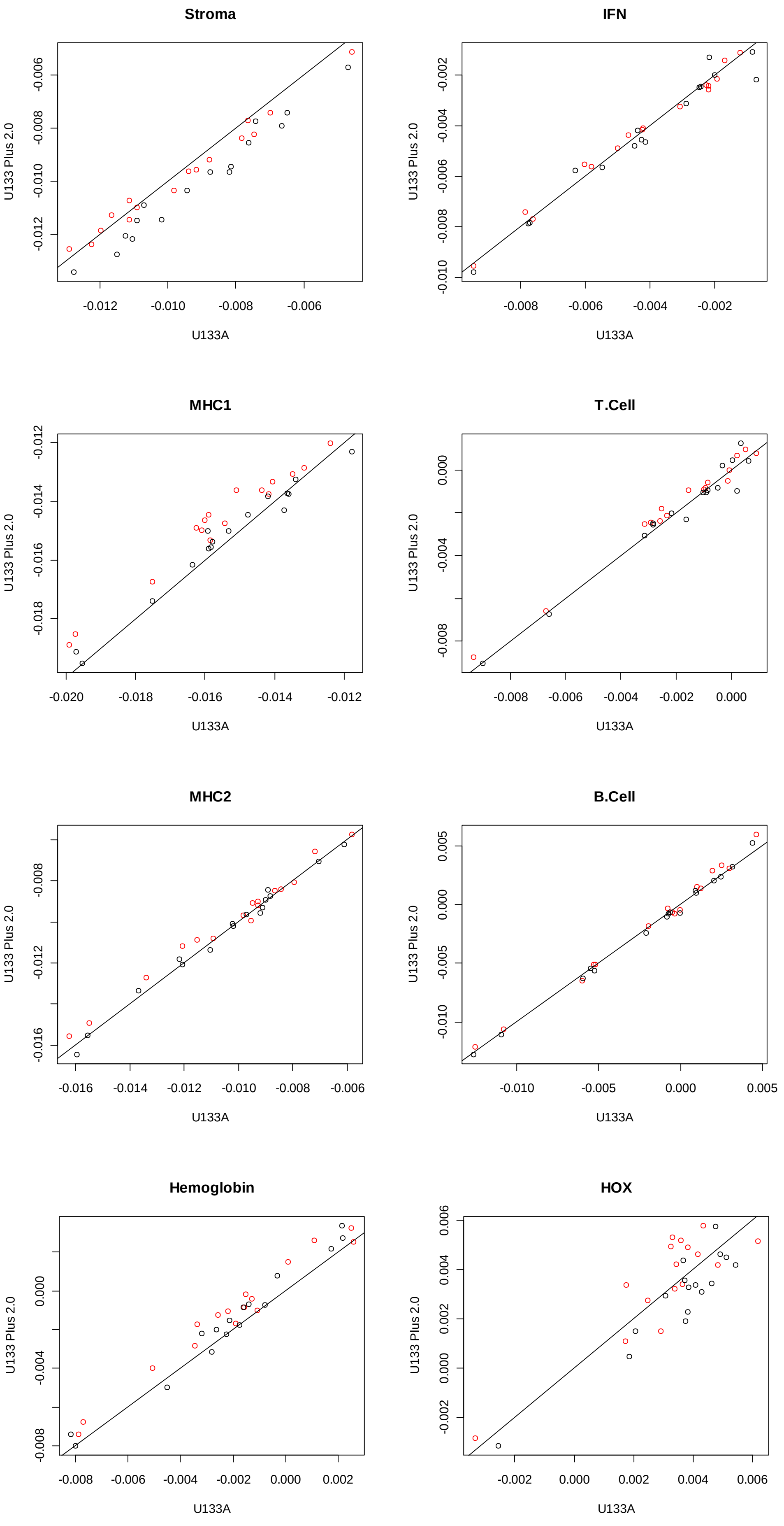
Stroma
U133 Plus 2.0
U133A
-0.006
-0.008
-0.010
-0.012
IFN
-0.002
-0.004
-0.006
-0.008
-0.010
MHC1
-0.012
-0.014
-0.016
-0.018
-0.020
T.Cell
0.000
MHC2
-0.014
-0.016
B.Cell
0.005
0.000
-0.005
-0.010
Hemoglobin
0.002
HOX
0.006
0.004

C

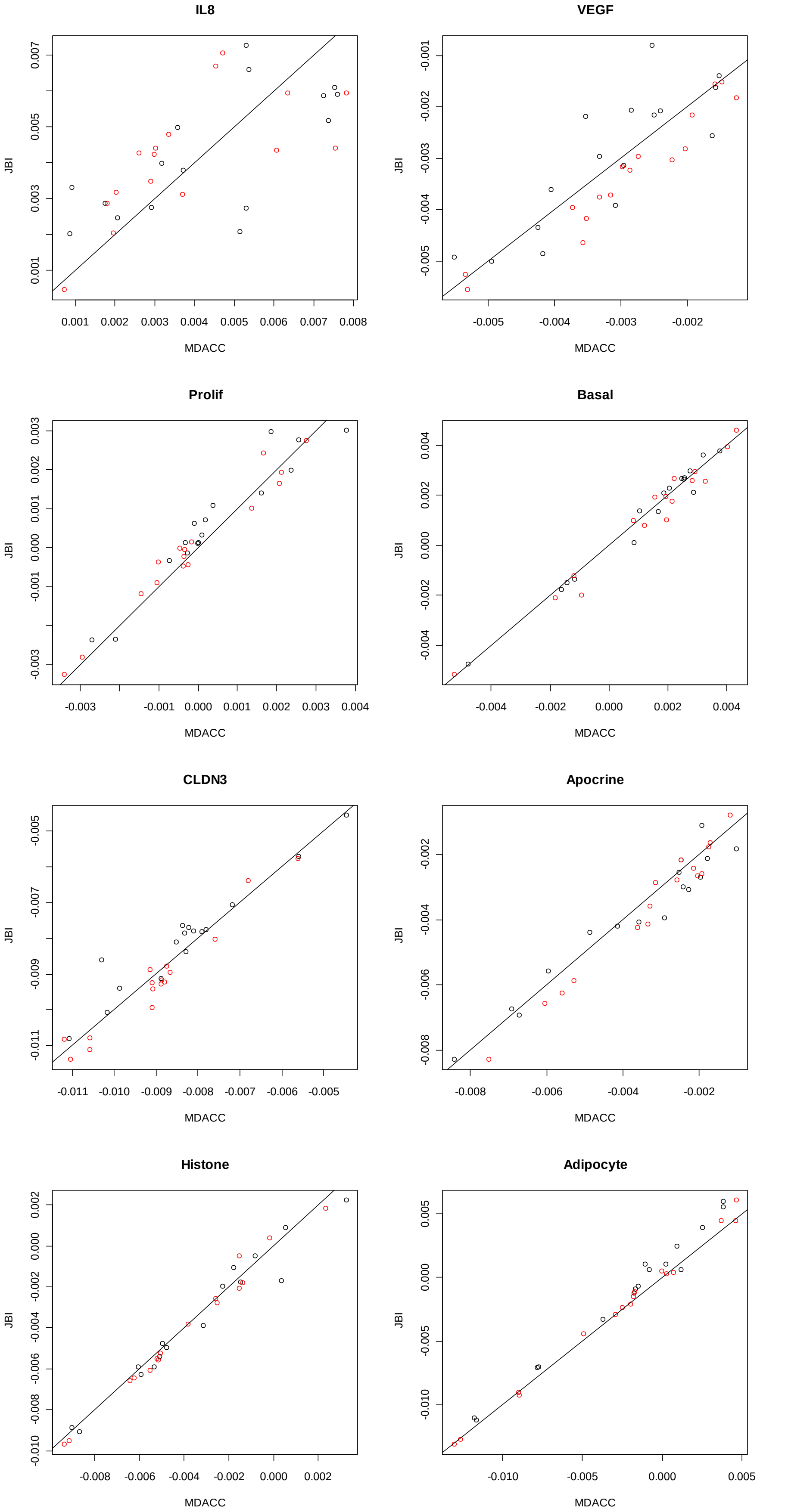

IL8
VEGF
Prolif
Basal
CLDN3
Apocrine
Histone
Adipocyte
JBI
MDACC

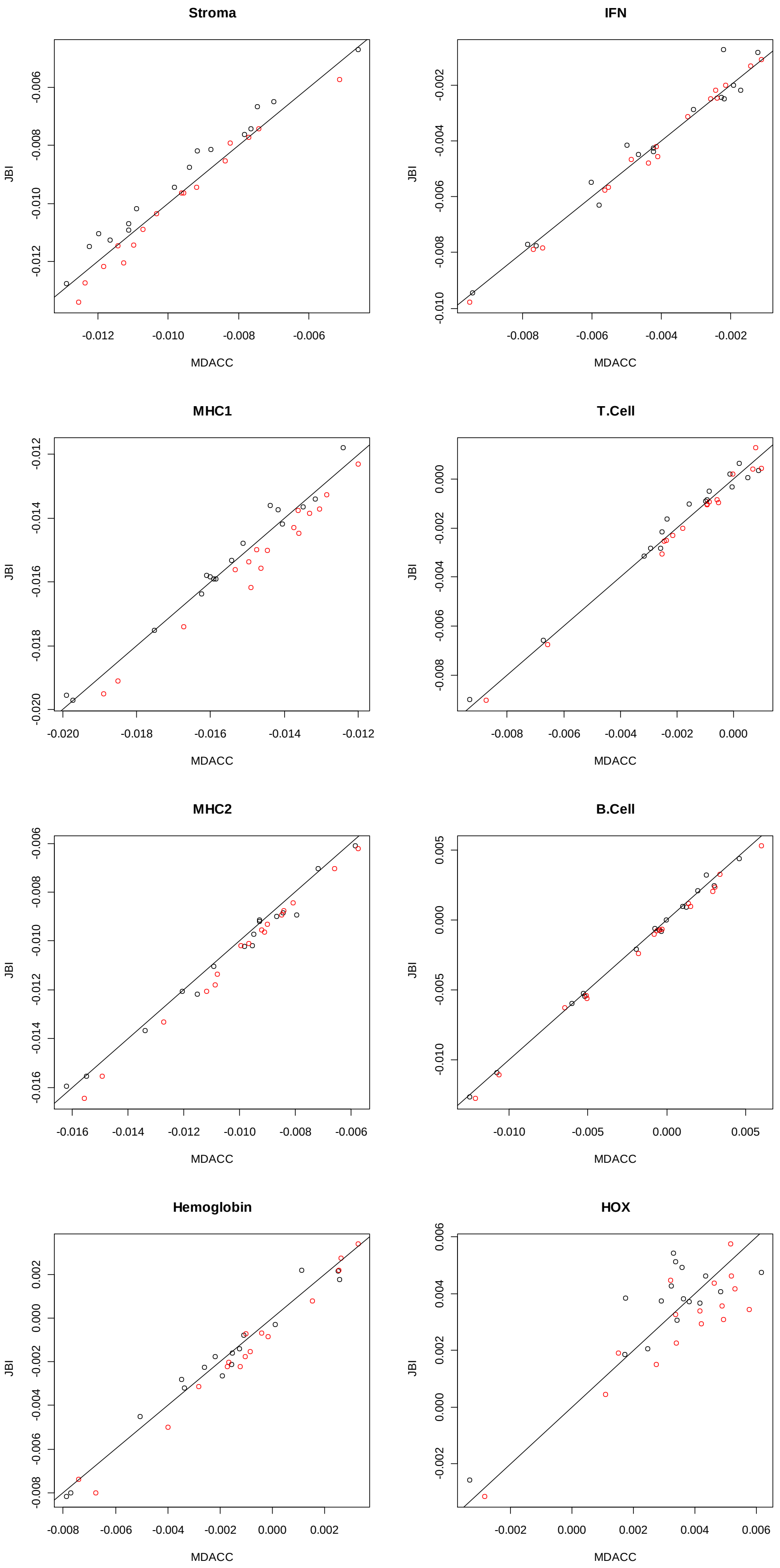
Stroma
JBI
-0.006
-0.008
-0.010
-0.012
-0.012
-0.010
-0.008
-0.006
MDACC
IFN
JBI
-0.002
-0.004
-0.006
-0.008
-0.010
-0.008
-0.006
-0.004
-0.002
MDACC
MHC1
JBI
-0.012
-0.014
-0.016
-0.018
-0.020
-0.020
-0.018
-0.016
-0.014
-0.012
MDACC
T.Cell
JBI
0.000
-0.002
-0.004
-0.006
-0.008
-0.008
-0.006
-0.004
-0.002
0.000
MDACC
MHC2
JBI
-0.006
-0.008
-0.010
-0.012
-0.014
-0.016
-0.016
-0.014
-0.012
-0.010
-0.008
-0.006
MDACC
B.Cell
JBI
0.005
0.000
-0.005
-0.010
-0.010
-0.005
0.000
0.005
MDACC
Hemoglobin
JBI
0.002
0.000
-0.002
-0.004
-0.006
-0.008
-0.008
-0.006
-0.004
-0.002
0.000
0.002
MDACC
HOX
JBI
0.006
0.004
0.002
0.000
-0.002
-0.002
0.000
0.002
0.004
0.006
MDACC

Supplementary Figure S18: Analysis of platform bias between U133A and U133 Plus 2.0 Affymetrix arrays

A) Distribution of gene expression of ESR1 (probeset 205225_at), PgR (probeset 208305_at), and HER2 (probeset 216836_s_at) among 3488 breast cancer samples profiled either on Affymetrix U133A (blue) or U133 Plus 2.0 (orange) arrays. Similar distributions and cutoff values were obtained for both platforms.

B) The inter-platform agreement for the 16 metagenes from our study between Affymetrix U133A and U133 Plus 2.0 arrays was analyzed using the raw data from a 2x2 factorial study from the Symmans et al. 2010 J Clin Oncol. 28:4111 (GEO accession number GSE17700). A good correlation between the two platforms was observed and a systematic bias affecting all metagenes was not detected.

C) The inter-laboratory agreement between two different laboratories (MDA and JBI) for the 16 metagenes from our study was analyzed using the raw data from a 2x2 factorial study from the Symmans et al. 2010 J Clin Oncol. 28:4111 (GEO accession number GSE17700). A good correlation between the two laboratories was observed and a systematic bias affecting all metagenes was not detected.

A

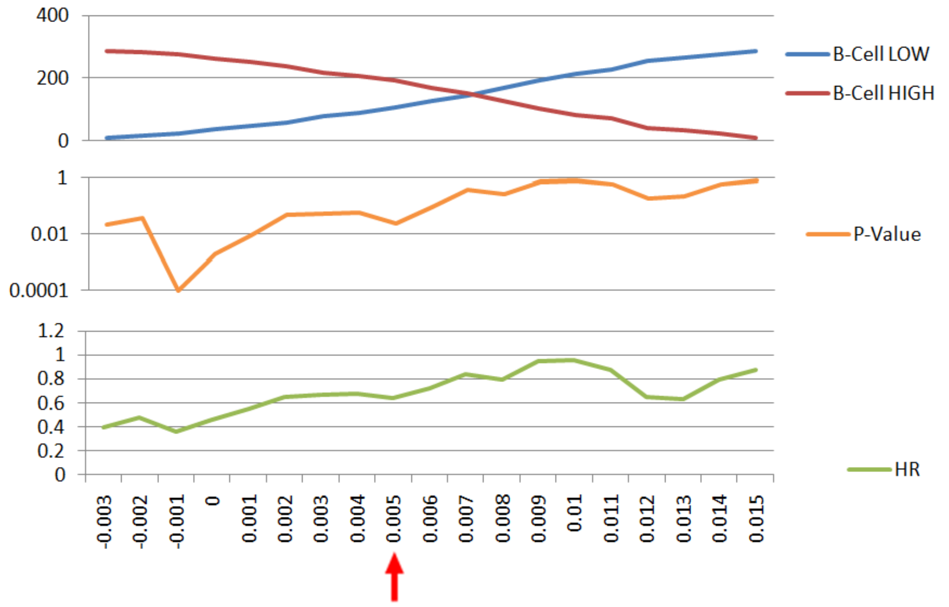

B

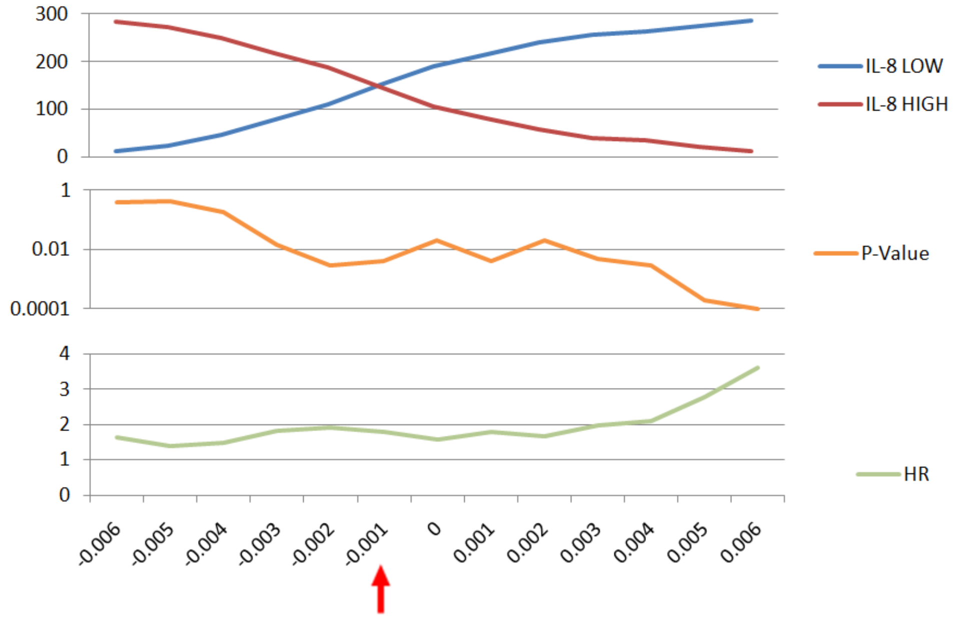

Supplementary Figure S19: Selection of cutoffs for dichotomizing of the IL-8 and B-Cell metagenes in the finding cohort-A

Univariate Cox regression analysis of event free survival was performed in the finding cohort-A with dichotomized IL-8 (in **A**) and B-Cell (in **B**) metagenes, respectively. The results of different cutoff values in steps of 0.001 are shown. In each figure the upper panel shows the number of samples in the two groups according to the used cutoff. The middle and lower panels shows the P-Value and hazard ratio of the respective univariate Cox regression according to the applied cutoff.

Those cutoffs were selected which concurrently displayed (i) a low P-Value and (ii) mostly equally sized sample groups. The cutoffs chosen for all further analyses are marked by red arrows (B-Cell metagene cutoff 0.005 (A) and IL-8 metagene cutoff -0.001 (B), respectively).

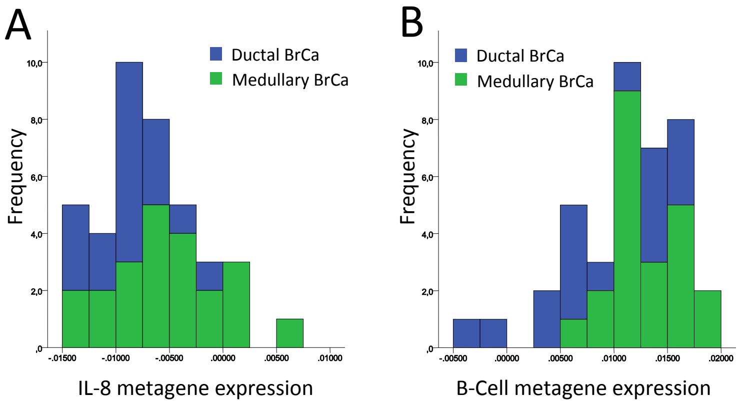

Supplementary Figure S20: Distribution of the IL-8 and B-Cell metagenes in TNBC of the medullary and non-medullary subtype

Affymetrix expression data from 39 triple negative breast cancers of the study of Bertucci et al. (Cancer Res. 2006; 66(9): 4636-44) on medullary breast cancer were analyzed. Based on histopatholocigal analysis 22 of these samples were defined as typical medullary breast cancer in the original study and 17 as ductal breast cancer. All probesets of the IL-8 and B-Cell metagenes were available from the Supplementary Data of this study. Shown is the distribution of the metagene expression values of IL-8 (in A) and B-Cell (in B) metagenes from the for ductal breast cancer (blue) and medullary breast cancer (green) samples.

While the expression of the B-Cell metagene is slightly higher in the medullary cohort no clear difference was observed in IL-8 and B-Cell metagene expression between the two histopathological subtypes.

(*Note that since no complete Affymetrix CEL file data were available for this dataset the scale of the metagene values is not the same as in TNBC cohorts–A,-B,-C, and the cutoff values cannot be directly adapted.*)

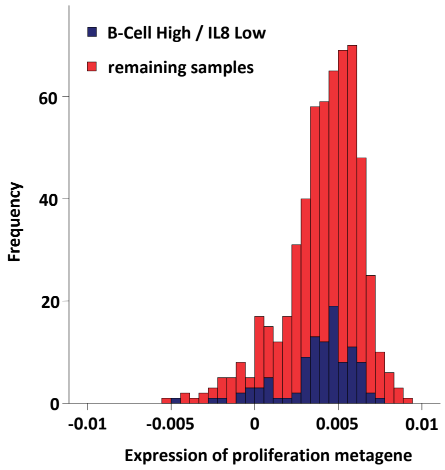

Supplementary Figure S21: **Comparison of the proliferative activity of TNBC stratified according to the combined B-Cell/IL-8 metagenes in cohort-A**

The distribution of the proliferation metagenes is shown for TNBC samples stratified according the combined B-Cell/IL-8 metagene with samples displaying either both high B-Cell and low IL-8 metagene expression in blue and the remaining samples in red, respectively. No difference in the expression of the proliferation metagene is observed between these two groups. Similar results were obtained in the validation cohorts (not shown).
